# Oxidation State Effects on Decoherence in Isovalent 4f^7^ Complexes

**DOI:** 10.1021/acs.inorgchem.6c04187

**Published:** 2026-10-08

**Authors:** Grant R. Wilkinson, Manoj V. H. Subramanya, Maximilian G. Bernbeck, Kavipriya Thangavel, Stephen Hill, Henry S. La Pierre

**Affiliations:** † School of Chemistry and Biochemistry, 1372 Georgia Institute of Technology, Atlanta, Georgia 30332-0400, United States; ‡ National High Magnetic Field Laboratory, 7823 Florida State University, Tallahassee, Florida 32310, United States; § Department of Physics, Florida State University, Tallahassee, Florida 32306, United States; ∥ Department of Chemistry and Biochemistry, Florida State University, Tallahassee, Florida 32306, United States; ⊥ Nuclear and Radiological Engineering and Medical Physics Program, School of Mechanical Engineering, Georgia Institute of Technology, Atlanta Georgia 30332-0400, United States; # Physical Sciences Division, Pacific Northwest National Laboratory, Richland, Washington 99352, United States

## Abstract

The results of pulsed EPR measurements at W-band (94 GHz) on a series of isovalent 4f^7^ ions (Eu^2+^, Gd^3+^, Tb^4+^) are presented, constituting the first example to date of pulsed EPR on molecular Tb^4+^. An inverse correlation is found between the 5 K phase memory relaxation times (*T*_m_) and the degree of zero-field splitting (ZFS) in these complexes. This observation reveals the role of ZFS as an effective reporter not only of structural distortions away from ideal cubic symmetry, but also of the overall electronic structure changes wrought by chemical oxidation, which undermine the seemingly advantageous increases in excited state energies and metal–ligand covalency across the series. Building upon previous studies of the physical properties of high oxidation state lanthanide complexes, these results further highlight the necessity for detailed electronic structure–property relationship studies to fully map the electronic consequences of chemical oxidation in the lanthanides.

## Graphical abstract

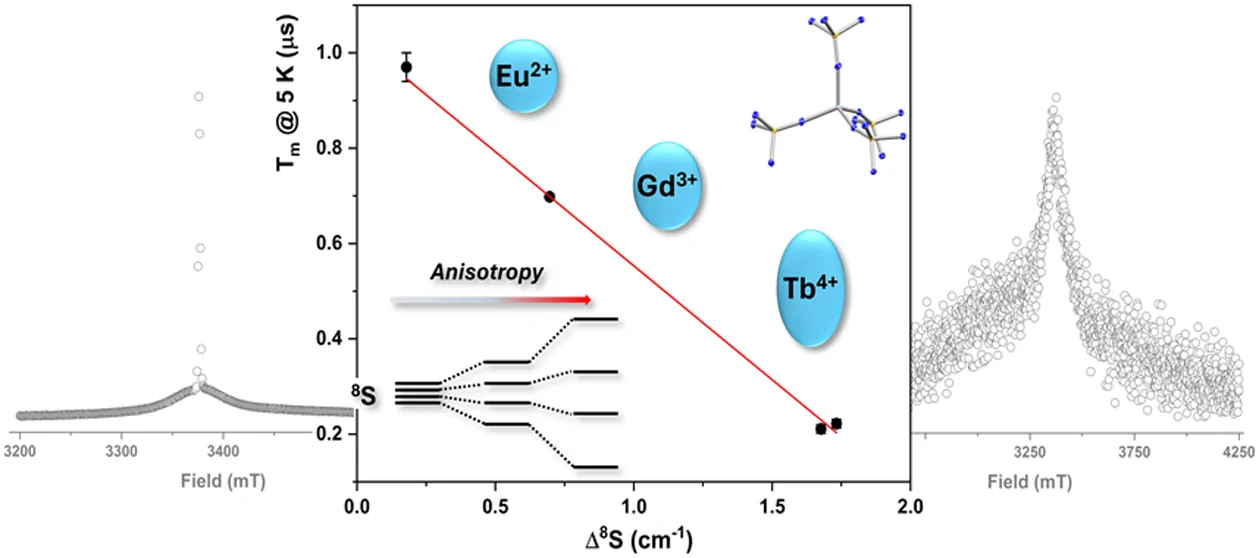

## Introduction

Among molecular complexes of lanthanide ions, Gd^3+^ has demonstrated excellent potential for applications in the design of molecular spin qubits owing to its chemical stability and isotropic valence electron configuration.^1–9^ The 4f^7^ configuration of Gd^3+^ possesses zero orbital angular momentum (and thus first-order spin–orbit coupling is inoperable) and, in general, such isotropic electronic states demonstrate insensitivity to coupled vibrational modes, thus increasing both spin–lattice (*T*_1_) and phase memory (*T*_m_) coherence times (in *T*_1_ limited regimes).^5,10–12^ Despite the advantageous benefits of the 4f^7^ configuration for Gd^3+^, there has been minimal to no extension to the isoelectronic congeners Eu^2+^ and Tb^4+^ in the context of quantum information science.

Though both ions are accessible under the appropriate conditions, the disparate requirements for stabilization of Eu^2+^ and Tb^4+^ in molecular species have precluded detailed studies of the effects of chemical oxidation in an isoelectronic 4f^7^ series on fundamental electronic structure and decoherence properties. This issue is particularly pronounced for Tb^4+^, and high-oxidation state non-Ce^4+^ lanthanide ions in general, where isolable molecular complexes have only been available since 2019, with only a handful of ligand frameworks demonstrating suitable stabilization of these ions to enable detailed physical characterization.^13–26^ The physical properties thus far elucidated for these ions point to distinct changes in fundamental electronic structure as a function of oxidation state, with increases in metal–ligand covalency and crystal field interactions leading to significant perturbation of the free-ion contributions.^27^

For Pr^4+^, recent work in extended solids^28–31^ and molecular complexes^19,21^ demonstrates how the increase in crystal field splitting leads to competition with spin–orbit coupling. This intermediate coupling regime quenches the orbital angular momentum, affording a greater isotropic character for the ion and enhancing its coherence properties.^29,30^ In contrast, the formally isotropic Tb^4+^ gains anisotropy through mixing of excited states into the ground ^8^S_7/2_ state facilitated by second-order spin–orbit coupling and intra-ion spin–spin coupling.^32,33^ This phenomenon is parametrized by the zero-field splitting (ZFS) of the ground octet.

To investigate how modulation of the underlying electronic structure as a function of oxidation state directs ZFS, our group previously reported the only extant series of molecular Eu^2+^, Gd^3+^, and Tb^4+^ ions in a mostly conserved coordination environment.^34^ In this series, where each ion is supported by four [NP­(1,2-bis-*^t^* Bu-diamidoethane)­(NEt_2_)]^−^ ([NP*]^−^) imidophosphorane ligands (Figures 1 and 2), high-frequency/high-field continuous-wave (CW) EPR and complementary theoretical calculations were performed to examine the static electronic properties of these complexes.^34^ In these studies, it was found that ZFS increased moving from Eu^2+^ to Gd^3+^ to Tb^4+^, driven primarily by the increase in second-order spin orbit coupling,^34^ with the increasing spin–orbit coupling constant, ζ, consistent with the increased effective nuclear charge both across the series and with increasing oxidation state.^35^ This occurs despite the increasing energy gap between the ground ^8^S and excited ^6^P state across the series,^34^ which should reduce the degree of state mixing, holding other contributions constant.

**Figure 1:**
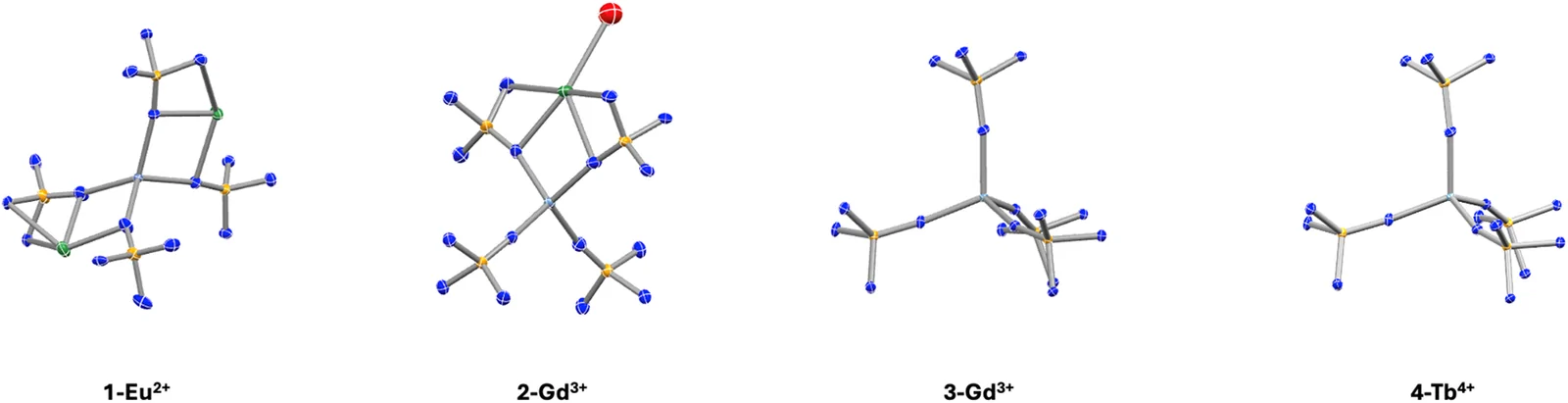
Molecular structures of **1-Eu^2+^**, **2-Gd^3+^**, **3-Gd^3+^**, and **4-Tb^4+^** with thermal ellipsoids set at 50%. Cyan, blue, orange, red, and green represent the lanthanide atom, nitrogen, phosphorus, oxygen, and potassium, respectively. All carbon and hydrogen atoms, along with disordered components and noncoordinated solvents are omitted for clarity.

**Figure 2:**
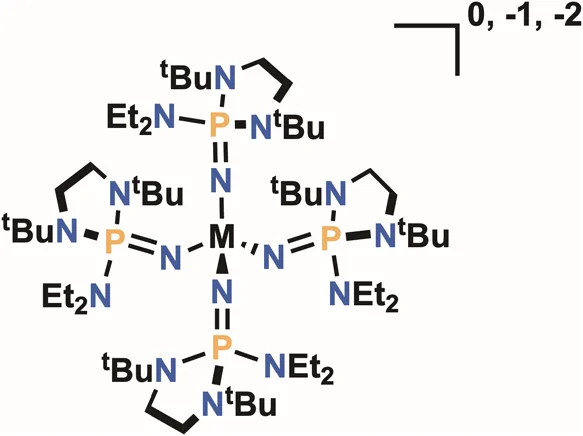
Representative molecular structure of a [M­(NP­(1,2-bis-*^t^* Bu-diamidoethane)­NEt_2_)_4_] complex where M = Eu^2+^, Gd^3+^, or Tb^4+^.

Additionally, the increasing magnitude of the crystal field and metal–ligand bond covalency as a function of increasing oxidation state in these ions serves to magnify the effects of structural distortions away from ideal *T*_d_ symmetry, where ZFS is quenched.^32,33^ This greater crystal field strength results in an increase in the overall splitting of the excited ^6^P state, lowering the effective energy gap to the ground state and further enabling state mixing giving rise to anisotropy.

The impact of the individual components of an ion’s electronic structure on the relaxation properties of qubits has been well studied in transition metals.^36–51^ While several studies have sought to establish optimal criteria for qubits based upon the tuning of one or more of these parameters, competition between individual perturbations has hindered their development and represents a critical tension in the design of molecular spin qubits. For these 4f^7^ ions, this interplay drives their sensitivity to static structural distortions giving rise to anisotropy, which in turn enhances spin–lattice relaxation,^5^ the main driver of *T*_m_ at high temperatures. To further understand the competition between these phenomena in formally isotropic 4f^7^ ions, the resulting coherence properties, and the underlying electronic structure of these ions, we present pulsed electron paramagnetic resonance (EPR) spectroscopy data of an isoelectronic (f^7^), isovalent series of Eu^2+^, Gd^3+^, and Tb^4+^ complexes. These data constitute the first example to date of such experiments reported for molecular Tb^4+^ and establish a correlation between enhanced environmental sensitivity as a function of increased oxidation state and coherence lifetime of the central transition in the only three available ions bearing the 4f^7^ valence configuration.

## Results

### Synthesis and Structure

The analytes [(Et_2_O)­K]­[Gd­(NP*)_4_] (**2-Gd** ^**3+**^), [(2.2.2-crypt)­K]­[Gd­(NP*)_4_] (**3-Gd** ^**3+**^) and [Tb­(NP*)_4_] (**4-Tb** ^**4+**^) (Figure 2) were synthesized as previously described while [K_2_Eu­(NP*)_4_] (**1-Eu** ^**2+**^) was synthesized through a modification of the procedure for [(THF)­K]­[(Et_2_O)­K]­[Eu­(NP*)_4_].^14,34^ The deviation from ideal *T*_d_ symmetry for the first coordination sphere of these complexes was quantified using the Σ_109.5_ metric, which reports the sum of deviations of the six angles of a four-coordinate complex from the ideal 109.5° tetrahedral angle.^52^ Complexes **2-Gd** ^**3+**^, **3-Gd** ^**3+**^, and **4-Tb** ^**4+**^ have Σ_109.5_ values of 28.4, 6.0, and 9.8° respectively,^34^ and **1-Eu** ^**2+**^ was found to have a value of 64.7°. Detailed structural comparisons of **1-Eu** ^**2+**^ and the previously reported [(THF)­K]­[(Et_2_O)­K]­[Eu­(NP*)_4_] can be found in the Supporting Information. Truncated molecular structures for the compounds investigated in this study are shown in Figure 1.

### Pulsed EPR Spectroscopy

Pulsed W-band (94 GHz) EPR measurements were performed using the high-power quasi-optical W-band spectrometer (HiPER) located at the National High Magnetic Field Laboratory (NHMFL) in Tallahassee, Florida^53,54^ on 2 mM frozen solutions of the analytes in either toluene (**1-Eu** ^**2+**^, **2-Gd** ^**3+**^, **4-Tb** ^**4+**^ **_tol_**) or 2-methyltetrahydrofuran (2-MeTHF) (**3-Gd** ^**3+**^, **4-Tb** ^**4+**^ **_THF_**). Further details regarding the spectrometer configuration and sample preparation can be found in the Supporting Information.

### Echo-Detected Field-Swept Spectra

Echo-detected field-swept (EDFS) spectra were collected for each sample at 5 K using a standard two-pulse Hahn echo sequence (π/2 – τ – π – τ – *echo*). Pulse lengths and wait times were varied to optimize echo intensity for each sample. Spectra were fit in *EasySpin* ^55^ using the effective spin-7/2 Hamiltonian

1 $${Ĥ}_{spin}=g_{iso}\mu_{B}\mathrm{B⃗}\cdot Ŝ+D\left\{{{Ŝ}_{z}}^{2}-\frac{1}{3}S\left(S+1\right)\right\}+E\left({{Ŝ}_{x}}^{2}-{{Ŝ}_{y}}^{2}\right)$$

where *g*_iso_ is the isotropic *g*-value, μ_*B*_ the Bohr magneton, ***B⃗*** the magnetic field vector, ***Ŝ*** the electronic spin operator, *S* the total spin of the system (*S* = 7/2), *Ŝ*_*i*_ (*i* = *x*,*y*,*z*) the components of the electronic spin operator along the *x*, *y*, and *z* directions respectively, and *D* and *E* the axial and rhombic components of the ZFS interaction. Results of the fits are given in Table 1, and the experimental spectra and fine-point simulations for **3-Gd** ^**3+**^ and **4-Tb** ^**4+**^ **_THF_** are plotted in Figure 3.

**Figure 3:**
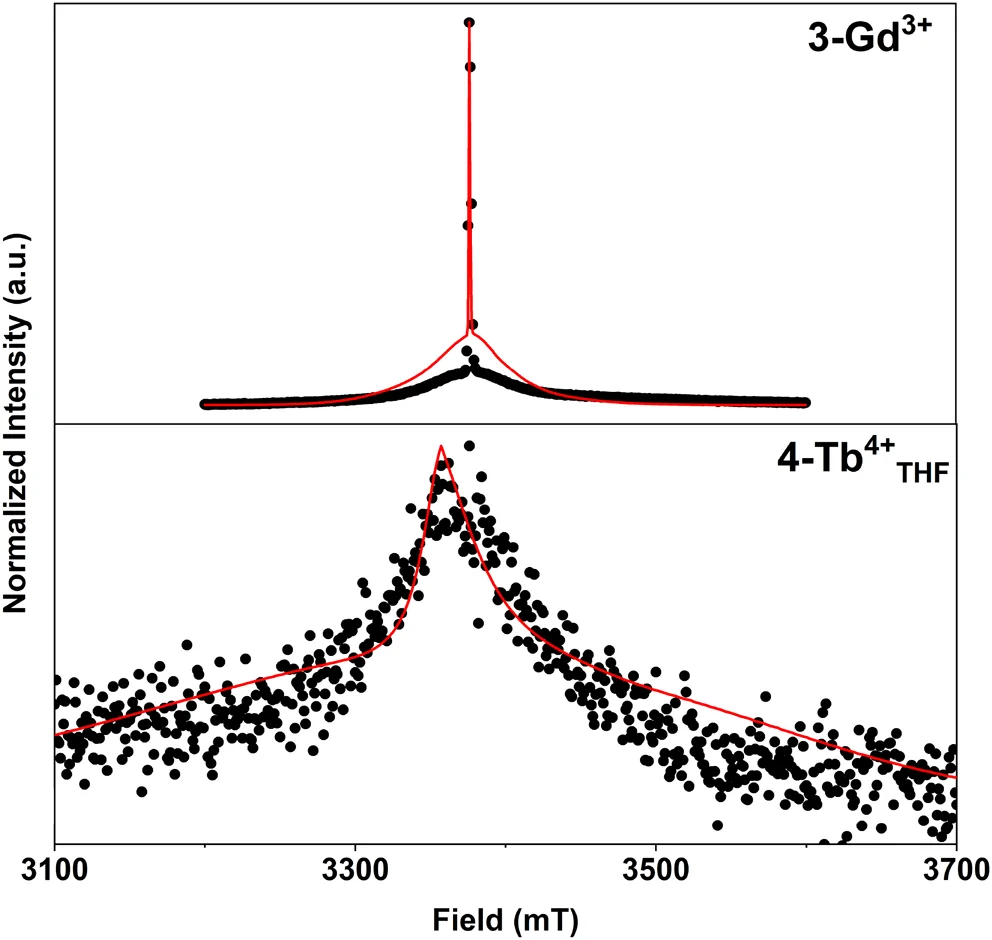
Co-plot of EDFS data (black) and fine-point simulations (red) for **3-Gd** ^**3+**^ and **4-Tb** ^**4+**^ **_THF_** in the range 3100–3700 mT at 5 K.

**Table 1:** Spin Hamiltonian Fit Parameters and Δ^8^S Values

Sample	*g*_*iso*_	*D* (cm^–1^)	*E* (cm^–1^)	Δ^8^ *S* (cm^–1^)
**1-Eu** ^**2+**^	1.9872(3)	–0.05043(13)	–0.0135(2)	0.696
**2-Gd** ^**3+**^	1.9878(6)	0.0807(2)	0.02178(14)	1.115
**3-Gd** ^**3+**^	1.9891(6)	0.0123(6)	0.0041(6)	0.179
**4-Tb** ^**4+**^ **_tol_**	2.0021(6)	0.1232(5)	0.0362(4)	1.733
**4-Tb** ^**4+**^ **_THF_**	2.0005(8)	0.1160(9)	0.0383(5)	1.676

Anisotropic line broadening was introduced as strain in the ZFS parameters (σ_*D*_ and σ_*E*_), with both parameters fixed to their reported values^34^ (those of [(THF)­K]­[(Et_2_O)­K]­[Eu­(NP*)_4_] used for **1-Eu** ^**2+**^) and modeled using explicit averaging. To minimize computational resources needed for fitting, the spectra were initially fit using a coarse sampling of the strain distributions (Figures S7–S11) and final simulations using the stable fit parameters were performed with finer samples to minimize truncation artifacts (Figures S12–S16, see Supporting Information for full fitting details). The energetic separation between the highest and lowest *m*_*s*_ doublets of the ground ^8^S state in absence of an applied field, denoted Δ^8^S,^34^ is calculated as the range of the eigenvalues of the ZFS operator and is given for each sample in Table 1.

#### *T*_1_ and *T*_m_ Measurements

Variable-temperature *T*_1_ and *T*_m_ measurements were performed at the field of maximum echo intensity corresponding to the central *m*_*s*_ = −1/2 → *m*_*s*_ = 1/2 transition for each analyte. Standard inversion recovery (π – *t*_wait_ – π/2 – τ – π – τ – *echo*) and two-pulse Hahn echo (π/2 – τ – π – τ – *echo*) sequences were used to measure *T*_1_ and *T*_m_, respectively, at each temperature. Pulse parameters were optimized for each temperature and sample to maximize echo intensity. Standard monoexponential functions were used to extract *T*_1_ and *T*_m_ (see Supporting Information for full details). Each measurement was performed in at least duplicate, and the presented results are arithmetic averages of the relevant data points with errors calculated via Gaussian error propagation. Full results are presented in Tables S4 and S5 and low-temperature results for **3-Gd** ^**3+**^ and **4-Tb** ^**4+**^ **_THF_** are plotted in Figure 4 (See Figures S18–S22 for full results). Measurements were performed at increasing temperatures until echoes were sufficiently weak to preclude meaningful interpretation. Additional data points were collected at temperatures below 5 K for the **4-Tb** ^**4+**^ species to supplement the small number of data points acquired at higher temperatures for these samples. Log–log plots of 1/*T*_1_ vs temperature are given in Figures S23–S27, and fits of the observed linear regimes reveal temperature dependencies for 1/*T*_1_ ranging T^0.40^-T^2.01^ across all samples.

**Figure 4:**
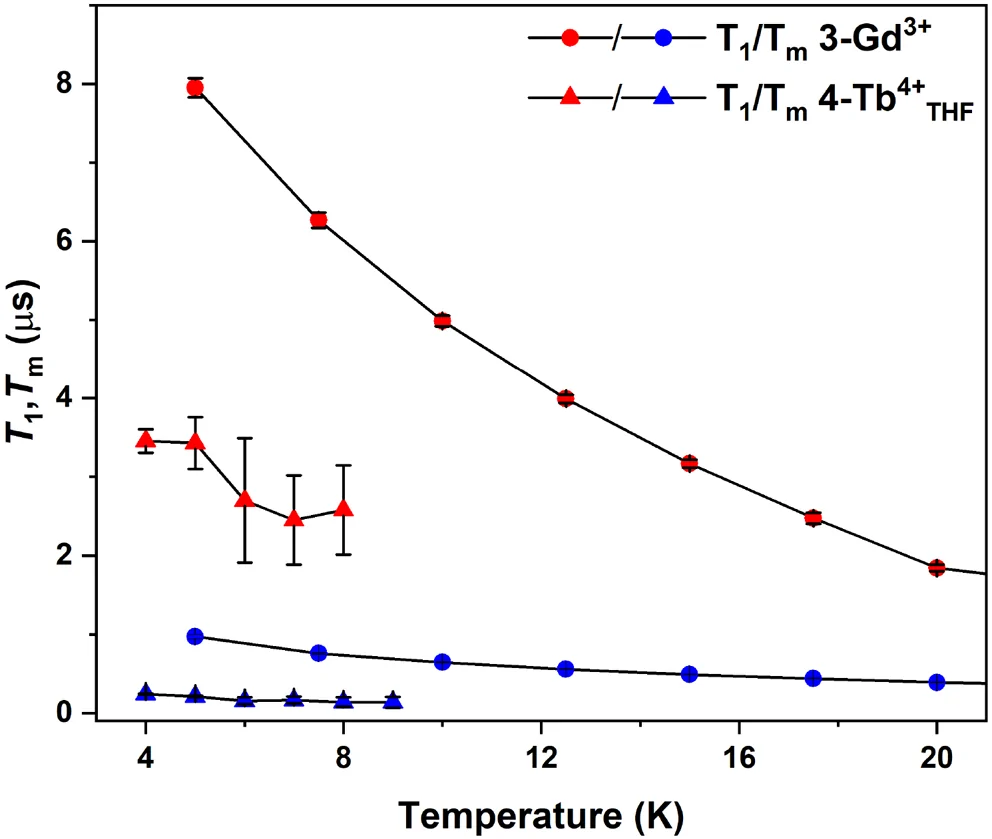
Co-plot of low-temperature *T*_1_ (red) and *T*_m_ (blue) vs temperature results for **3-Gd** ^**3+**^ (circles) and **4-Tb** ^**4+**^ **_THF_** (triangles). Lines between data points serve as visual guides.

#### *T*_m_ Fittings

Fitting of the *T*_m_ data for **1-Eu** ^**2+**^, **2-Gd** ^**3+**^, and **3-Gd** ^**3+**^ were performed using a modified version of the coherence lifetime model for the central transition proposed by Wilson et al.^56^

2 $$\frac{1}{T_{m}}=A_{1}\omega_{dd}\left(\mathrm{r̅}\right)\sum_{m,m\prime }W_{m,m\prime }n_{m}n_{m\prime }+\frac{F}{T_{1}}$$

where *A*_1_ and *F* weight the individual contributions, ω_dd_(r̅) is the dipole–dipole coupling strength for neighboring spin centers at an average distance *r̅*, *W*_*m*,*m*′_ is the overlap matrix element for an energy-conserving spin flip-flop between neighboring spin centers in arbitrary spin projection states *m* and *m*′ weighted by their respective Boltzmann populations *n*_*m*_ and *n*_*m*′_, and *T*_1_ is the spin–lattice relaxation time. Details on the full derivation of this modified model can be found in the Supporting Information. Fit results are summarized in Table 2 and full fit results are given in Figures S28–S30. The 5 K values for 1/*T*_m_ obtained experimentally and from the full fit in eq 2 are given in Table 2. The corresponding values obtained from extrapolating the high-temperature linear regime to 5 K (Figures S31–S33) are also provided.

**Table 2:** Results of Equation 2 Fits and Experimental, Full Fit, and Linear Extrapolation Values for 1/*T*_m_ at 5 K for **1-Eu** ^**2+**^, **2-Gd** ^**3+**^, and **3-Gd** ^**3+**^

Sample	*A*_1_(10^–3^)	*F*	1/*T*_m_, Experimental 5 K (μs^–1^)	1/*T*_m_, Full Fit 5 K (μs^–1^)	1/*T*_m_, Extrapolated 5 K (μs^–1^)
**1-Eu** ^**2+**^	5.0(8)	1.7(2)	1.433(11)	1.63	3.18
**2-Gd** ^**3+**^	1.2(6)	3.74(17)	1.117(15)	1.23	1.63
**3-Gd** ^**3+**^	2.7(4)	2.47(10)	1.03(3)	0.76	1.65

## Discussion

The analytes of interest in this study were synthesized as previously described,^14,34^ except in the case of **1-Eu** ^**2+**^, which necessitated a slight modification of the reported procedure to preclude coordinating solvents.^34^ The fit parameters for the EDFS spectra collected for each sample at 5 K are in reasonable agreement (as applicable) with those previously reported for solid-state and solution phase measurements.^34^ All spectra (Figures S7–S16) display broad features over much of the spectral width as a consequence of the large strain in ZFS parameters,^32^ which broadens many of the available transitions, as observed in the previous CW study.^34^ Additionally, a relatively sharp peak around *g* = 2 corresponding to the central transition is observed in all spectra.

To look for any trends in relaxation times across the series, variable-temperature *T*_1_ and *T*_m_ measurements were performed for each analyte. Qualitatively, a clear trend of increasing *T*_1_ and *T*_m_ for a given temperature appears upon moving from **1-Eu** ^**2+**^ to **2-Gd** ^**3+**^ to **3-Gd** ^**3+**^ (Figures S18–S22). This trend mirrors the increase in the excited ^6^P state energy moving from Eu^2+^ to Gd^3+^ and the decrease in distortion from *T*_d_ quantified by the decreasing Σ_109.5_ values (**1-Eu** ^**2+**^: 64.7°, **2-Gd** ^**3+**^: 28.4°, **3-Gd** ^**3+**^: 6.0°),^34^ with the increase from **2-Gd** ^**3+**^ to **3-Gd** ^**3+**^ accounted for by the decrease in distortion away from ideal *T*_d_ symmetry moving from an inner-sphere to an outer-sphere potassium cation. Based on this initial behavior, one might expect that the **4-Tb** ^**4+**^ species, with an even higher ^6^P energy and a similarly small distortion from *T*_d_ (Σ_109.5_: 9.8°)^34^ as **3-Gd** ^**3+**^, to follow this apparent trend. The opposite, however, is observed, with clear decreases from the Gd^3+^ species in both *T*_1_ and *T*_m_. In fact, **4-Tb** ^**4+**^ **_tol_** and **4-Tb** ^**4+**^ **_THF_** display the shortest *T*_m_ times across the entire series with exceptionally weak signal-to-noise ratios, even at 5 K, and large relative errors in *T*_1_ values. Decreasing relaxation times upon moving from Gd^3+^ to Tb^4+^ are not without precedent, with reports from both extended-solid and molecular species of greater thermal attenuation of CW EPR signals for Tb^4+^ systems indicative of shorter *T*_1_ times. To the best of our knowledge, no studies have elucidated the physical basis for this phenomenon.

To understand the mechanisms driving *T*_1_, the linear regimes of log–log plots of 1/*T*_1_ vs temperature were fit for each analyte (Figures S23–S27), revealing temperature dependencies for the spin–lattice relaxation rate spanning T^0.40^-T^2.01^. These results suggest that relaxation is primarily driven by direct processes in all compounds, with some higher order processes (such as Raman or Orbach) gaining prominence at higher temperature.^42,57–59^ The dominance of direct processes is not unexpected given the *B* ^4^ dependence of this mechanism^58^ and the high magnetic fields (∼3.35 T) at which the experiments were conducted. Given the *T*_1_ enhancements noted for isotropic systems,^5,10–12^ it is expected that *T*_1_ should scale inversely with increasing anisotropy (ZFS) holding other contributions constant. Despite this, no clear correlation is observed between the measured low temperature *T*_1_ (5 K) values and the total splitting of the ground ^8^S state at zero-field, denoted Δ^8^S (Figure S34), chosen to minimize ambiguities in comparing multiple ZFS terms of opposite sign. Confounding factors include differential coupling strengths to molecular vibrations, an active area of computational development.^48,60–64^

Considering *T*_m_ values, the coherence lifetime model proposed by Wilson et al. for the central transition provides an excellent starting point.^56^ This model explicitly considers three contributions to decoherence: (1) energy conserving nearest-neighbor electron spin flip-flops, (2) direct *T*_1_-driven decoherence, and (3) *T*_1_-induced relaxation on neighboring spins. At low temperature, *T*_1_ contributions should be minimal, allowing for examination of how electronic structure changes affect the contribution of the electron flip-flop mechanism, alongside other effects, such as nuclear spin diffusion and methyl tunnelling, that are not explicitly treated in this model.^42,56^

Interestingly, an inverse correlation is found between the 5 K *T*_m_ values and Δ^8^S for these compounds (Figure 5). While this correlation might be consistent with *T*_1_-driven decoherence (mechanisms 2 and 3 in the Wilson model), as ZFS induced anisotropy is expected to shorten *T*_1_, no correlation can be found between *T*_m_ and *T*_1_ at 5 K (Figure S37). To further examine the physical basis for this correlation, a modified form of Wilson’s model (eq 2) was employed, where mechanisms 2 and 3, which both depend on *T*_1_, are treated as a single component weighted by *F*. This simplification was necessitated by the lack of variable concentration data needed to differentiate contributions from the two mechanisms. Sufficient data were available for **1-Eu** ^**2+**^, **2-Gd** ^**3+**^, and **3-Gd** ^**3+**^ to apply this truncated model to these data sets and results are presented in Table 2 and Figures S28–S30. Attempts were made to apply this model to the **4-Tb** ^**4+**^ **_tol_** and **4-Tb** ^**4+**^ **_THF_** data sets, but the small number of data points combined with the large errors in *T*_1_ and *T*_m_ present in both precluded meaningful fits.

**Figure 5:**
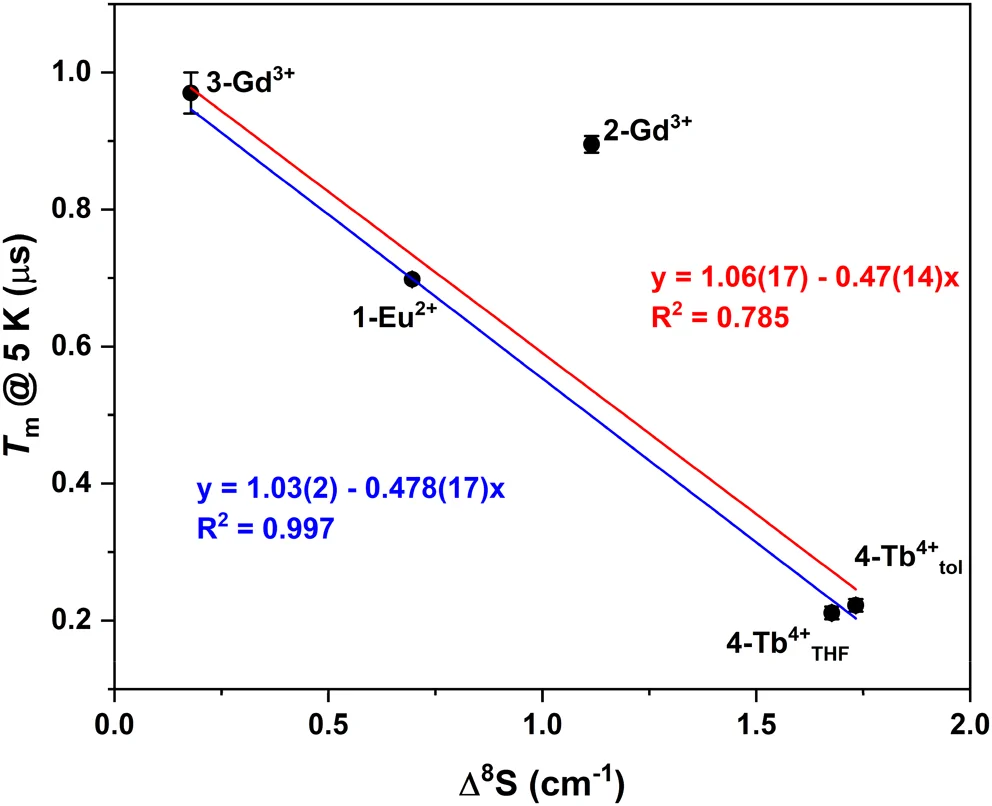
Plot of *T*_m_ values at 5 K vs Δ^8^S. Linear fits of the data are shown considering (red) and neglecting (blue) the **2-Gd** ^**3+**^ result.

Considering the individual fits, a clear difference between the results for **1-Eu** ^**2+**^ and **3-Gd** ^**3+**^ vs those of **2-Gd** ^**3+**^ can be seen at lower temperatures, where **1-Eu** ^**2+**^ and **3-Gd** ^**3+**^ demonstrate a deviation from linearity with decreasing temperature that is absent in **2-Gd** ^**3+**^ (Figure 6). The observed deviation from linearity with respect to 1/*T*_1_ in **1-Eu** ^**2+**^ and **3-Gd** ^**3+**^ is consistent with mechanism (1) from the Wilson model dominating relaxation at low temperatures, as anticipated, while the absence of this deviation in **2-Gd** ^**3+**^ is suggestive of *T*_1_ driven processes being more important at low temperatures in this system. This interpretation is consistent with the results of the fits to eq 2, which reveal a smaller *A*_1_ and larger *F* value for **2-Gd** ^**3+**^ than those found for the other compounds. Further evidence of the stronger influence of *T*_1_ contributions on the low temperature decoherence of **2-Gd** ^**3+**^ can be found through extrapolation of the high-temperature linear, *T*_1_-limited regimes of each data set to low temperature (Figures S31–S33). The resulting 1/*T*_m_ values predicted by the full fit and the extrapolation are presented in Table 2 alongside the experimental values.

**Figure 6:**
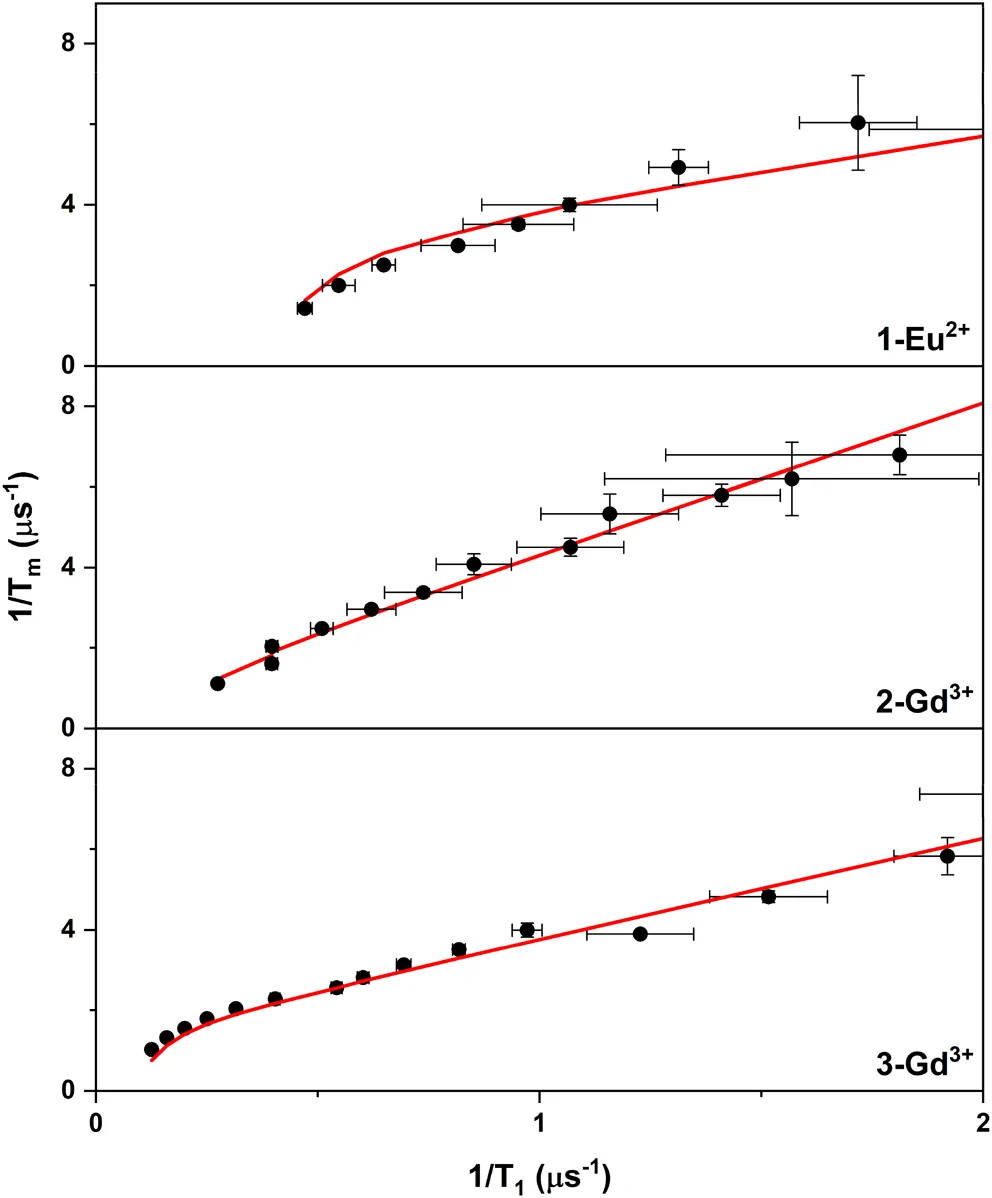
Low temperature plot of 1/*T*_m_ vs 1/*T*_1_ for **1-Eu** ^**2+**^ (top, ≤ 22.5 K) **2-Gd** ^**3+**^ (middle, ≤ 35 K), and **3-Gd** ^**3+**^ (bottom, ≤ 45 K) with associated errors. Fits of eq 2 to the data are shown in red.

Notably, the extrapolated 1/*T*_m_ values for **1-Eu** ^**2+**^ and **3-Gd** ^**3+**^ overestimate the decoherence rate roughly 2-fold as compared to the full fit values, while **2-Gd** ^**3+**^ demonstrates a greater agreement between the two values, with only a 33% overestimation from the extrapolation of the high-temperature behavior. A similar observation can be made comparing the extrapolated values to the experimental data points, though the overestimation in **3-Gd** ^**3+**^ in this comparison is somewhat attenuated with respect to the full fit values. This analysis supports the classification of **2-Gd** ^**3+**^, which deviates from the linear correlation between *T*_m_ at 5 K and Δ^8^S observed for the other compounds, as an outlier. The physical basis for this outlier behavior is not immediately apparent from this analysis, however the presence of a pendant diethyl ether, which has several noted low energy (∼100 cm^–1^) vibrational modes^65,66^ available for coupling to the metal center and is absent in the other complexes, may contribute to the extension of the *T*_1_-driven regime to lower temperatures in **2-Gd** ^**3+**^. Detailed low energy IR and/or Raman experiments coupled with theoretical calculations may shed further light on this behavior but are beyond the scope of this report.

Regardless, exclusion of the **2-Gd** ^**3+**^ data point from the linear fit then results in a near perfect inverse correlation between the two parameters, as evidenced by the near unity *R* ^2^ value (Figure 5). It is important to note that this empirical linear relationship should be interpreted with caution as, for values of Δ^8^S greater than 2.15 cm^–1^, this relationship cannot be valid as it predicts unphysically negative *T*_m_ values, suggesting additional parameters or higher-order functional forms are necessary to describe the relationship between Δ^8^S and *T*_m_ in these cases.

To better understand the source of this correlation, the Wilson model is examined in more detail. From the original model, the *A*_1_ term, which represents the contribution of the energy-conserving spin flip-flop mechanism to decoherence, is expected to scale inversely with the EPR line width. Wilson et al. proxy the line width with the degree of ZFS,^56^ which applied to the compounds in this study, would imply a larger *A*_1_ term for **1-Eu** ^**2+**^ than **3-Gd** ^**3+**^, the opposite of the observed behavior. This highlights a potential deficiency in the model to accurately weight the disparate broadenings of the *m*_*s*_ sublevels by the ZFS,^56^ though the influence of additional extrinsic factors (i.e., disorder or unresolved hyperfine coupling) on the phenomenological line widths cannot be discounted. Additionally, other processes which may be critical to decoherence in these systems, such as nuclear spin flip-flops,^42^ are not explicitly accounted for in the original model, and likely contribute at low temperatures.

This abridged model still leaves unclear, however, why the ZFS, which classically describes only the anisotropy of the ion and thus should primarily drive changes in *T*_1_ behavior,^5,10–12^ should correlate with *T*_m_. While the ZFS in these systems is primarily driven by second-order spin–orbit coupling, it ultimately represents an aggregate of the electronic and structural perturbations present in this system that promote mixing of excited states into the ground state, which may in turn serve to shorten *T*_m_. The rapid relaxation of the **4-Tb** ^**4+**^ species, however, obfuscates detailed interpretation, necessitating further synthetic design to achieve a molecular Tb^4+^ species with sufficiently long *T*_1_ and *T*_m_ times at low temperature to enable Wilson analyses. Therefore, elucidation of the basis for this observed correlation will entail further studies of new, carefully designed 4f^7^ series, paired with appropriate theoretical modeling. Examination of the concentration-dependence of the decoherence behavior will be particularly illuminating, enabling fitting with the full Wilson model to determine the limiting dipole–dipole interaction distance between neighboring electron spin centers.^56^ Additionally, rigorously isostructural analytes across all three isoelectronic ions would enable disambiguation of the role of structural and electronic contributions to the ZFS, and thus the decoherence properties of these ions.

As previously alluded, studies of isotropic metal ions (including Gd^3+^) have clearly demonstrated the diminished sensitivity of *T*_1_ and *T*_m_ to vibrations due to a lack of orbital anisotropy.^5,10–12^ While the formally isotropic 4f^7^ configuration of the ions studied here should afford the same benefits in the absence of first-order orbital angular momentum, the observed trends in ZFS and 5 K *T*_m_ suggest that subtle changes in overall electronic structure within this series, as reported by the aggregate Δ^8^S term, drive enhanced decoherence as a function of chemical oxidation state, despite seemingly advantageous changes in individual perturbations.

## Conclusions

In this work, we present W-band pulsed EPR measurements on an isovalent series of Eu^2+^, Gd^3+^, and Tb^4+^ complexes. Though no clear correlation was observed between *T*_1_ at 5 K and the zero-field splitting of the ^8^S ground state (Δ^8^S), an inverse correlation between the *T*_m_ value at 5 K and Δ^8^S is observed. This result emphasizes how tuning of the electronic structure in formally isotropic ions via chemical oxidation can undermine quantum coherence and highlights the need for concentration-dependent studies and rigorously isostructural analytes to fully map these effects. Though limited to the only available molecular series of Eu^2+^, Gd^3+^, and Tb^4+^ ions, this work more broadly demonstrates the disparities in electronic structure and resulting properties between differing oxidation states in lanthanide ions. These results also highlight the necessity of rigorously cubic coordination environments to realize Tb^4+^ qubits through the quenching of ZFS perturbations. The observed sensitivity of coherence properties to perturbations additionally commends dynamic EPR studies to map the effects of bonding in 5f^7^ ions (Cm^3+^ and Bk^4+^) wherein ligand-5f orbital overlap further increases crystal field and covalency contributions.

## Experimental Section

Synthetic details for **1-Eu** ^**2+**^ are given here. General considerations, characterization details, including IR spectroscopy, DC magnetometry, and crystallography, and details on pulsed EPR spectroscopy measurement, data processing and fitting are provided in the SI.

### [K_2_Eu­(NP*)_4_] (**1-Eu** ^**2+**^)

This compound was synthesized following a modified literature procedure. In the glovebox, a 20 mL scintillation vial was charged with [EuI_2_(THF)_2_] (105.85 mg, 0.191 mmol), hexanes (2 mL), and a glass coated magnetic stirring bar. To this mixture was added a solution of [K­(NP­(CH_2_N*^t^* Bu)_2_(Et_2_N))] (249.78 mg, 0.765 mmol, 4.01 equiv) in hexanes (2 mL), and the resulting light green suspension was allowed to stir overnight at room temperature. The now bright orange reaction mixture was filtered through a pad of Celite on a fine porosity glass frit, and the tan solids were washed with minimal additional hexanes. The resulting bright orange filtrate was reduced *in vacuo* and triturated with pentane (3 × 2 mL) to afford a waxy orange residue. This residue was crystallized from evaporation of a concentrated solution in pentane into a reservoir of toluene at −35 °C to afford the product as XRD quality single crystals (187.06 mg, 71%). IR (cm^–1^): *v.* 3015 (vw), 2964 (w), 2902 (w), 2829 (w), 1463 (vw), 1384 (w), 1355 (w), 1305 (vw), 1262 (w), 1245 (w), 1196 (s), 1178 (s), 1143 (m), 1093 (vw), 1040 (m), 1021 (m), 966 (m), 917 (m), 863 (w), 789 (w), 679 (s), 608 (w), 517 (w), 492 (s), 465 (w), 447 (vw). Elem. Anal. Found (calc): C, 48.60 (48.75); H, 9.46 (9.35); N, 16.20 (16.24).

## Supplementary Material



## Notes

General considerations, IR and EPR spectroscopy, magnetometry, and crystallographic data (PDF)
