# Supplementary material for: Oxidation State Effects on Decoherence in Isovalent 4f7 Complexes
Source: Inorg Chem. 2026 Oct 8;65(42):25005–13. doi: 10.1021/acs.inorgchem.6c04187 (PMC13722037; doi:10.1021/acs.inorgchem.6c04187)
Supplement: Supplementary file 1 [file ic6c04187_si_001.pdf]

Supporting Information:

Oxidation State Effects on Decoherence in Isovalent 4f^7 Complexes

Grant R. Wilkinson,[a] Manoj V. H. Subramanya,[b] Maximilian G. Bernbeck,[a] Kavipriya Thangavel,[b] Stephen Hill,[b,c,d] and Henry S. La Pierre[a,e,f]*

[a] School of Chemistry and Biochemistry, Georgia Institute of Technology, Atlanta, GA 30332-0400, United States

[b]National High Magnetic Field Laboratory, Florida State University, Tallahassee, FL 32310, United States

[c]Department of Physics, Florida State University, Tallahassee, FL 32306, United States

[d]Department of Chemistry and Biochemistry, Florida State University, Tallahassee, FL 32306, United States

[e]Nuclear and Radiological Engineering Program and Health Physics Program, School of Mechanical Engineering, Georgia Institute of Technology, Atlanta, GA 30332-0400, United States

[f]Physical Sciences Division, Pacific Northwest National Laboratory, Richland, WA 99352, United States

* *hsl@gatech.edu*

Contents

General Considerations S2

IR Spectroscopy S3

Crystallographic Information S4

DC Magnetometry S6

EPR Spectroscopy S8

Sample Preparation S8

EDFS Measurements and Fitting S8

T_1 and T_m Measurements S17

T_1 Power Law Fits S22

T_m Mechanistic Fits S24

Trends in T_1 and T_m at 5 K S28

References S31

General Considerations

Unless otherwise noted, all reagents were obtained from commercial suppliers and used without further purification unless otherwise noted. The syntheses and manipulations described herein were conducted under argon or nitrogen with exclusion of oxygen or water using standard Schlenk techniques or in an inert atmosphere glovebox (Vigor) under a dinitrogen (<0.1 ppm O_2/H_2O) atmosphere unless otherwise noted. The glovebox is equipped with a -35 ºC freezer and a cold well. All glassware and cannulae were stored in an oven overnight (>8 h) at a temperature of ca. 160 ºC. Celite and molecular sieves were dried under vacuum at a temperature > 250 ºC for a minimum of 24 h. Toluene, pentane, and hexanes were sparged with UHP-grade argon (Airgas) and passed through columns containing Q-5 and molecular sieves in a solvent purification system (JC Meyer Solvent Solutions). All solvents in the glovebox were stored in bottles over 3 Å molecular sieves. Anhydrous 2-methyltetrahydrofuran (2-MeTHF, unstabilized, Acros) was sparged with UHP-grade argon (Airgas) and stored over 3 Å molecular sieves in the glovebox prior to use. $[K(NP(CH_2N^tBu)_2(Et_2N))]$ (KNP*),[1] $[(Et_2O)K][Gd(NP^*)_4]$ (**2-Gd^{3+}**),[2] $[(2.2.2\text{-crypt})K][Gd(NP^*)_4]$ (**3-Gd^{3+}**),[2] $[Tb(NP^*)_4]$ (**4-Tb^{4+}**),[1] and $[EuI_2(THF)_2]$[3] were prepared according to literature procedures. Cargile Type NVH immersion oil was degassed on a Schlenk line by stirring under active vacuum with gentle heating (to facilitate stirring, ca. 40 ºC) overnight prior to use. Infrared (IR) spectra were recorded on a Bruker ALPHA FTIR spectrometer from 400 to 4000 cm^{-1} using attenuated total reflection (ATR) and peaks are listed in wavenumber [cm^{-1}] and intensity is indicated by the following abbreviations: vw (very weak); w (weak); m (medium); s (strong); vs (very strong); br (broad). Elemental analysis (CHN) was performed at the University of Iowa MATFab Facility.

Crystals suitable for single-crystal x-ray diffraction were immersed in NVH oil and transferred to the diffractometer in sealed 20 mL scintillation vials. The data for the structure reported in this paper was collected from a shock-cooled single crystal at 100 K on a Bruker D8 VENTURE dual wavelength Mo/Cu three-circle diffractometer with a microfocus sealed x-ray tube using a mirror optics as monochromator and a Bruker PHOTON III detector. The diffractometer was equipped with an Oxford Cryostream 800 low-temperature device and used Mo-K_α radiation (λ = 0.71073 Å) for the reported collection. Information on structural solutions and refinement details are described in the Crystallography section. Crystallographic data for the structure reported in this paper has been deposited with the Cambridge Crystallographic Data Centre.[4] CCDC 2473801 contains the supplementary crystallographic data for this paper. This data can be obtained free of charge from the Cambridge Crystallographic Data Centre via www.ccdc.cam.ac.uk/structures. The crystallographic report and CIF file were generated using FinalCIF.[5] Structures were visualized and analyzed in Mercury (CCDC) and graphics were generated using POV-Ray.[6,7]

Quartz wool was used to immobilize samples for DC magnetometry measurements and was cleaned following a modification of a published procedure.[8] Quartz wool (Technical Glass) was leached with a mixture of oxalic acid (0.1 M) and sulfuric acid (0.5 M) in deionized water in a covered glass beaker for 3 h at 90 ºC with occasional agitation using a glass stirring rod. The quartz wool was then collected on a glass frit, washed with deionized water (1 L) and dried *in vacuo* overnight (>8 h). The wool was further dried in an oven set to 160 ºC overnight (>8 h) prior to being brought in the glovebox for use.

IR Spectroscopy

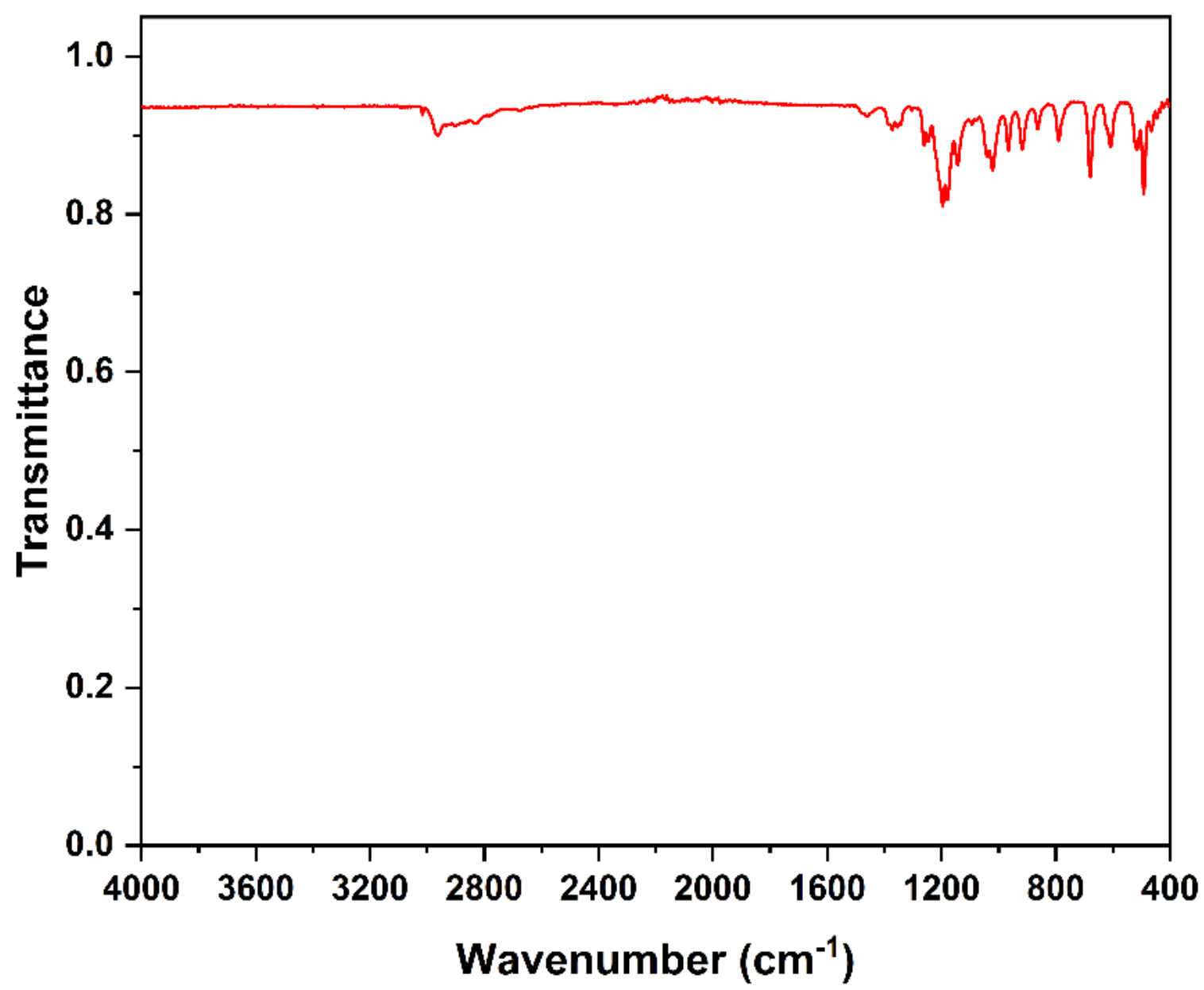

Figure S1. IR spectrum of **1-Eu^{2+}**.

Crystallographic Information

Table S1. Crystallographic data for **1-Eu^{2+}**.

Compound	***1-Eu^{2+}***
Empirical formula	$C_{63}H_{136}EuK_2N_{16}P_4$
Formula weight	1471.91
Temperature [K]	100.00
Crystal system	monoclinic
Space group (No.)	$P2_1/n$ (14)
a [Å]	15.003(4)
b [Å]	22.192(7)
c [Å]	23.990(8)
α [°]	90
β [°]	98.713(12)
γ [°]	90
Volume [Å^3]	7895(4)
Z	4
ρ_{calc} [gcm^{-3}]	1.238
μ [mm^{-1}]	1.027
F(000)	3148
Crystal size [mm^3]	0.317 × 0.24 × 0.138
Crystal color/habit	Clear orange plank
Radiation	Mo-K_α (λ = 0.71073 Å)
2θ range [°]	5.04 to 60.90 (0.70 Å)
Index ranges	$-21 \le h \le 21$
	$-31 \le k \le 31$
	$-32 \le l \le 34$
Reflections collected	551271
Independent reflections	23806
	R_{int} = 0.0907
	R_{sigma} = 0.0287
Completeness to θ = 25.242°	99.2 %
Data / Restraints / Parameters	23806/42/1131
Goodness-of-fit on F^2	1.126
Final R indexes	R_1 = 0.0261
[I≥2σ(I)]	wR_2 = 0.0598
Final R indexes	R_1 = 0.0351
[all data]	wR_2 = 0.0675
Largest peak/hole [eÅ^{-3}]	0.82/-0.60

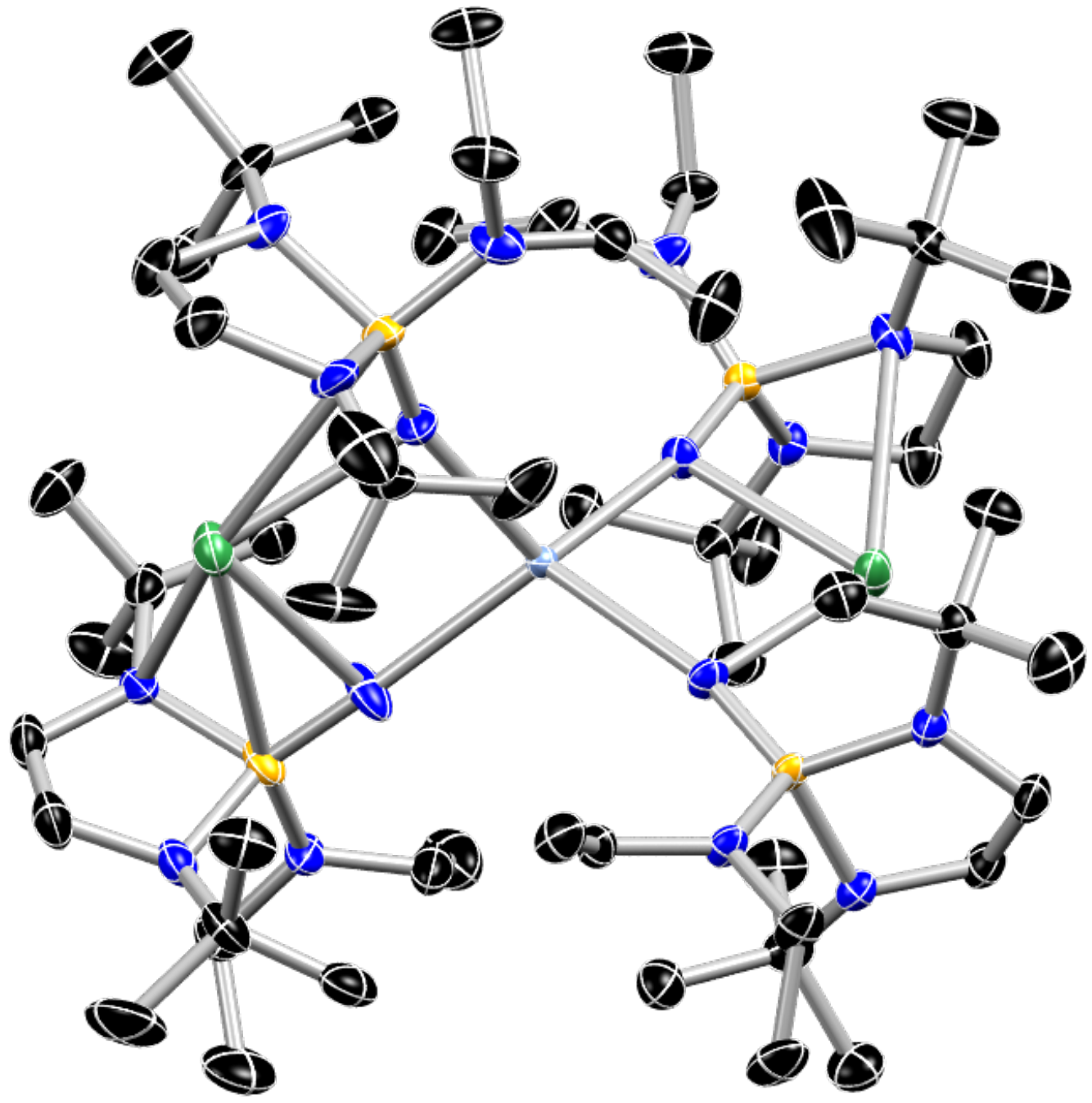

Figure S2. Molecular structure of **1-Eu^{2+}** with thermal ellipsoids set at 50 % probability. Black, blue, orange, green, and blue-grey represent C, N, P, K, and Eu respectively. H atoms, disordered components and solvent molecules are omitted for clarity.

Solution and Refinement Details for 1-Eu^{2+}. All data were integrated with SAINT and a multi-scan absorption correction using SADABS was applied.[9, 10] The structure was solved using direct methods using SHELXT and refined by full-matrix least-squares methods against F^2 by SHELXL using Olex2.[11-13] All non-hydrogen atoms were refined with anisotropic displacement parameters. All C-bound hydrogen atoms were refined isotropic on calculated positions using a riding model with their U_{iso} values constrained to 1.5 times the U_{eq} of their pivot atoms for terminal sp^3 carbon atoms and 1.2 times for all other carbon atoms. Disordered components in ethyl and tert-butyl residues and the solvent toluene molecule were modelled with the SPLIT SAME command, applying a SADI restraint to correlated bond lengths and allowing the two components to refine their occupancies to a total occupancy of 1. Each disordered residue was modelled with a separate free variable. No further restraints or constraints were applied.

Structural Comparison of 1-Eu^{2+} and [(THF)K][(Et$_2$O)K][Eu(NP*)$_4$]. 1-Eu^{2+} features slightly elongated Eu-N (2.490(4) vs 2.474(8) Å) and P-N$_{imide}$ (1.531(4) vs 1.519(8) Å) bond lengths in comparison to [(THF)K][(Et$_2$O)K][Eu(NP*)$_4$],[2] which likely arise from the increased Lewis acidity of the non-coordinated potassium cations in **1-Eu^{2+}**. The deviation in the geometry of the first coordination sphere in **1-Eu^{2+}** can be quantified using the $\Sigma_{109.5}$ metric, which measures the sum of the deviations from the six unique angles in a four-coordinate complex from the ideal tetrahedral angle 109.5° as part of a larger equation to quantify the pyramidalization of nitrogen-coordinated boranes.[14] For **1-Eu^{2+}** this metric yields a value of 64.7°, representing only a minor further distortion away from [(THF)K][(Et$_2$O)K][Eu(NP*)$_4$] with its corresponding value of 60.6°.[2] Overall, no significant structural changes are noted for **1-Eu^{2+}** as compared to the previously reported [(THF)K][(Et$_2$O)K][Eu(NP*)$_4$].

DC Magnetometry

Magnetic measurements were performed on a Quantum Design MPMS3 magnetometer. Samples were prepared inside a glovebox by adding a measured amount of sample to a half-sealed quartz tube using a plastic spatula to transfer and funnel sample into the tube. A measured amount of quartz wool was then added to the tube and compressed onto the sample using a PTFE rod. The unsealed top of the tube was attached to a homemade vacuum assembly (*vide infra*, parts list available upon request), pulled under static vacuum, and the tube was sealed using an H_2/O_2 torch. The sealed tube was then pressure-fitted into a plastic straw using PTFE tape and loaded onto the instrument. Variable temperature and field, zero field cooled (ZFC) measurements were performed as previously described.[15]

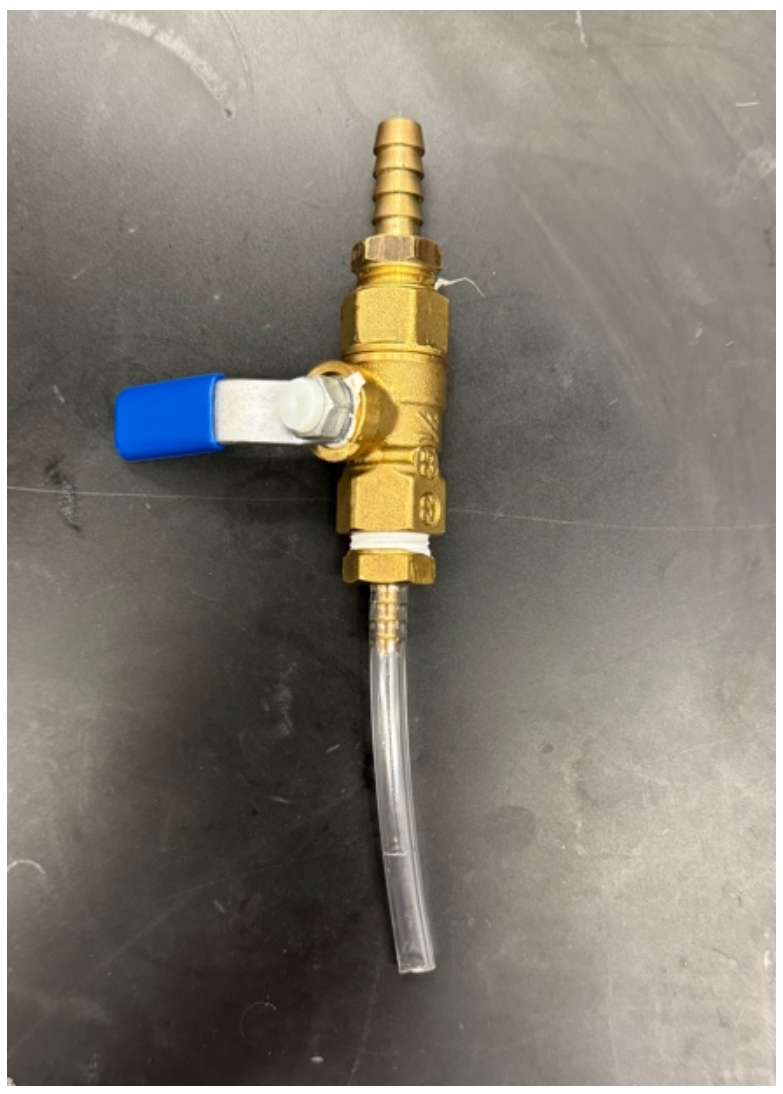

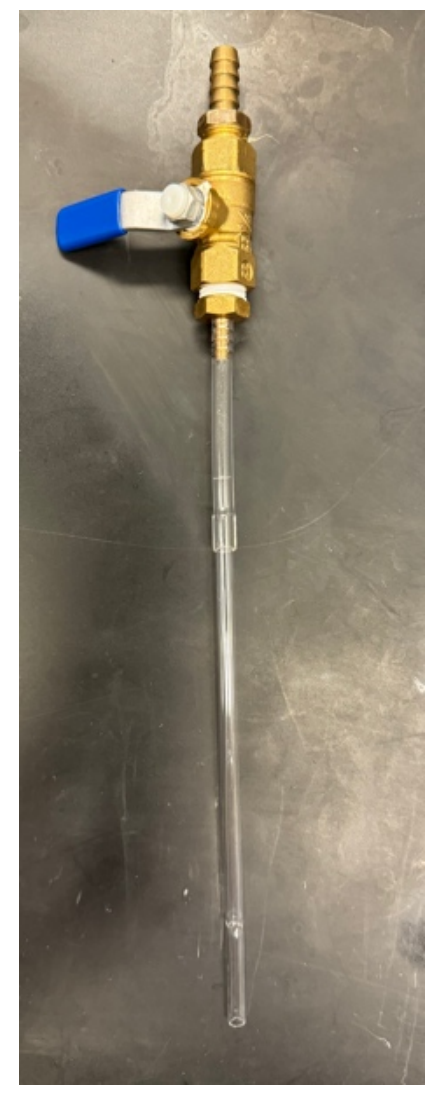

Figure S3. (Left) Homemade vacuum assembly for SQUID sample sealing and (right) example usage of vacuum assembly with empty quartz SQUID tube.

Diamagnetic corrections for **1-Eu^{2+}** and quartz wool were performed using Pascal's constants[16] (tabulated in Table S2). Contributions from the quartz sample tube and plastic straw are negligible compared to the sample and are not considered in this analysis.

Table S2. Molecular weights and diamagnetic corrections for **1-Eu^{2+}** and quartz wool.

Compound	*Molecular Weight (g/mol)*	*Diamagnetic Correction (10^{-6} emu/mol)*
1-Eu^{2+}	1379.77	-951.4
Quartz Wool (SiO_2)	60.08	-2.5

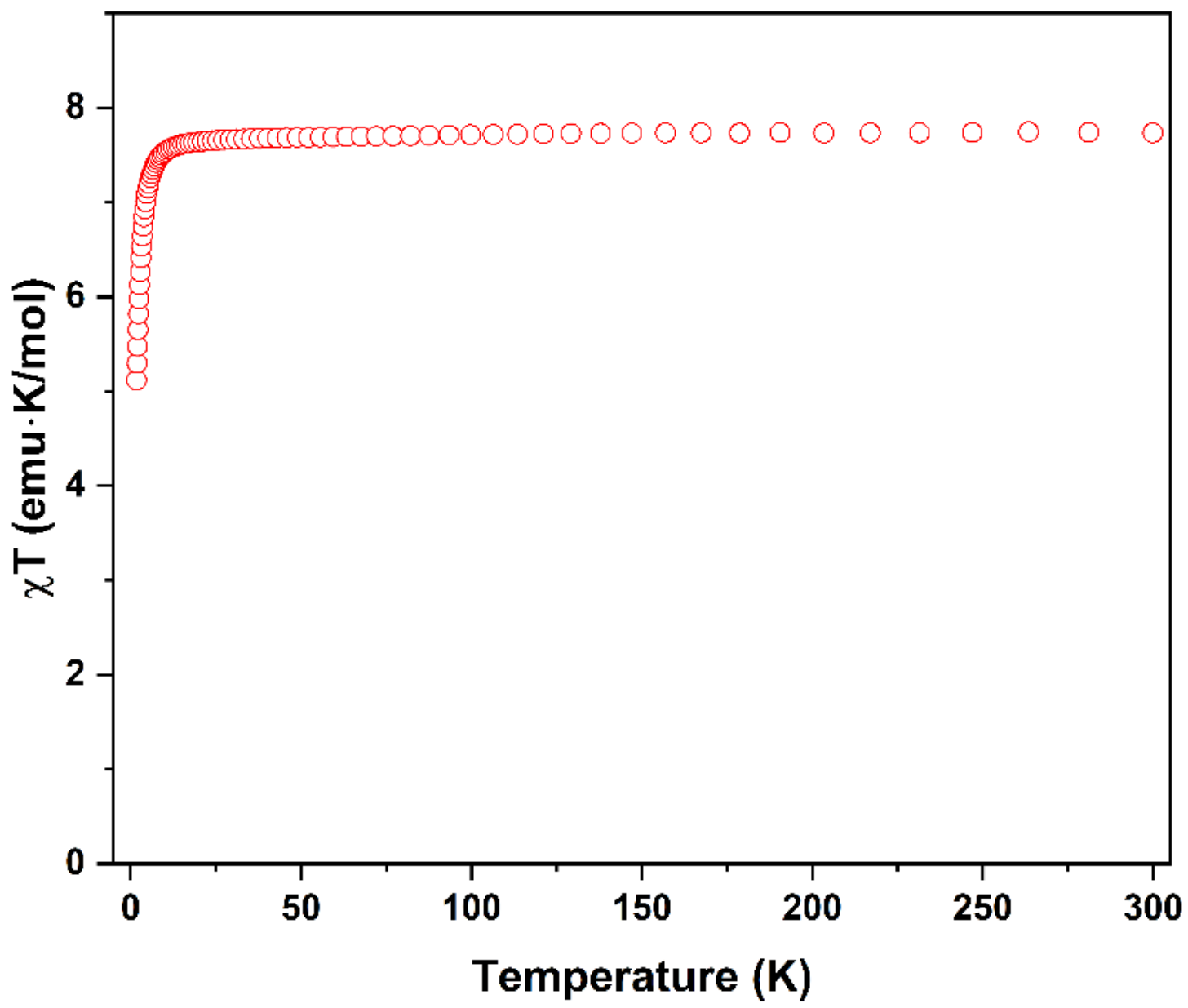

Figure S4. χT vs. T for **1-Eu^{2+}** measured at 1.0 T zero-field cooled from 1.8-300 K. The room temperature moment of 7.73 emu·K/mol agrees well with the expected moment of 7.88 emu·K/mol for an isotropic 4f^7 ion.[17]

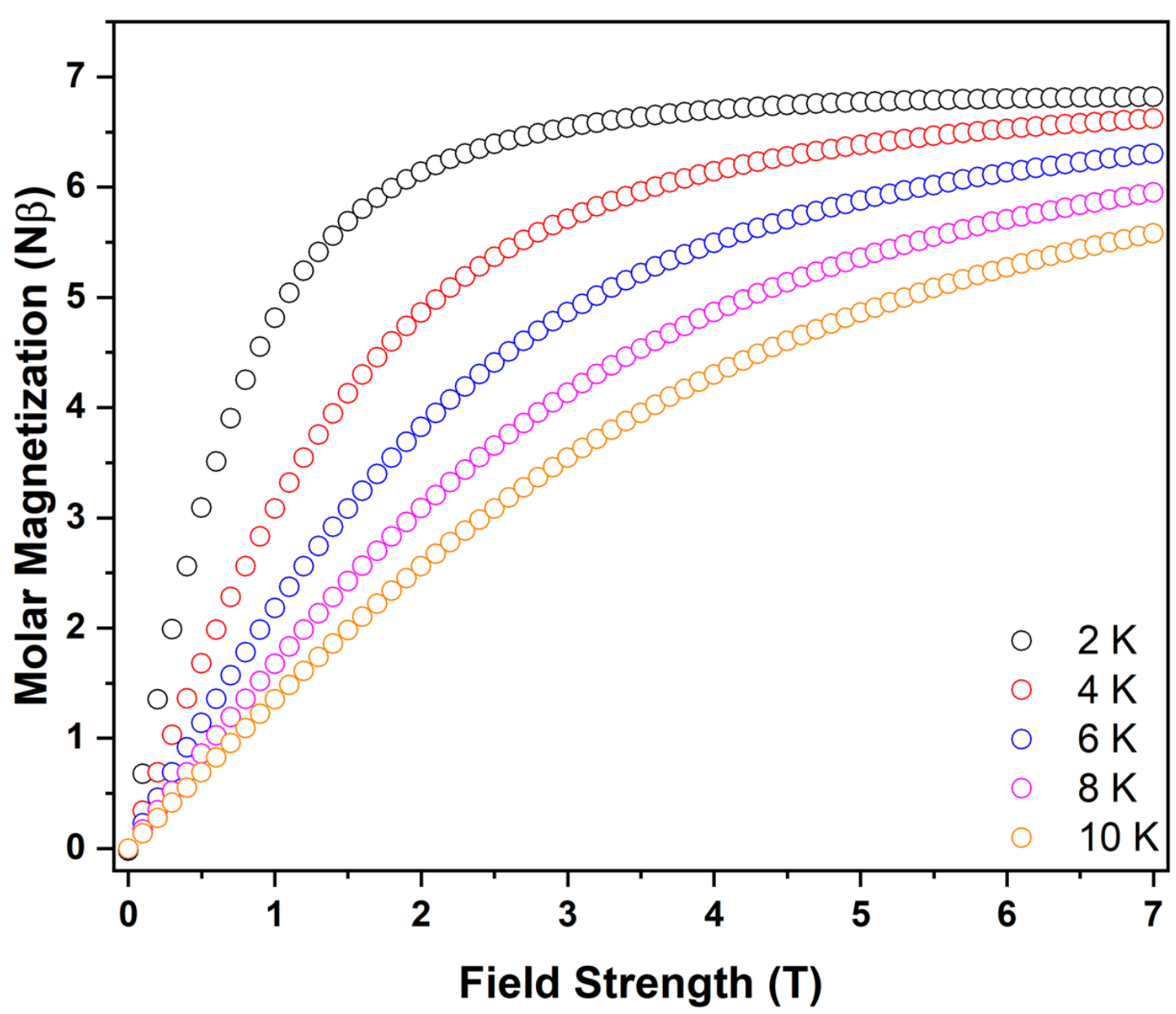

Figure S5. Molar magnetization for **1-Eu^{2+}** under an applied field of 0-7 T measured at 2, 4, 6, 8, and 10 K. The 2 K saturation value of 6.82 Nβ is consistent with the expected value of 7 Nβ for an isotropic 4f^7 ion.[17]

EPR Spectroscopy

All pulsed measurements were performed at 94 GHz using the high-power quasi-optical W-band spectrometer (HiPER) located at the National High Magnetic Field Laboratory (NHMFL) in Tallahassee, Florida.[18, 19] In this study, we employed a 10 W solid-state amplifier (QuinStar Technologies, Torrence, CA, USA) in place of a high-power extended interaction klystron (EIK) amplifier. An advantage of the solid-state amplifier is the absence of duty cycle limitations. Data was acquired using the specman4EPR program.[20]

Sample Preparation. Pulsed EPR measurements were performed on 2 mM solutions of the compounds in either toluene or 2-MeTHF glass. Solutions were initially prepared inside of the glovebox and loaded inside PTFE sample holders, which were then capped with a small plug of PTFE wire followed by a small amount of Apiezon® H grease (Figure S6).

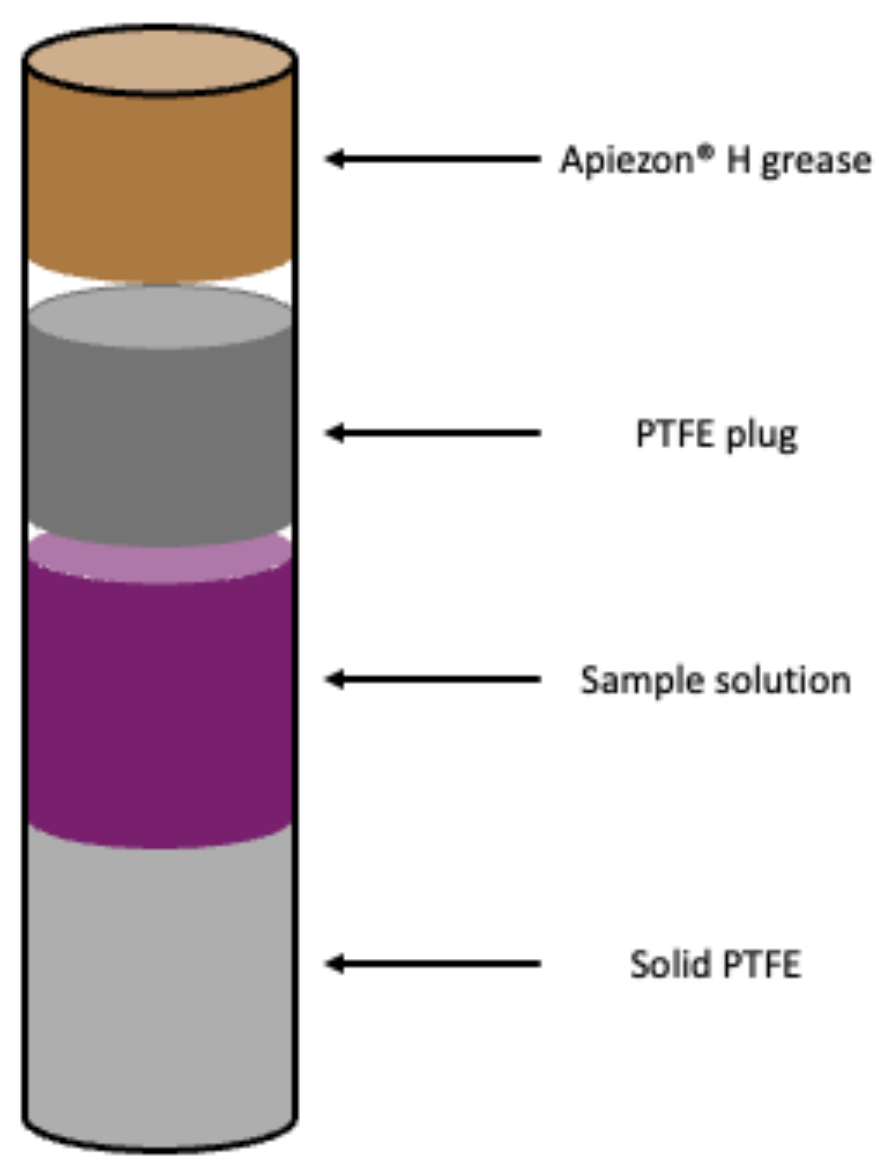

Figure S6. Diagram of prepared sample for frozen solution measurements.

Prepared samples were then placed inside 4 mL shell vials and allowed to freeze inside of the glovebox cold well before being heat-sealed in Mylar bags, removed from the glovebox, and shipped to the instrument frozen. At the instrument, samples were removed from the Mylar bags and immediately immersed in liquid nitrogen to maintain sample integrity and preclude introduction of water or oxygen to the samples. Under liquid nitrogen, the samples were carefully loaded into pre-chilled sample pucks and then quickly loaded onto the instrument and cooled to 5 K.

The analyte **4-Tb^{4+}** was measured in both toluene and 2-MeTHF to facilitate comparisons and the datasets are denoted as **4-Tb$^{4+}_{tol}$** and **4-Tb$^{4+}_{THF}$** respectively.

EDFS Measurements and Fitting. Echo-detected field swept (EDFS) spectra were recorded at 5 K using a standard Hahn echo sequence ($\frac{\pi}{2} - \tau - \pi - \tau - echo$) for each sample and the magnitude of the resulting

signal (real and imaginary components) were calculated and normalized to yield the presented spectra. Pulse parameters were optimized for each sample and typical values for $\frac{\pi}{2}$ and τ ranged from 40-150 ns and 280-600 ns respectively. These spectra were fit using EasySpin 6.0.9[21] as implemented in the MatLab® (MathWorks) toolbox. Least-squares fittings were performed using the *esfit* GUI function and the *pepper* simulation function utilizing the Nelder-Mead simplex algorithm with data "as is" as target, "maxabs" scaling, and no baseline for **1-Eu^{2+}**, **2-Gd^{3+}**, and **3-Gd^{3+}** (an offset baseline was necessary to adequately fit the spectra of **4-Tb^{4+}** due to the overall noisiness of the data). Spectra for the analytes were fit as S = 7/2 systems using the spin Hamiltonian:

$$\hat{H}_{spin} = g_{iso}\mu_B\vec{\boldsymbol{B}}\cdot\hat{\boldsymbol{S}} + D\left\{\hat{S}_z{}^2 - \frac{1}{3}S(S+1)\right\} + E\left(\hat{S}_x{}^2 - \hat{S}_y{}^2\right) \quad \text{(S1)}$$

Where g_{iso} is the isotropic g-value, μ_B the Bohr magneton, $\vec{\boldsymbol{B}}$ the magnetic field vector, $\hat{\boldsymbol{S}}$ the electronic spin operator, S the total spin of the system, $\hat{S}_i(i = x, y, z)$ the components of the electronic spin operator along the x, y, and z directions respectively, and D and E are the axial and rhombic components of the zero-field splitting (ZFS) interaction. Isotropic Lorentzian line broadening was applied to each analyte spectra using the *lwpp* keyword and was fixed at 1 mT for **1-Eu^{2+}**, **2-Gd^{3+}**, and **4-Tb^{4+}**, and at 0.1 mT for **3-Gd^{3+}**. As previously described,[2] the modelling of these systems requires the inclusion of anisotropic line broadening in the form of *D* and *E* strain (σ_D and σ_E respectively). To simplify the fitting procedure, these values were fixed at the reported values for **2-Gd^{3+}**, **3-Gd^{3+}**, and **4-Tb^{4+}** (the reported parameters for [(THF)K][(Et$_2$O)K][Eu(NP*)$_4$] were used for **1-Eu^{2+}**).[2] Due to the relatively large values of the strain parameters compared to the central values (>10 %), explicit strain averaging was performed following the EasySpin documentation and averaging over 11 points in the Gaussian distributions for *D* and *E* defined by the central value and the fixed strain (defining the full-width at half-maximum). The code utilized to fit **2-Gd^{3+}** via this methodology is given below:

```
%Define the spin system with g and ZFS values initialized to reported
%values
Sys.S = 7/2;
Sys.g = 1.990;
Sys.D = [0.086 0.018]*29979.2458; %[D E] converted to MHz

Sys.lwpp = [0 1]; %two component broadening with [Gauss Lorentz]

Exp.mwFreq = 94;
Exp.Range = [2500, 4400];
Exp.nPoints = 1901;
Exp.Temperature = 5;
Exp.Harmonic = 0;

%Initial variance range for esfit GUI, set to 3x reported ESD
SysVary.g = 0.015;
SysVary.D = [0.009 0.009]*29979.2458;
SysVary.lwpp = [10 10]; %constrained in GUI

function spec = Averaging(Sys,Exp) %nest explicit averaging components from EasySpin
documentation to allow least-squares fitting
    Sys0.S = Sys.S; %import values defined outside function
```

```
    Sys0.g = Sys.g;
    Sys0.lwpp = Sys.lwpp;

    %fixed to literature values
    Dfwhm = 0.017*29979.2458; %DStrain converted to MHz
    Efwhm = 0.017*29979.2458; %EStrain converted to MHz

    %create distribution around central value and select 11 points for
    %averaging
    D = linspace(0,2*Sys.D(1),11);       % range of D values, MHz
    Dweights = gaussian(D,Sys.D(1),Dfwhm); % associated weights
    E = linspace(0,2*Sys.D(2),11);       % range of E values, MHz
    Eweights = gaussian(E,Sys.D(2),Efwhm); % associated weights

    spec = 0;
    for iD = 1:numel(D) %loop through D values in distribution
        Sys0.D(1) = D(iD);
        for iE = 1:numel(E) %loop through E values in distribution
            Sys0.D(2) = E(iE);
            [B,spec_] = pepper(Sys0,Exp); %simulate spectrum with particular D and E
within distribution
            spec = spec + Dweights(iD)*spec_*Eweights(iE); %add spectrum scaled to
weight of D and E values in respective distributions
        end
    end
end

esfit(spc,@Averaging,{Sys,Exp},{SysVary}); %simulate using esfit for the explicit
averaging function
```

The minimization landscape for **4-Tb^{4+}$_{THF}$** was sufficiently shallow as to require sequential fitting of the *g* and ZFS values while constraining the other parameters to those determined from the **4-Tb^{4+}$_{tol}$** spectrum prior to allowing all parameters to relax for the final fit. Parameters from the fits of the analyte spectra are given in Table S3 and the experimental and fitted spectra are shown in Figures S7-S11.

Table S3. Simulation parameters for EDFS spectra of analytes and derived parameter $\Delta\ ^8S$.

Sample	g_{iso}	*D* (cm^{-1})	*E* (cm^{-1})	σ_D (cm^{-1})[a]	σ_E (cm^{-1})[a]	$\Delta\ ^8S$ (cm^{-1})[b]
1-Eu^{2+}	1.9872(3)	-0.05043(13)	-0.0135(2)	0.020	0.020	0.696
2-Gd^{3+}	1.9878(6)	0.0807(2)	0.02178(14)	0.017	0.017	1.115
3-Gd^{3+}	1.9891(6)	0.0123(6)	0.0041(6)	0.020	0.010	0.179
4-Tb^{4+}$_{tol}$	2.0021(6)	0.1232(5)	0.0362(4)	0.095	0.035	1.733
4-Tb^{4+}$_{THF}$	2.0005(8)	0.1160(9)	0.0383(5)	0.095	0.035	1.676

[a]Constrained during fitting procedure to the reported values from Gompa *et al.*[2]
[b]Calculated from eigenvalues of ZFS Hamiltonian

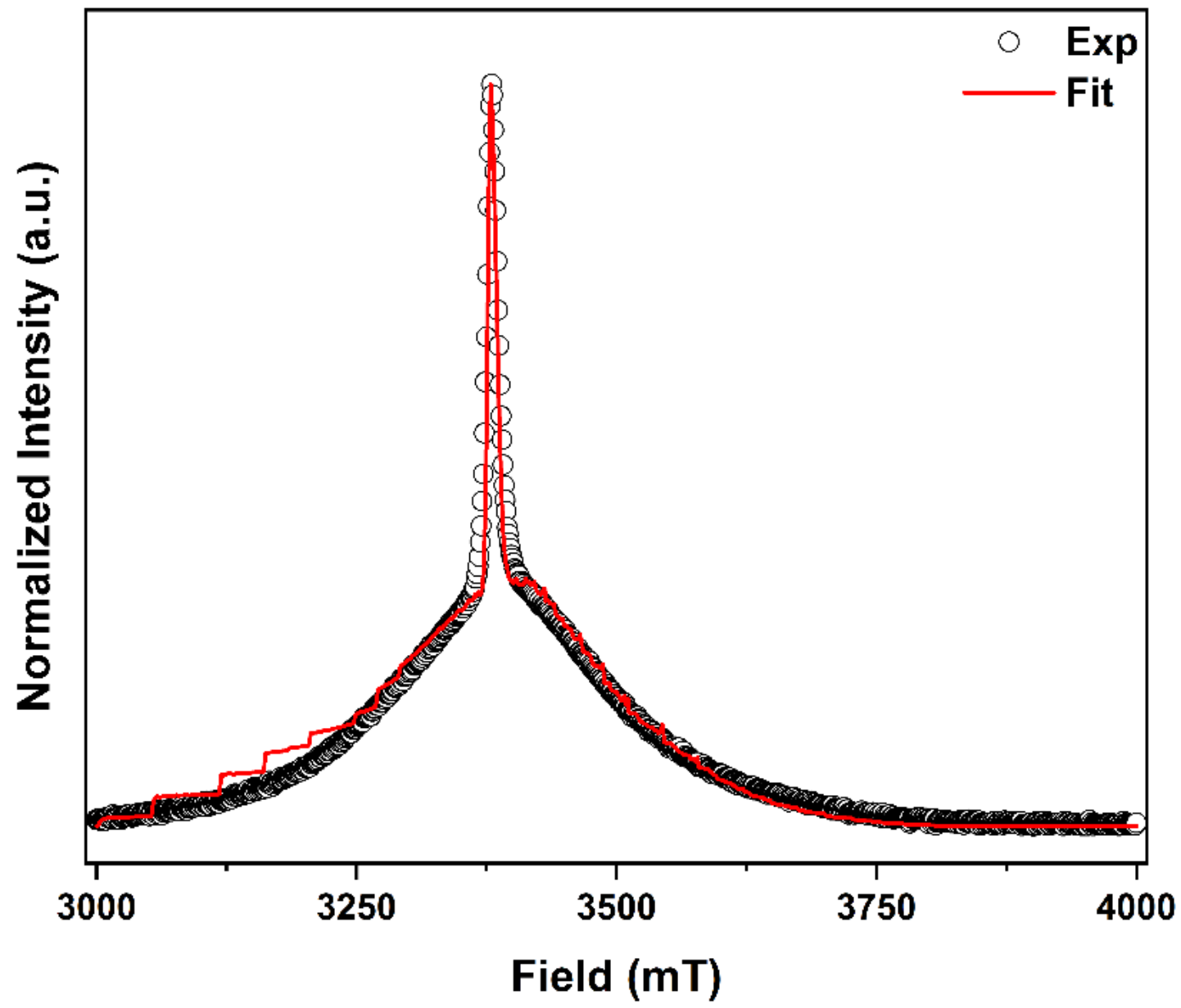

Figure S7. Experimental and fit W-band (94 GHz) EDFS spectra of **1-Eu²⁺** recorded as a 2 mM frozen solution in toluene at 5 K.

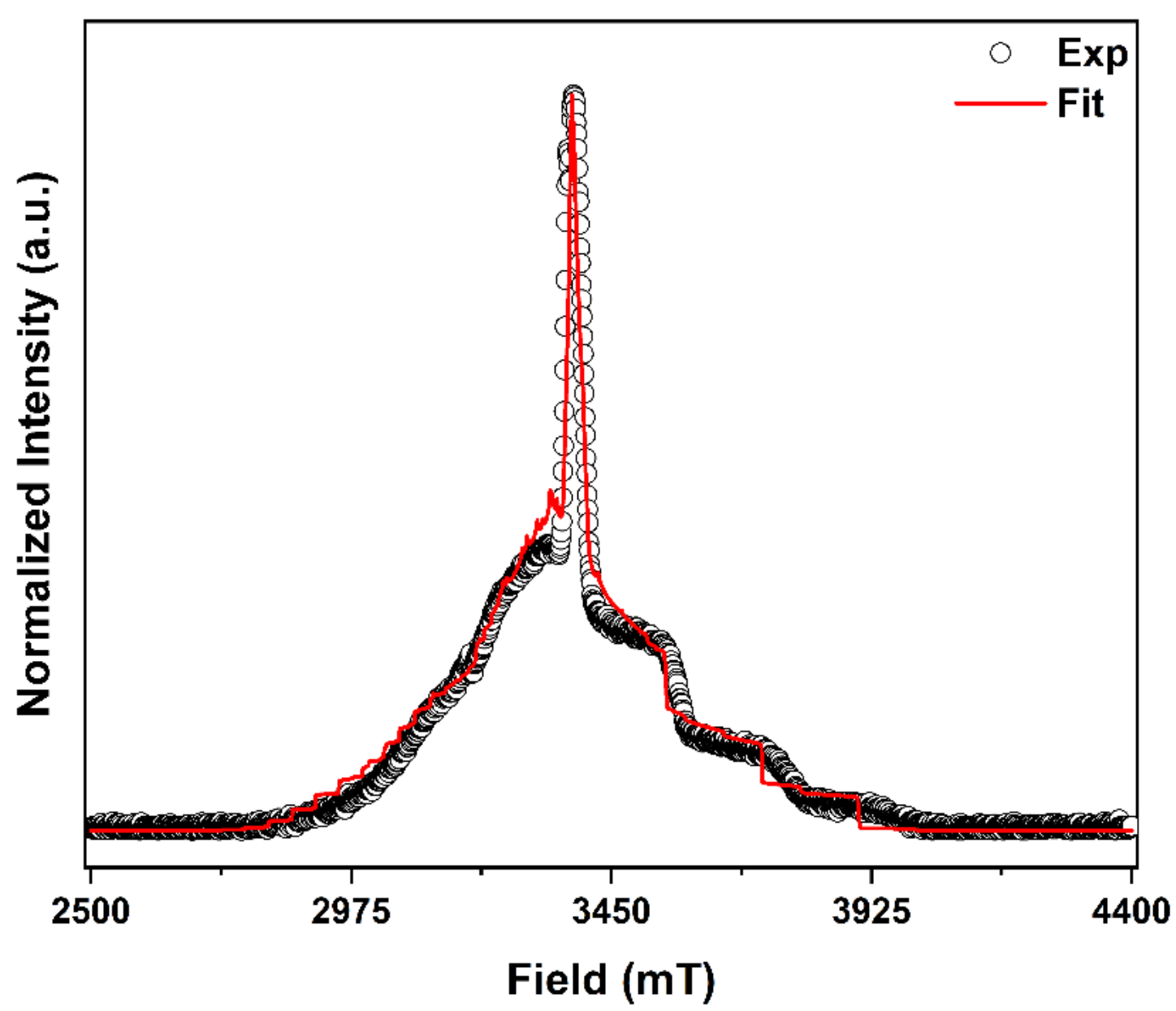

Figure S8. Experimental and fit W-band (94 GHz) EDFS spectra of **2-Gd³⁺** recorded as a 2 mM frozen solution in toluene at 5 K.

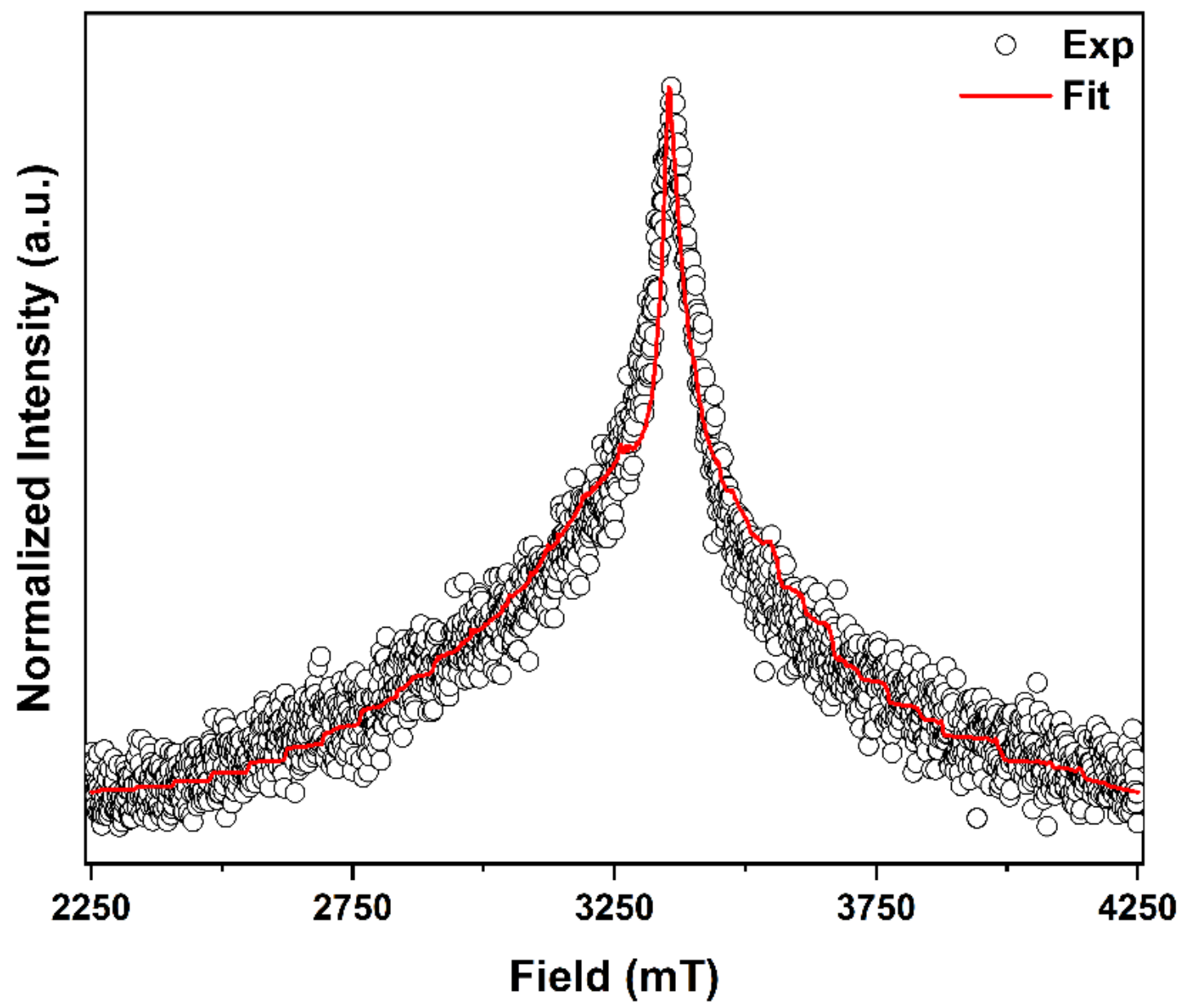

Figure S9. Experimental and fit W-band (94 GHz) EDFS spectra of **4-Tb^{4+}** recorded as a 2 mM frozen solution in toluene (**4-Tb$^{4+}_{tol}$**) at 5 K.

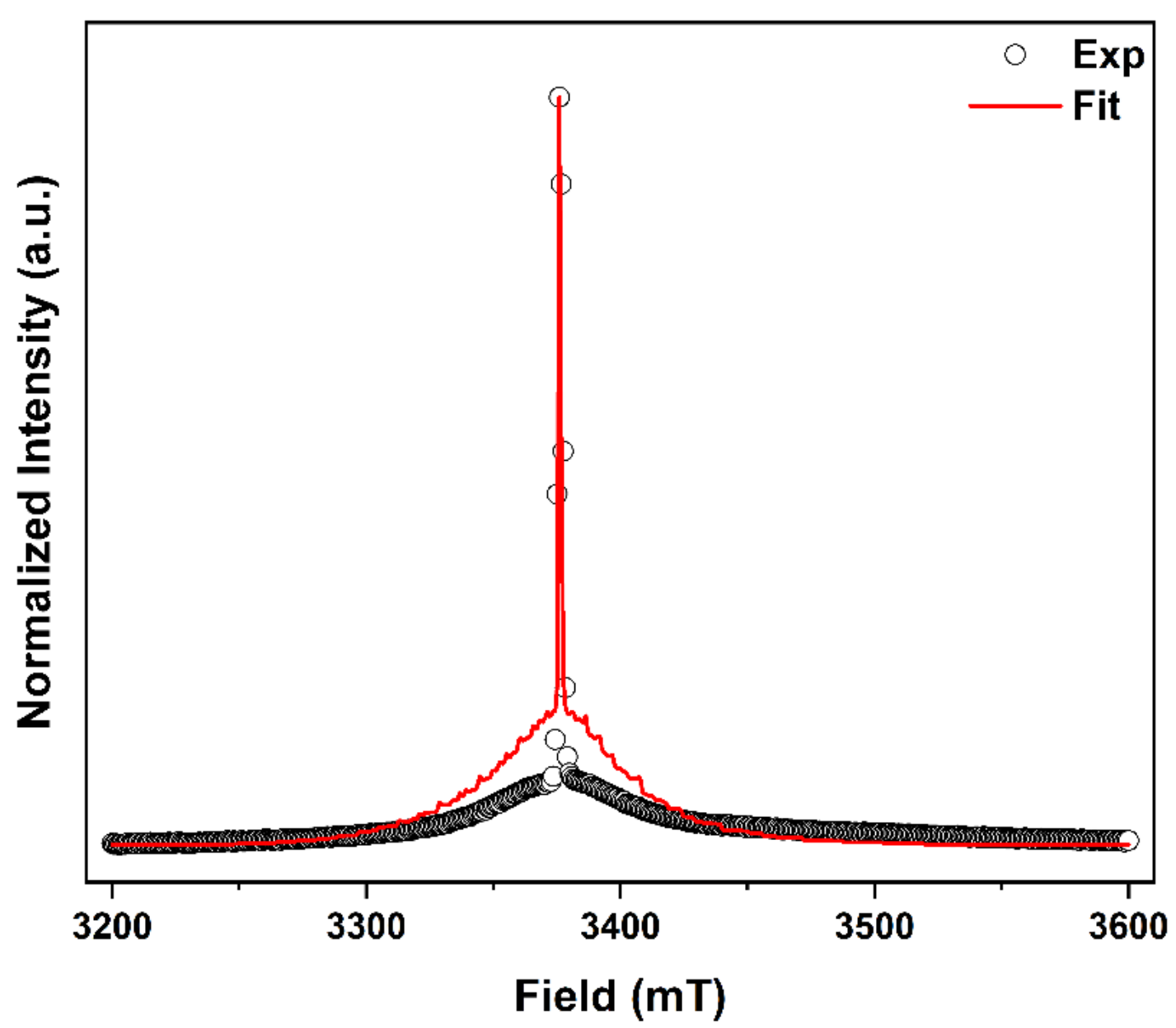

Figure S10. Experimental and fit W-band (94 GHz) EDFS spectra of **3-Gd^{3+}** recorded as a 2 mM frozen solution in 2-MeTHF at 5 K.

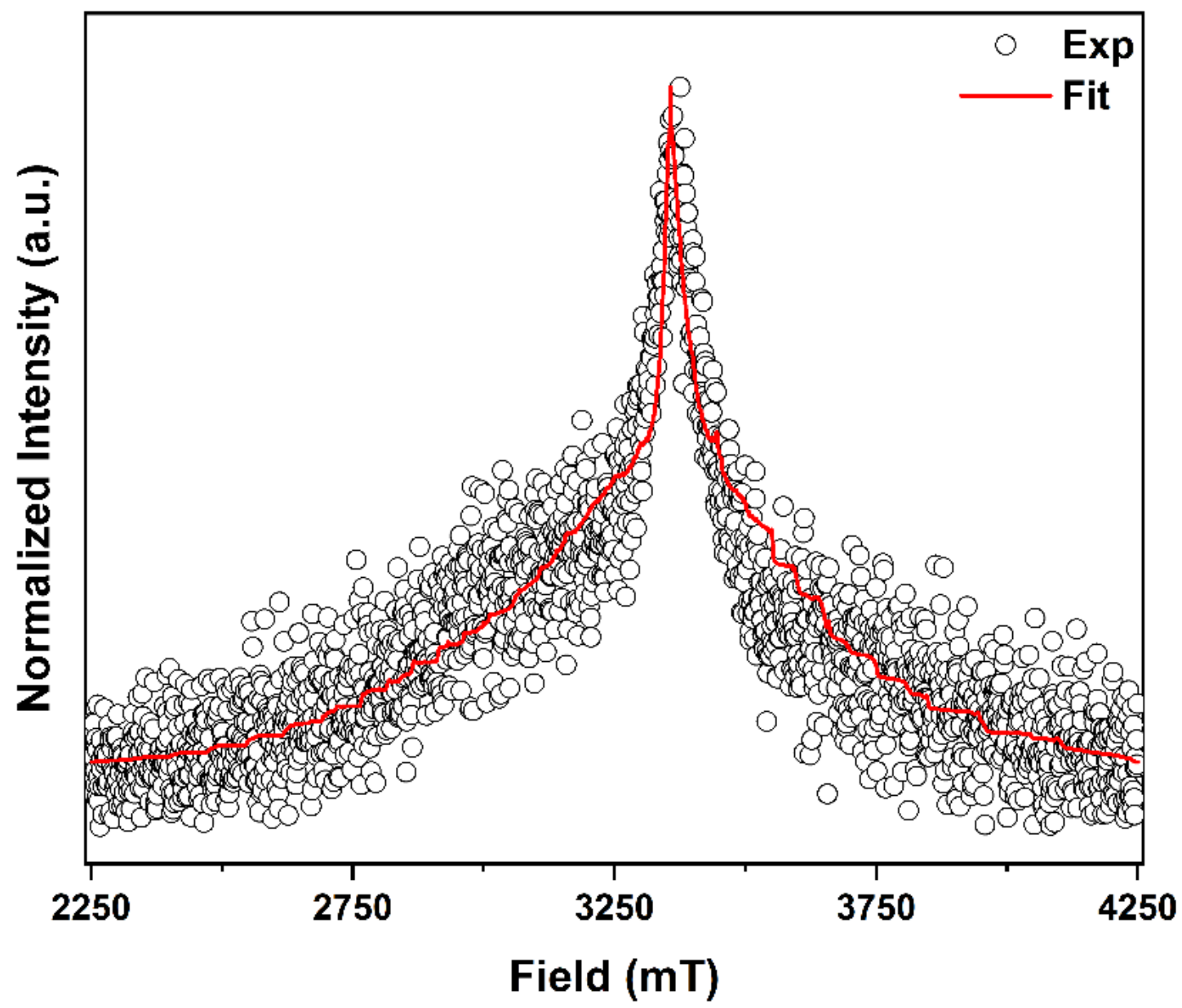

Figure S11. Experimental and fit W-band (94 GHz) EDFS spectra of **4-Tb^{4+}** recorded as a 2 mM frozen solution in 2-MeTHF (**4-Tb$^{4+}_{THF}$**) at 5 K.

As a compromise between numerical precision and computational efficiency, the sampling of the points along the σ_D and σ_E distributions was set at n = 11 points for each distribution, resulting in n^2 = 121 individual spectra being generated and summed to afford the total spectrum for each iteration of the fitting procedure. Though sufficient to enable fitting of the spectra on reasonable timescales, some inherent "steppiness" remains in the final spectra as an artefact of the relatively coarse sampling of the strain distributions as noted in Figures S7-S11. To improve the visual appearance of the final spectra, a single simulation was performed for each dataset sampling n = 101 points along each distribution, resulting in much smoother overall spectra (Figures S12-S16).

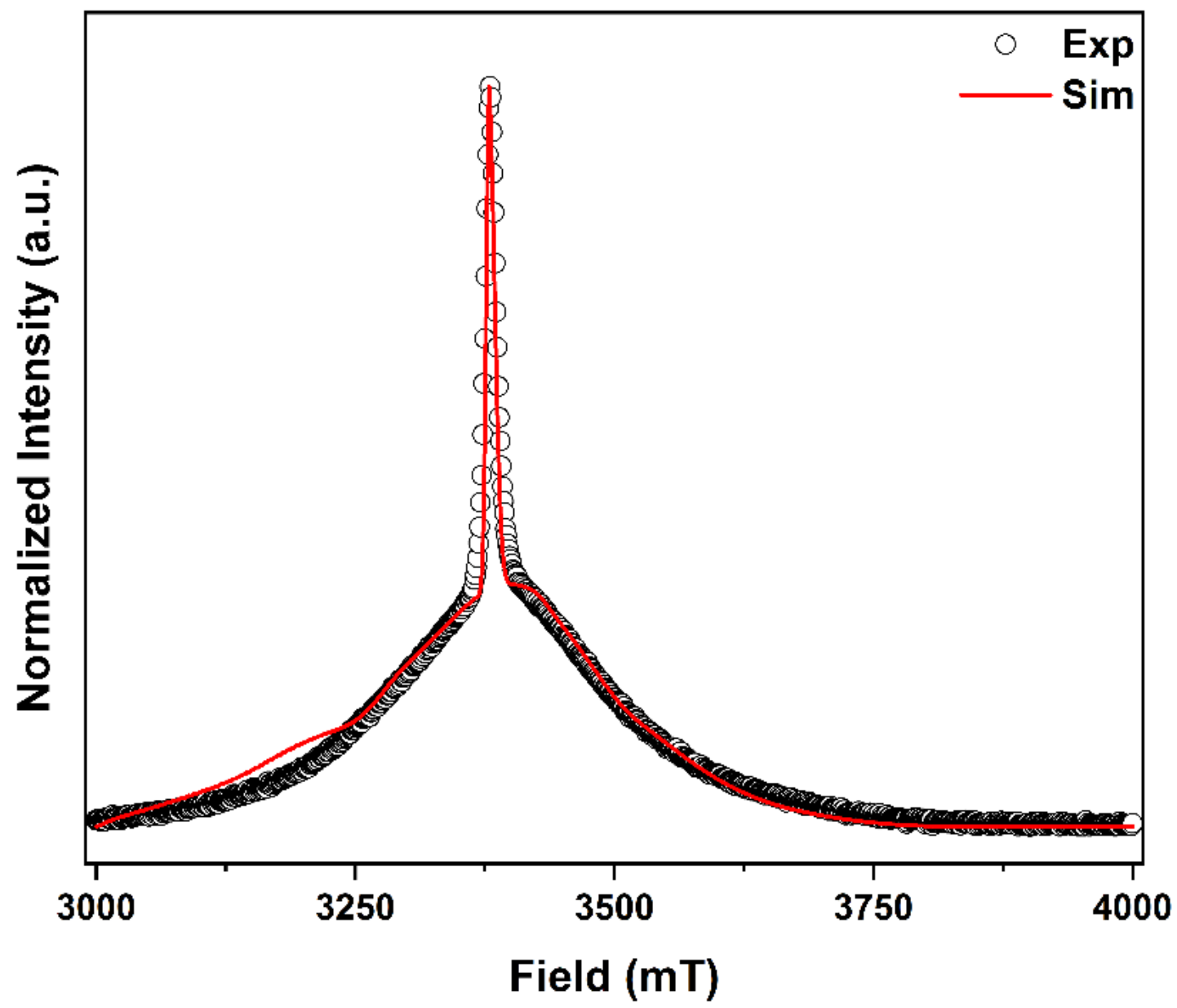

Figure S12. Experimental and simulated (n = 101 points) W-band (94 GHz) EDFS spectra of **1-Eu²⁺** recorded as a 2 mM frozen solution in toluene at 5 K.

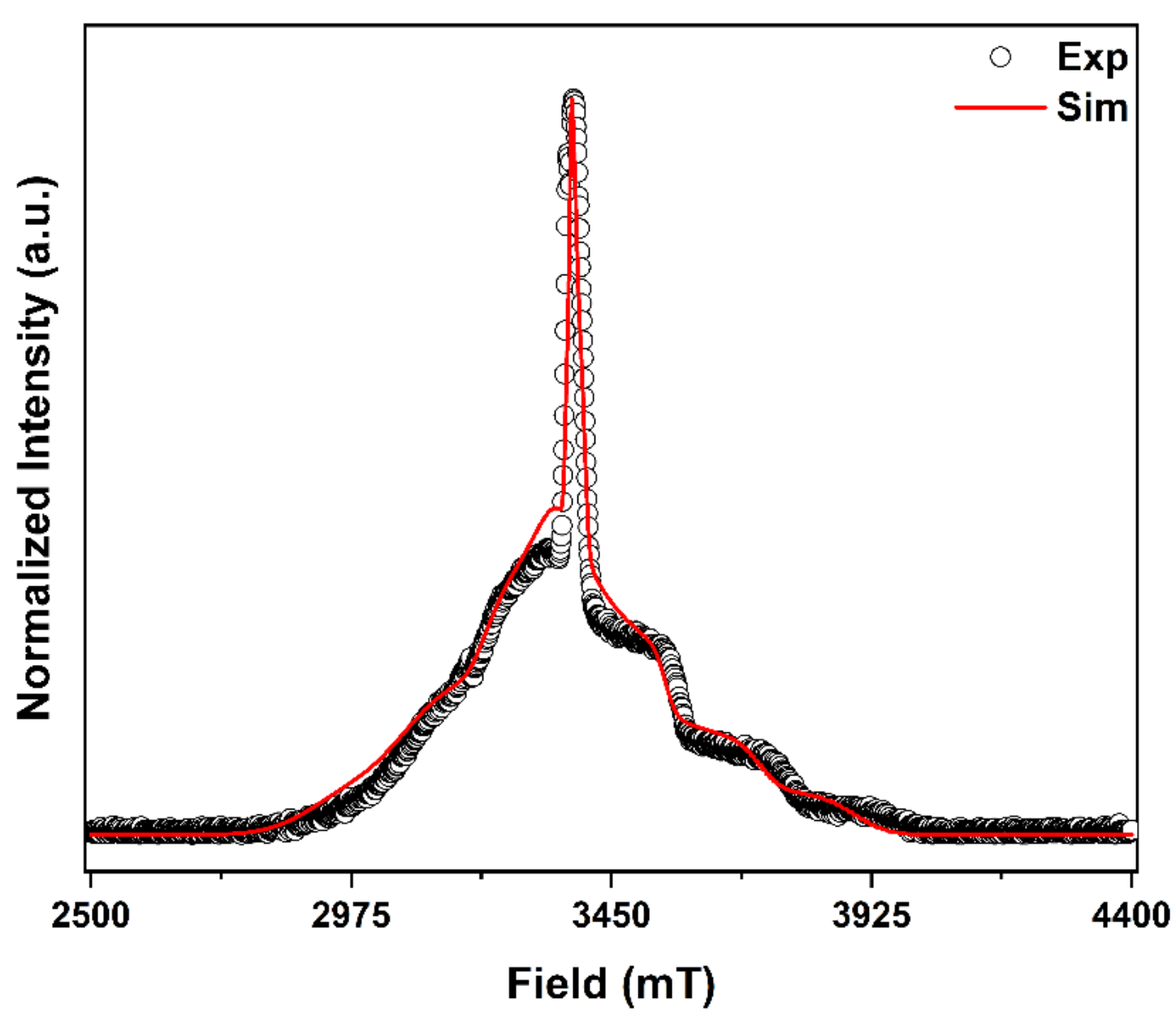

Figure S13. Experimental and simulated (n = 101 points) W-band (94 GHz) EDFS spectra of **2-Gd³⁺** recorded as a 2 mM frozen solution in toluene at 5 K.

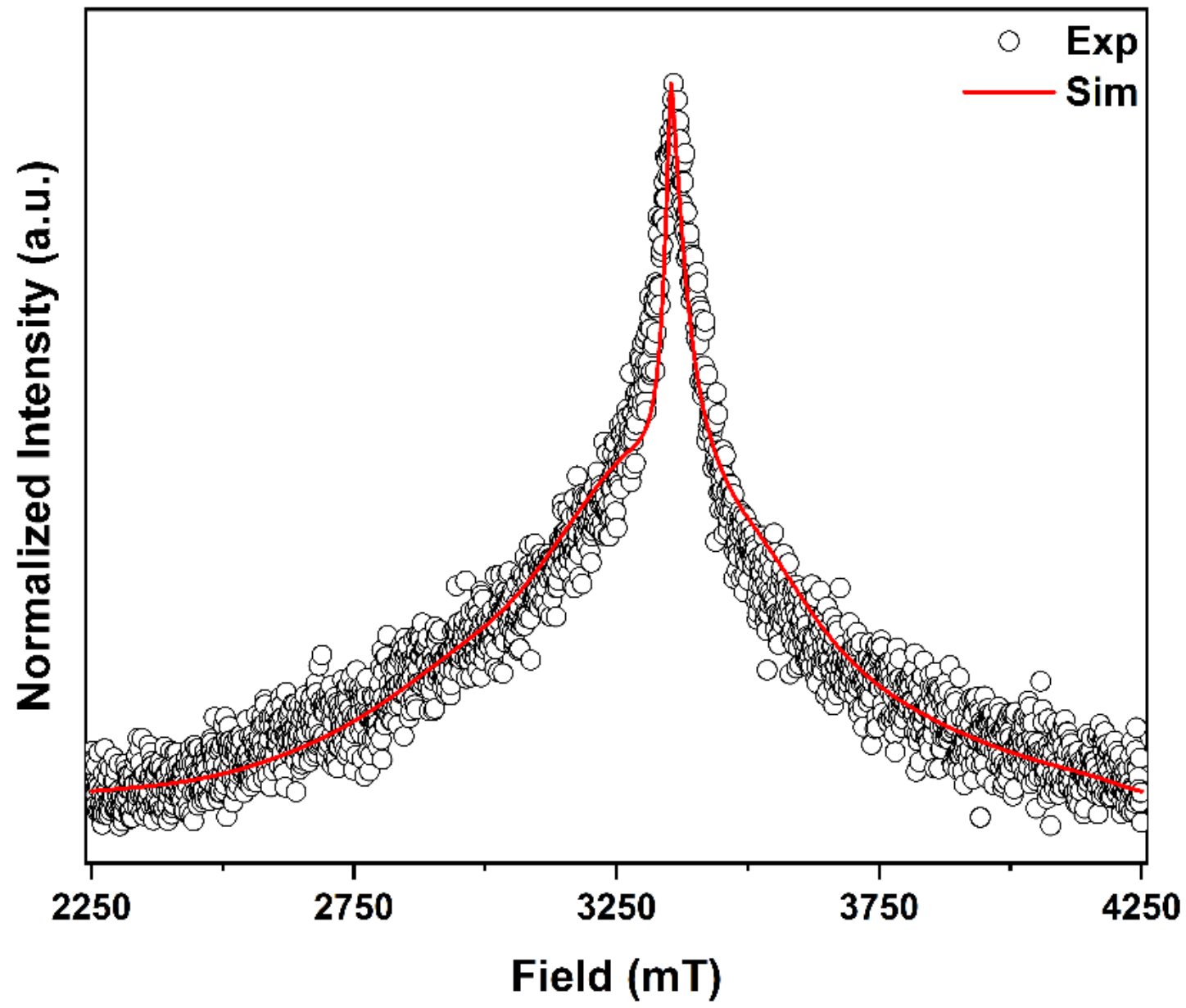

Figure S14. Experimental and simulated (n = 101 points) W-band (94 GHz) EDFS spectra of **4-Tb^{4+}** recorded as a 2 mM frozen solution in toluene (**4-Tb$^{4+}_{tol}$**) at 5 K.

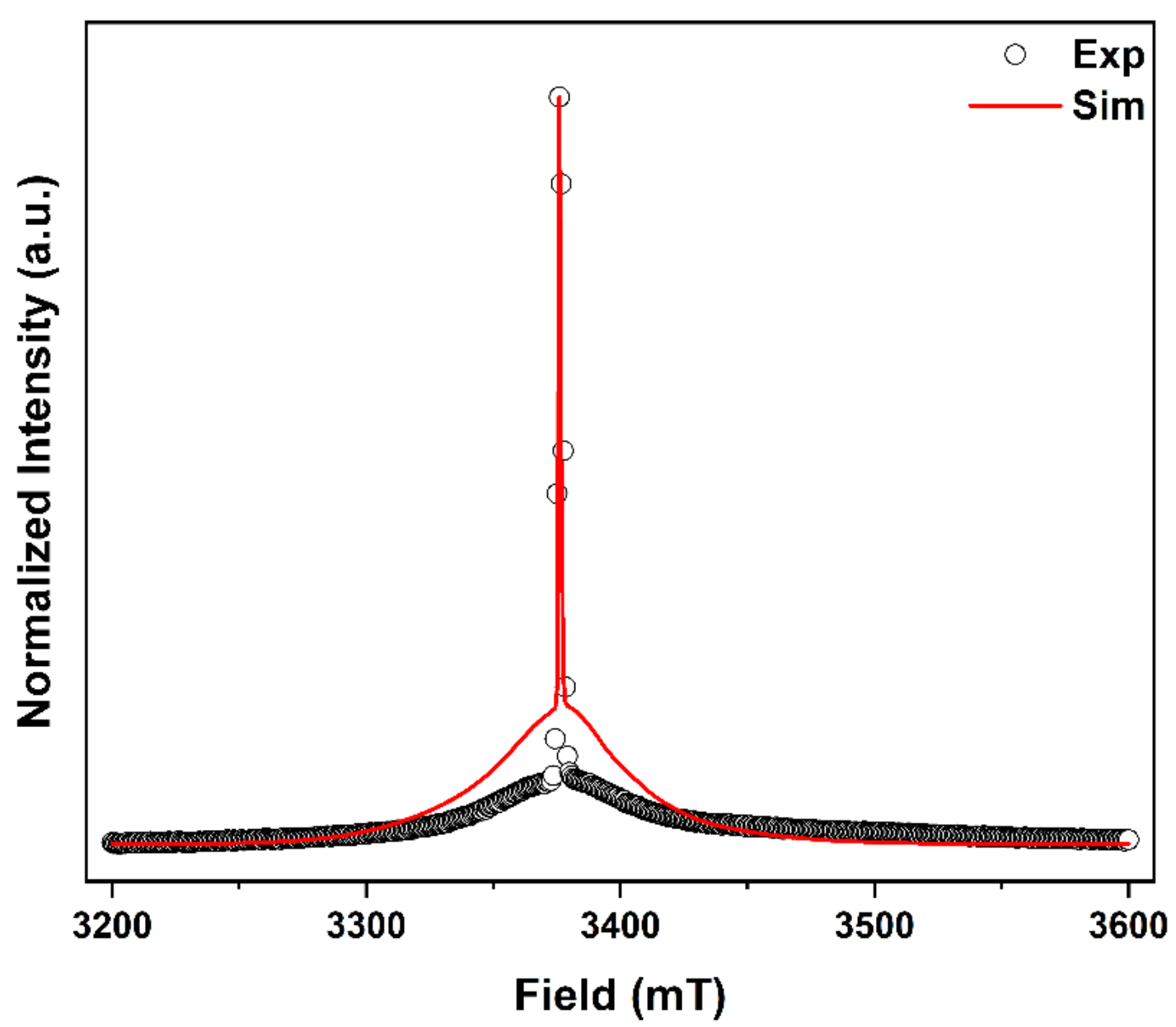

Figure S15. Experimental and simulated (n = 101 points) W-band (94 GHz) EDFS spectra of **3-Gd^{3+}** recorded as a 2 mM frozen solution in 2-MeTHF at 5 K.

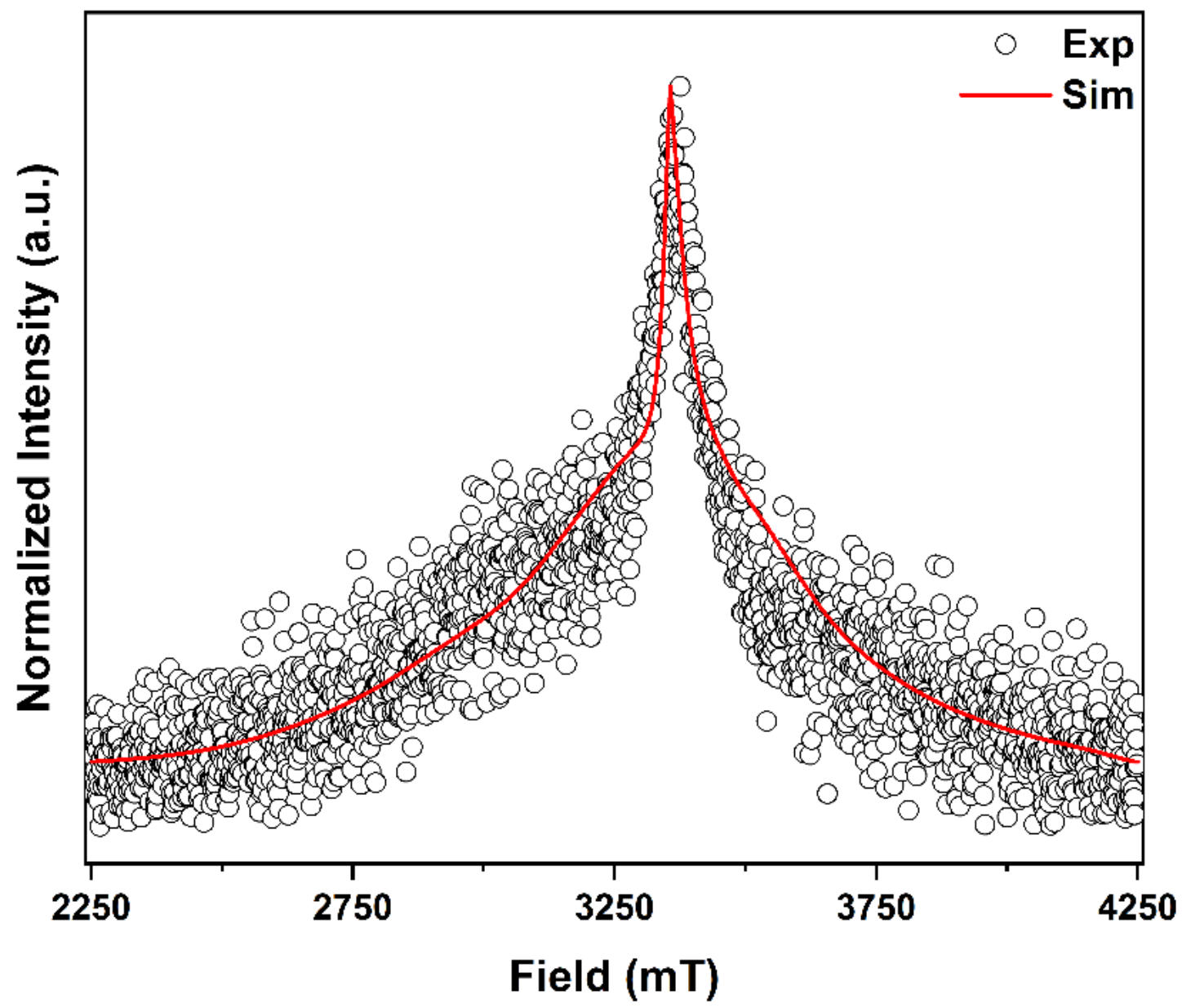

Figure S16. Experimental and simulated (n = 101 points) W-band (94 GHz) EDFS spectra of **4-Tb^{4+}** recorded as a 2 mM frozen solution in 2-MeTHF (**4-Tb$^{4+}_{THF}$**) at 5 K.

The spectrum of an empty sample holder assembly was also recorded and displays a single, broad, weak signal (Figure S17). The spectrum was normalized and fit utilizing the same fitting procedures as the analyte spectra with an offset baseline and spectral line broadening applied via an isotropic Lorentzian component. The following S = 1/2 spin Hamiltonian was used to fit the blank spectrum:

$$\hat{H}_{spin} = g_{iso}\mu_B\vec{\boldsymbol{B}}\cdot\hat{\boldsymbol{S}} \quad \text{(S2)}$$

Where the variables take the same meaning as in Eqn. S1. The spectrum was best fit with g_{iso} = 1.9957(13) with a full width at half maximum (FWHM) Lorentzian peak broadening of 40(2) mT. This feature is likely due to a paramagnetic impurity in the H grease and does not coincide with the central transition features of the analyte spectra, nor is it readily apparent in other regions of those spectra.

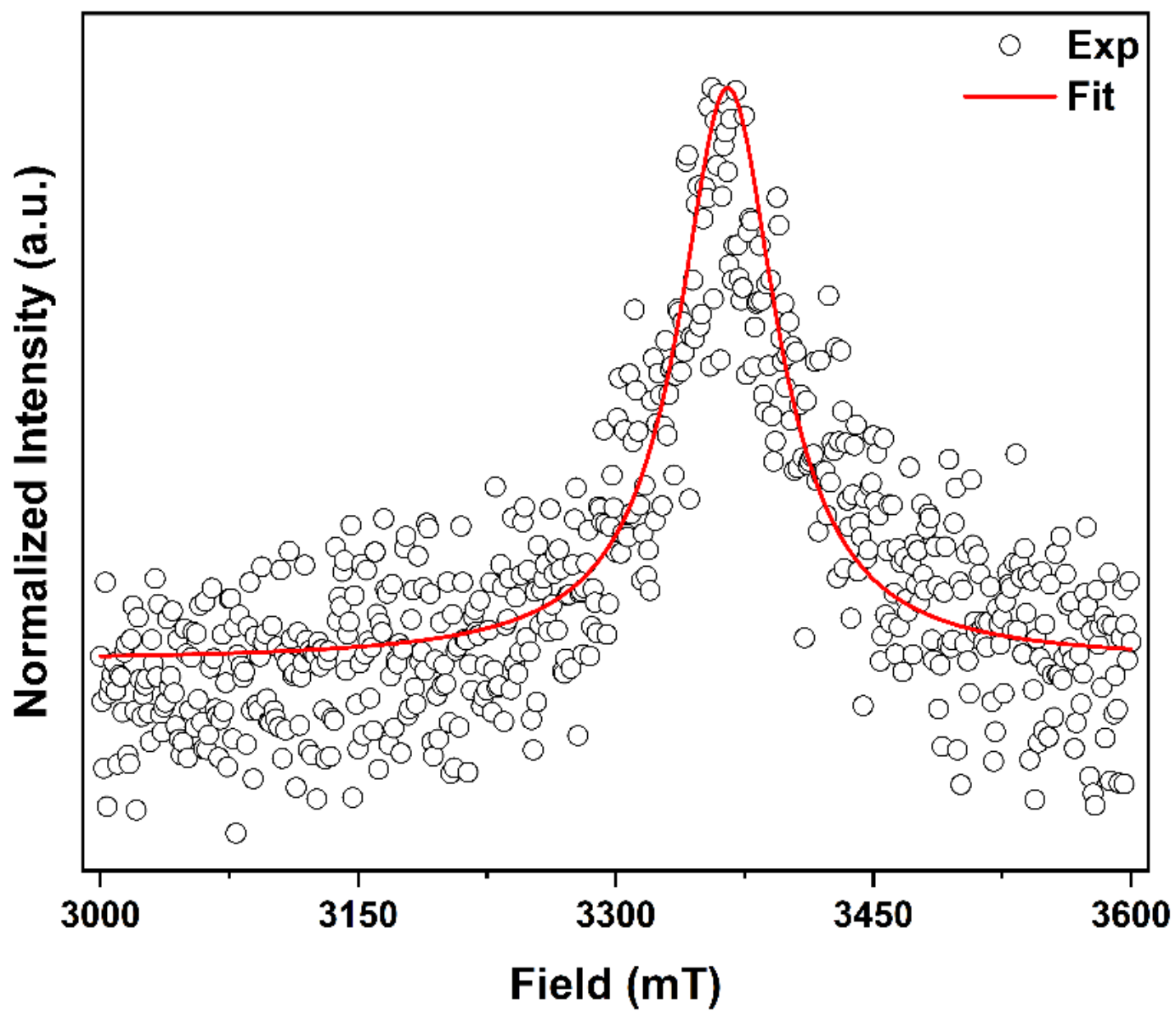

Figure S17. Experimental and fit W-band (94 GHz) EDFS spectra of blank sample holder recorded at 5 K.

***T_1* and *T_m* Measurements.** Variable-temperature T_1 and T_m measurements were performed for each analyte at the field of maximum echo intensity corresponding to the central ($m_s = -1/2 \rightarrow m_s = 1/2$) transition (**1-Eu^{2+}**: 3.375 T; **2-Gd^{3+}**: 3.382 T; **3-Gd^{3+}**: 3.371 T; **4-Tb^{4+}$_{tol}$**: 3.358 T; **4-Tb^{4+}$_{THF}$**: 3.368 T). Standard inversion recovery ($\pi - t_{wait} - \frac{\pi}{2} - \tau - \pi - \tau - echo$) and Hahn echo ($\frac{\pi}{2} - \tau - \pi - \tau - echo$) experiments were utilized to measure T_1 and T_m respectively at each temperature. Pulse parameters were optimized for each temperature and sample to maximize the echo intensity with typical values of $\frac{\pi}{2}$ ranging 40-100 ns. As was done for the EDFS data, the resulting magnitude of the real and imaginary signal components was normalized and fit with a standard monoexponential function of the form:

$$I(t) = I_0 + Ae^{-x/T} \tag{S3}$$

Where I is the signal intensity, I_0 the intensity offset, A is a scaling factor, T is the characteristic time (T_1 or T_m) and $x = t_{wait}$ for T_1 measurements, and $x = 2\tau$ for T_m measurements. Fits were performed via least-squares curve fitting as implemented in the *lmfit* package in python using a home-built code.[22] For inversion recovery measurements, it was occasionally necessary to manually invert the first few points in the measurement prior to the normalization process. Each measurement was repeated at least twice at each temperature, and the presented values are arithmetic averages of the results with the propagated standard deviations from the fitting procedures. Measurements were begun at 5 K and continued with increasing temperature until the echo became too weak to produce meaningful data. For **4-Tb^{4+}$_{tol}$** and **4-Tb^{4+}$_{THF}$**, additional data points were obtained at 3 and 4 K (as applicable) to supplement the small number of data points ≥ 5 K. The results of the measurements are tabulated in Tables S4 and S5, and the plots of T_1 and T_m vs temperature for each analyte are given in Figures S18-S22.

Table S4. T_1 and T_m average fit results for **1-Eu^{2+}**, **2-Gd^{3+}**, and **4-Tb$^{4+}_{tol}$**.

	1-Eu^{2+}		***2-Gd^{3+}***		***4-Tb$^{4+}_{tol}$***	
Temperature (K)	*T_1 (μs)*	*T_m (μs)*	*T_1 (μs)*	*T_m (μs)*	*T_1 (μs)*	*T_m (μs)*
3	-	-	-	-	-	0.351(6)
4	-	-	-	-	3.72(15)	0.252(5)
5	2.12(8)	0.698(5)	3.64(8)	0.895(12)	2.95(13)	0.222(9)
6	-	-	-	-	2.6(4)	0.173(18)
7	-	-	-	-	2.4(6)	0.18(2)
7.5	1.83(12)	0.501(3)	2.52(8)	0.622(7)	-	-
8	-	-	-	-	2.2(9)	0.16(4)
9	-	-	-	-	2.1(7)	0.14(5)
10	1.54(6)	0.399(4)	2.52(8)	0.491(6)	1.5(10)	-
12.5	1.23(13)	0.335(7)	1.96(10)	0.403(5)	-	-
15	1.05(14)	0.284(9)	1.61(14)	0.338(7)	-	-
17.5	0.94(17)	0.25(1)	1.36(16)	0.296(9)	-	-
20	0.76(4)	0.203(18)	1.17(12)	0.245(16)	-	-
22.5	0.58(4)	0.17(3)	0.94(11)	0.222(11)	-	-
25	0.49(7)	0.170(16)	0.86(12)	0.188(17)	-	-
27.5	0.39(7)	0.150(3)	0.64(17)	0.16(2)	-	-
30	0.35(8)	0.149(5)	0.71(7)	0.173(8)	-	-
32.5	-	0.124(19)	-	-	-	-
35	-	0.109(14)	0.55(16)	0.147(11)	-	-
40	-	-	0.45(5)	0.119(13)	-	-
45	-	-	0.36(5)	0.10(3)	-	-
50	-	-	0.33(12)	0.076(16)	-	-

Table S5. T_1 and T_m average fit results for **3-Gd^{3+}** and **4-Tb^{4+}$_{THF}$**.

	3-Gd^{3+}		***4-Tb^{4+}$_{THF}$***	
Temperature (K)	*T_1 (μs)*	*T_m (μs)*	*T_1 (μs)*	*T_m (μs)*
4	-	-	3.46(15)	0.241(7)
5	7.95(12)	0.97(3)	3.4(3)	0.211(9)
6	-	-	2.7(8)	0.16(4)
7	-	-	2.5(6)	0.17(4)
7.5	6.27(10)	0.757(9)	-	-
8	-	-	2.6(6)	0.14(6)
9	-	-	-	0.14(7)
10	4.99(7)	0.645(6)	-	-
12.5	3.99(5)	0.558(4)	-	-
15	3.17(5)	0.491(3)	-	-
17.5	2.47(7)	0.439(3)	-	-
20	1.84(4)	0.390(3)	-	-
22.5	1.66(4)	0.356(4)	-	-
25	1.44(3)	0.320(5)	-	-
27.5	1.22(2)	0.285(6)	-	-
30	1.03(4)	0.250(11)	-	-
35	0.82(8)	0.256(4)	-	-
40	0.66(6)	0.208(6)	-	-
45	0.52(3)	0.172(14)	-	-
50	0.46(7)	0.136(17)	-	-
55	0.37(3)	0.146(8)	-	-
60	0.32(6)	0.11(4)	-	-
70	0.29(5)	0.110(5)	-	-
80	0.20(7)	0.066(16)	-	-
90	-	0.028(19)	-	-

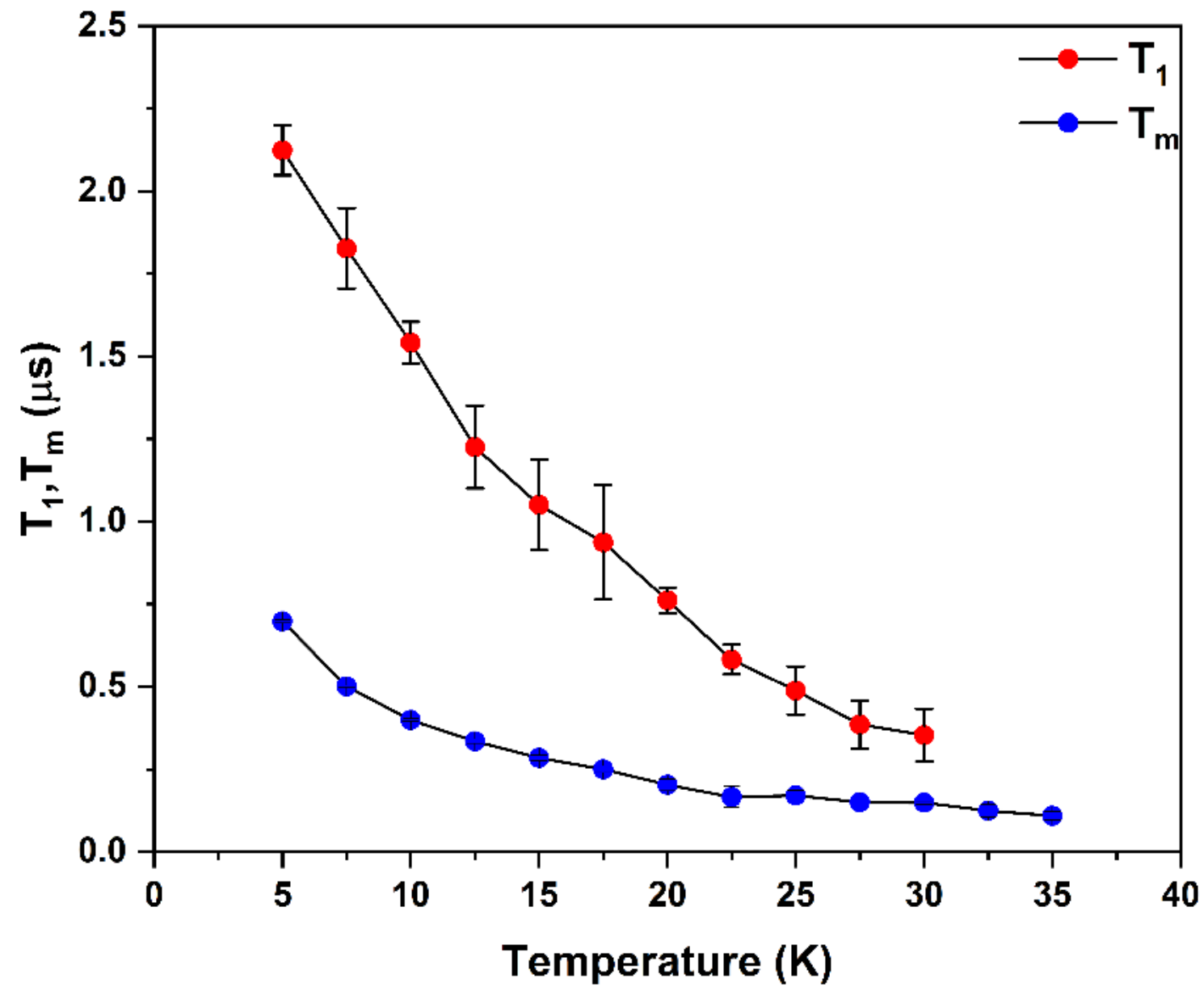

Figure S18. Average T_1 (red) and T_m (blue) fit result values and errors for **1-Eu²⁺**. Lines between points do not represent a fit but rather serve as a visual guide.

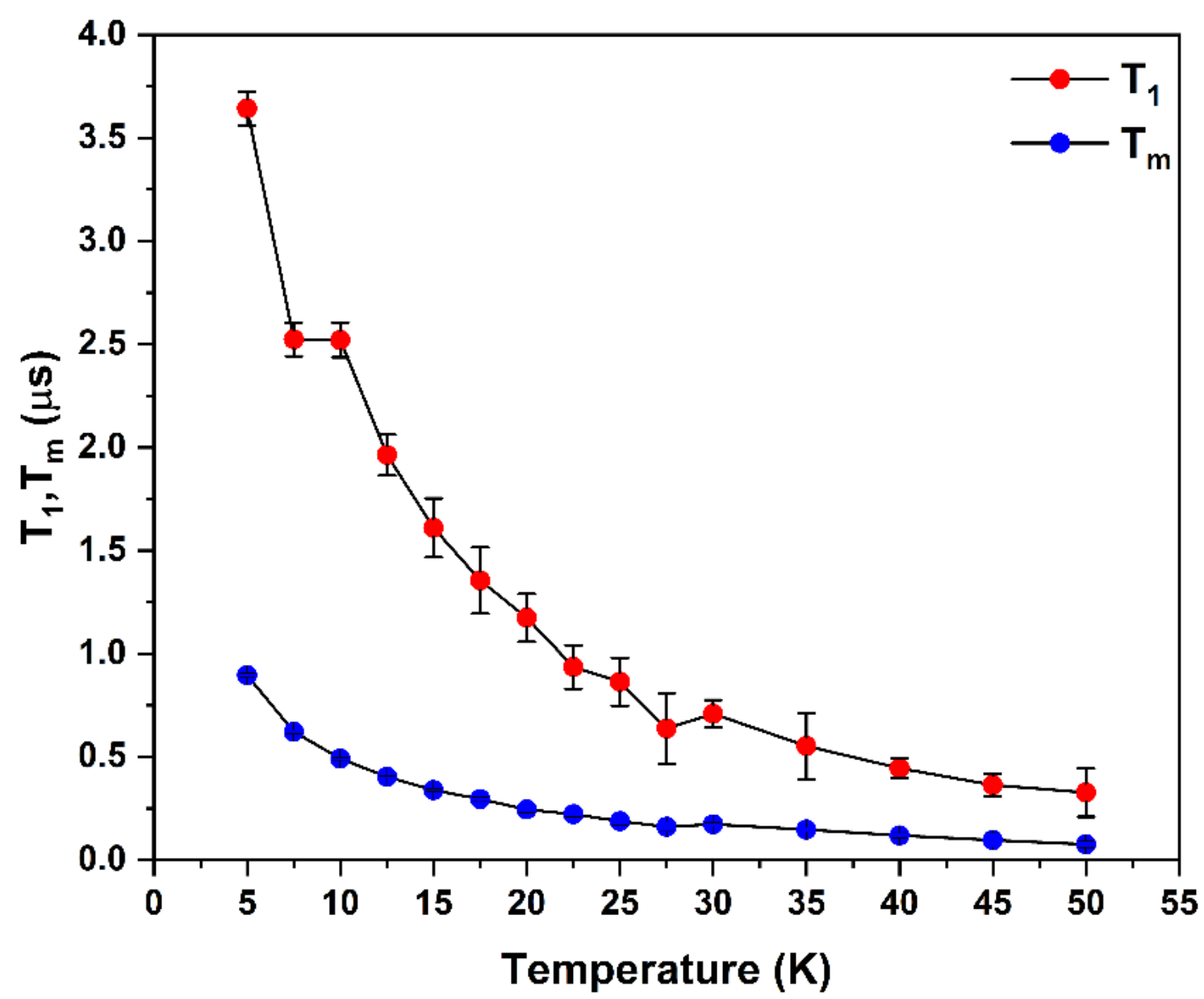

Figure S19. Average T_1 (red) and T_m (blue) fit result values and errors for **2-Gd³⁺**. Lines between points do not represent a fit but rather serve as a visual guide.

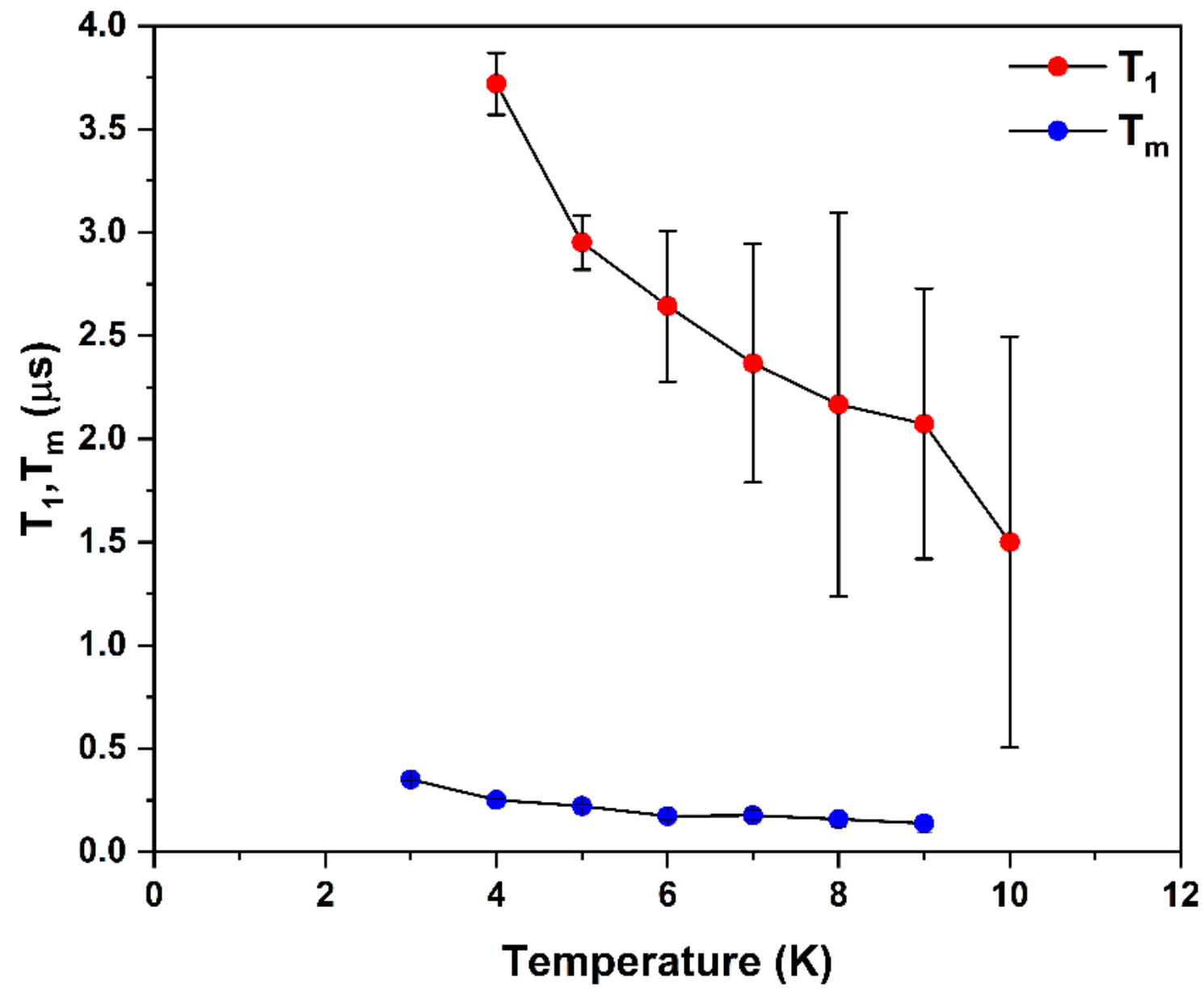

Figure S20. Average T_1 (red) and T_m (blue) fit result values and errors for **4-Tb^{4+}$_{tol}$**. Lines between points do not represent a fit but rather serve as a visual guide.

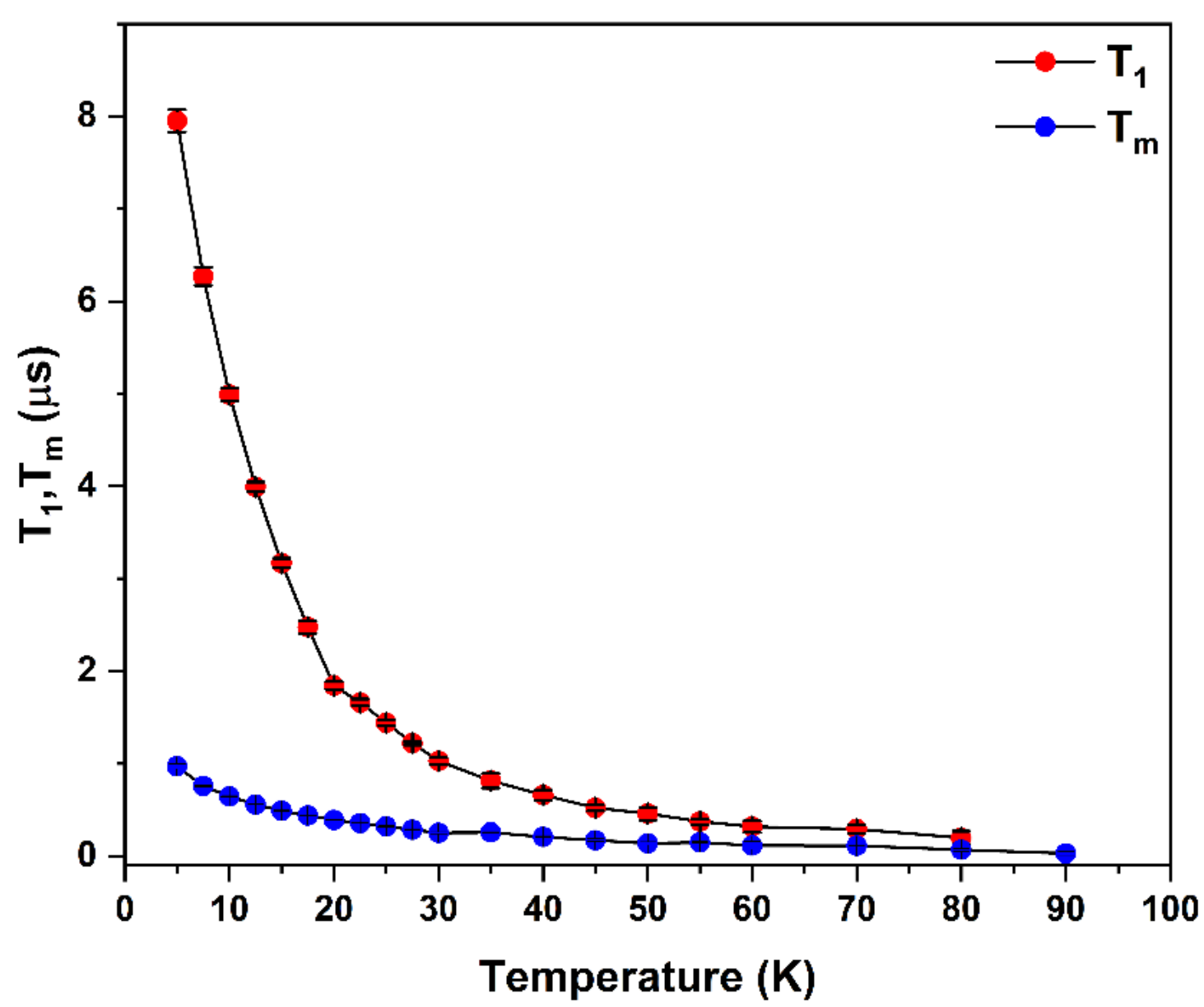

Figure S21. Average T_1 (red) and T_m (blue) fit result values and errors for **3-Gd^{3+}**. Lines between points do not represent a fit but rather serve as a visual guide.

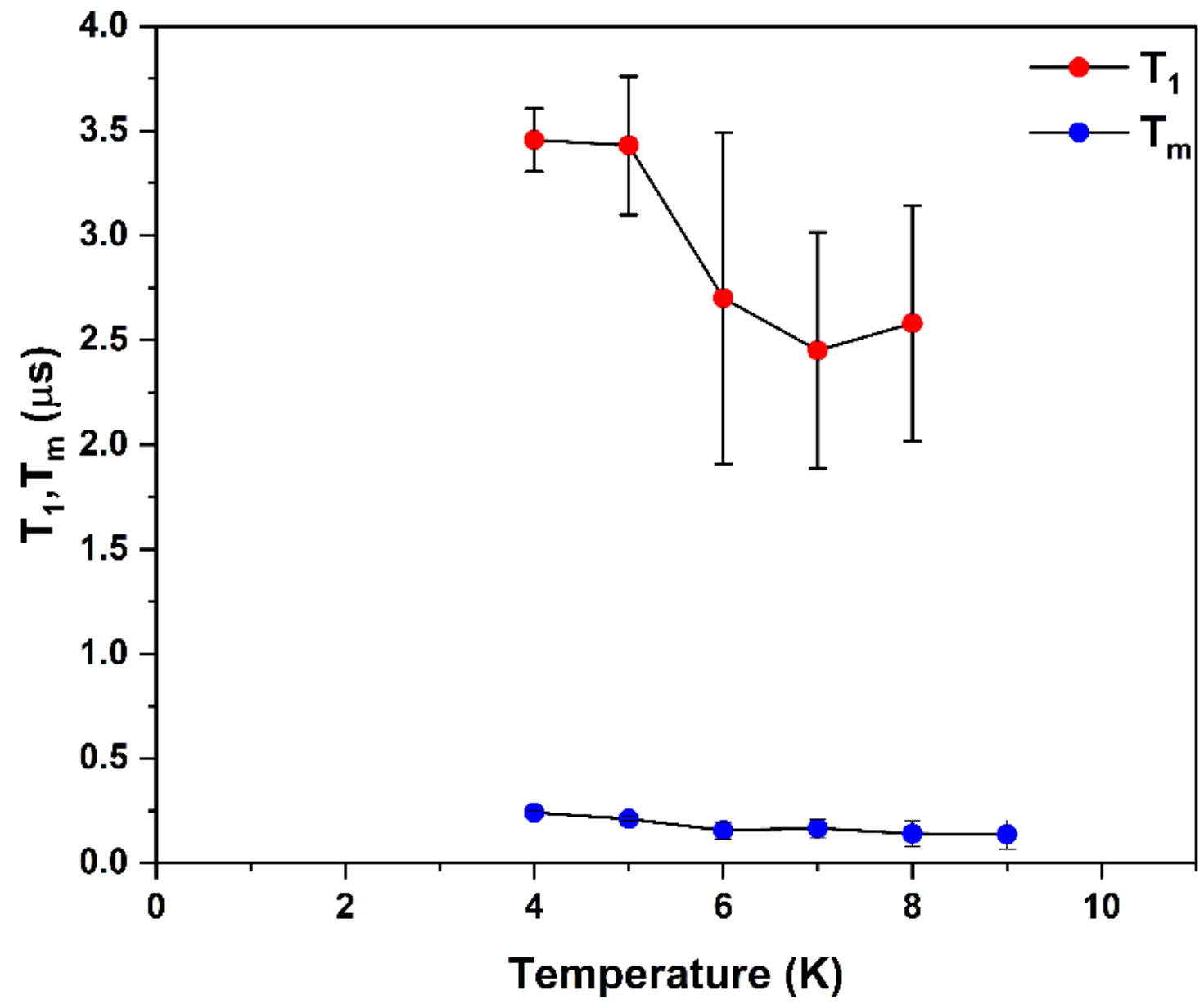

Figure S22. Average T_1 (red) and T_m (blue) fit result values and errors for **4-Tb^{4+}$_{THF}$**. Lines between points do not represent a fit but rather serve as a visual guide.

***T_1* Power Law Fits.** To determine the temperature dependencies of T_1 for the analytes, plots of log(1/T_1) vs. log(temperature) were generated and each distinct linear regime for each compound was fit to extract the underlying temperature dependence (Figures S23-S27).

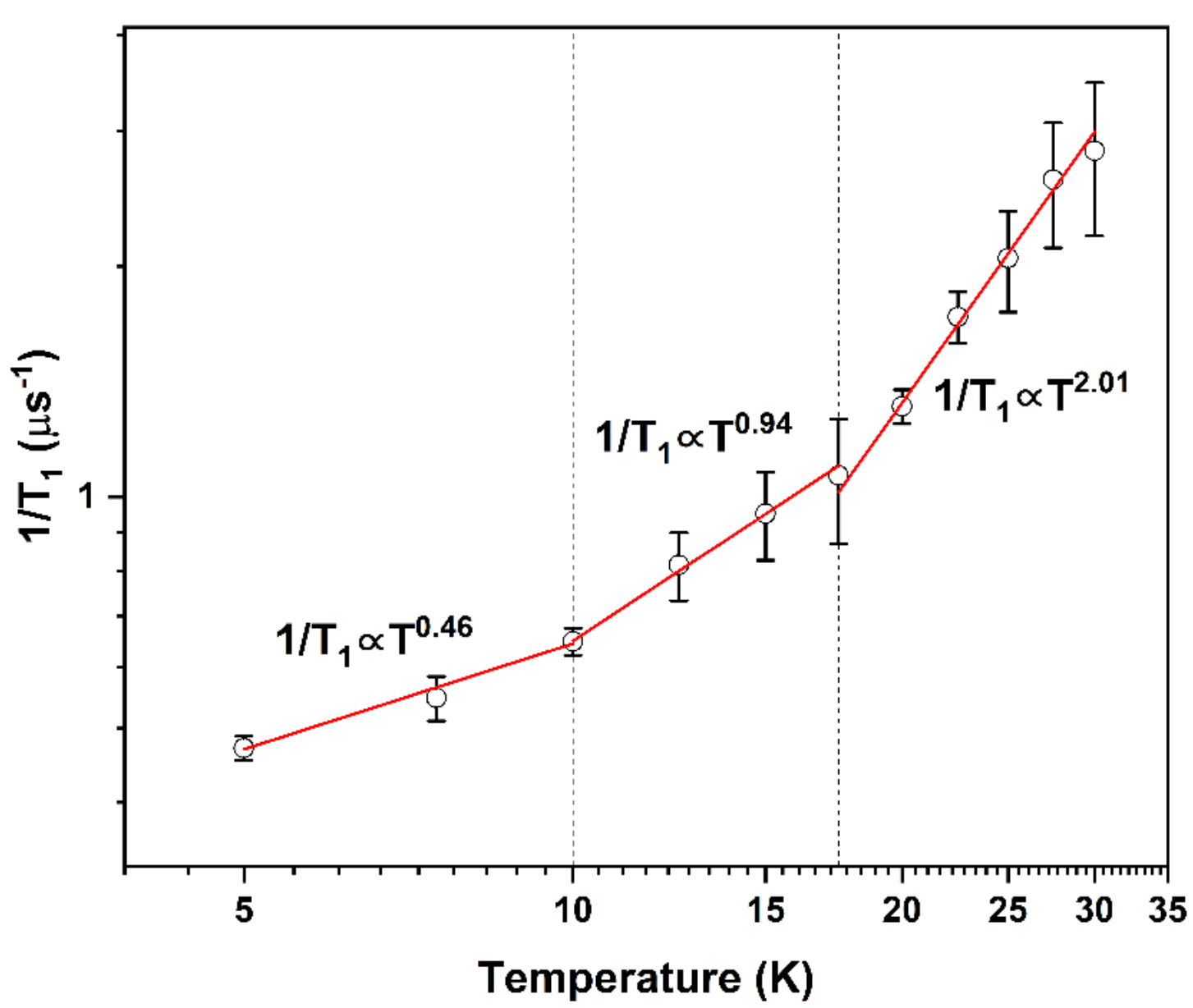

Figure S23. Plot of log(1/T_1) vs. log(temperature) for **1-Eu^{2+}** and corresponding linear fits (red). The distinct regions are indicated by the vertical dashed lines and the dependence of 1/T_1 as a function of temperature is given for each region.

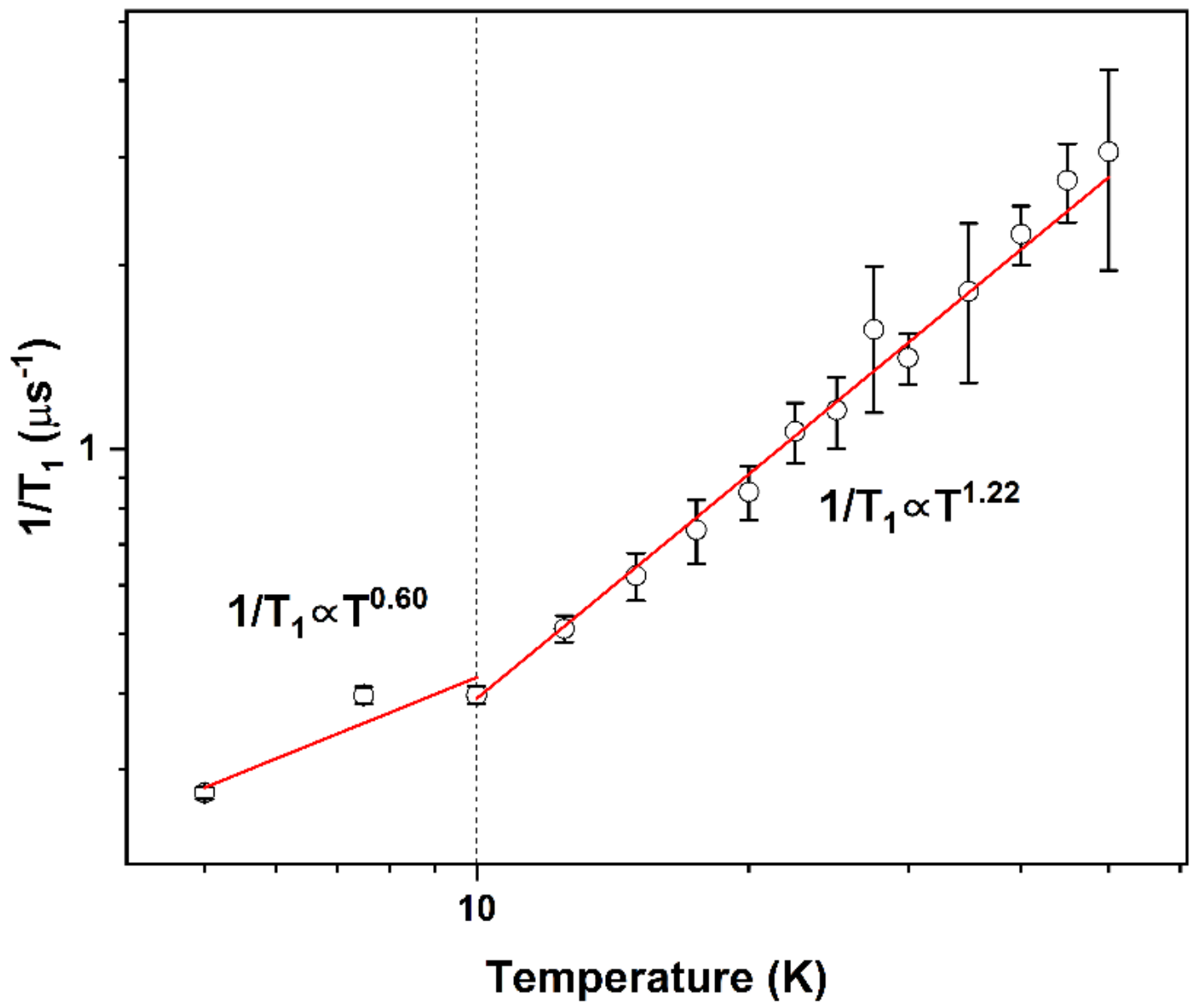

Figure S24. Plot of log(1/T_1) vs. log(temperature) for **2-Gd^{3+}** and corresponding linear fits (red). The distinct regions are indicated by the vertical dashed line and the dependence of 1/T_1 as a function of temperature is given for each region.

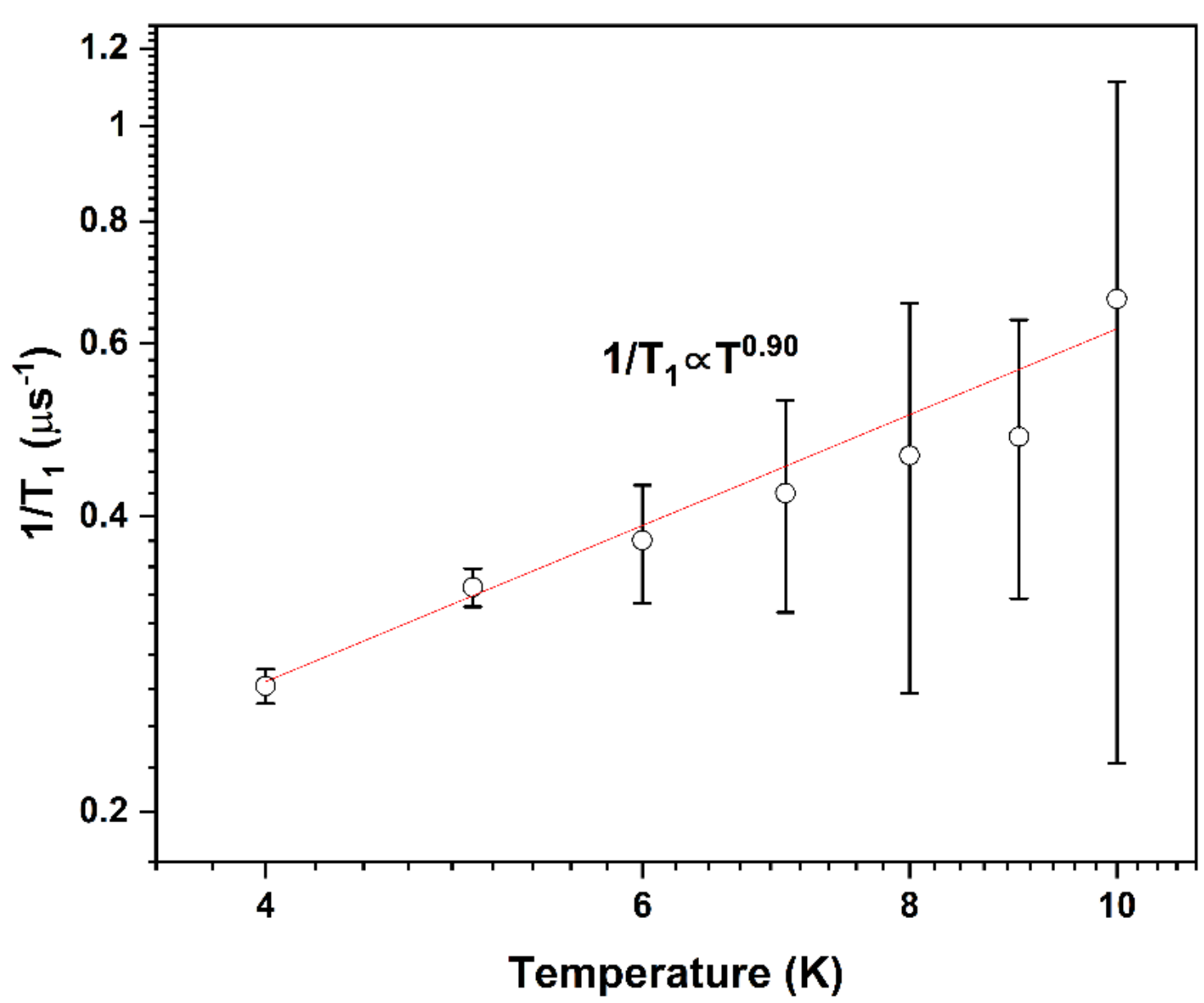

Figure S25. Plot of log(1/T_1) vs. log(temperature) for **4-Tb$^{4+}_{tol}$** and corresponding linear fit (red). The dependence of 1/T_1 as a function of temperature extracted from the fit is given in the plot.

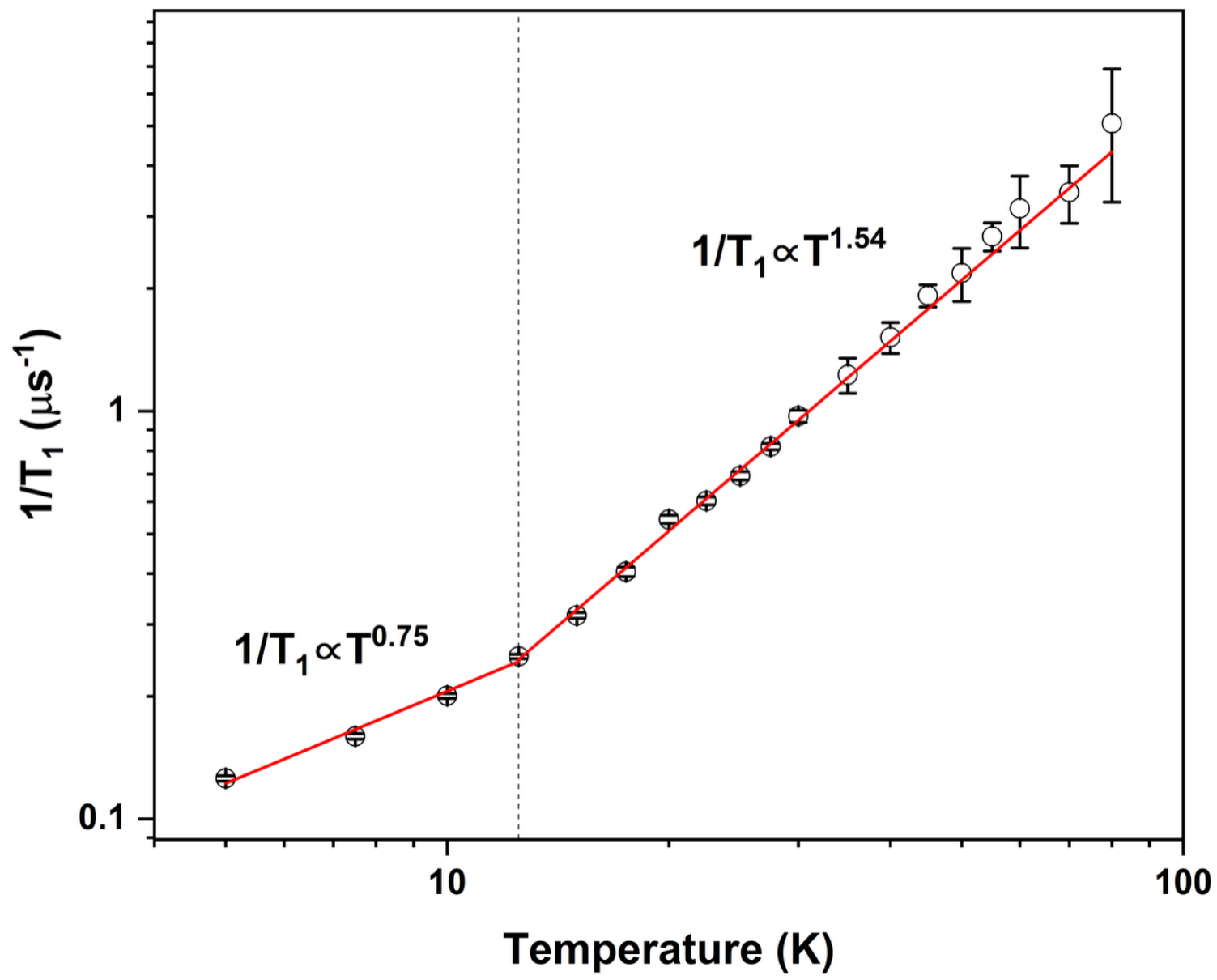

Figure S26. Plot of log(1/T_1) vs. log(temperature) for **3-Gd³⁺** and corresponding linear fits (red). The distinct regions are indicated by the vertical dashed line and the dependence of 1/T_1 as a function of temperature is given for each region.

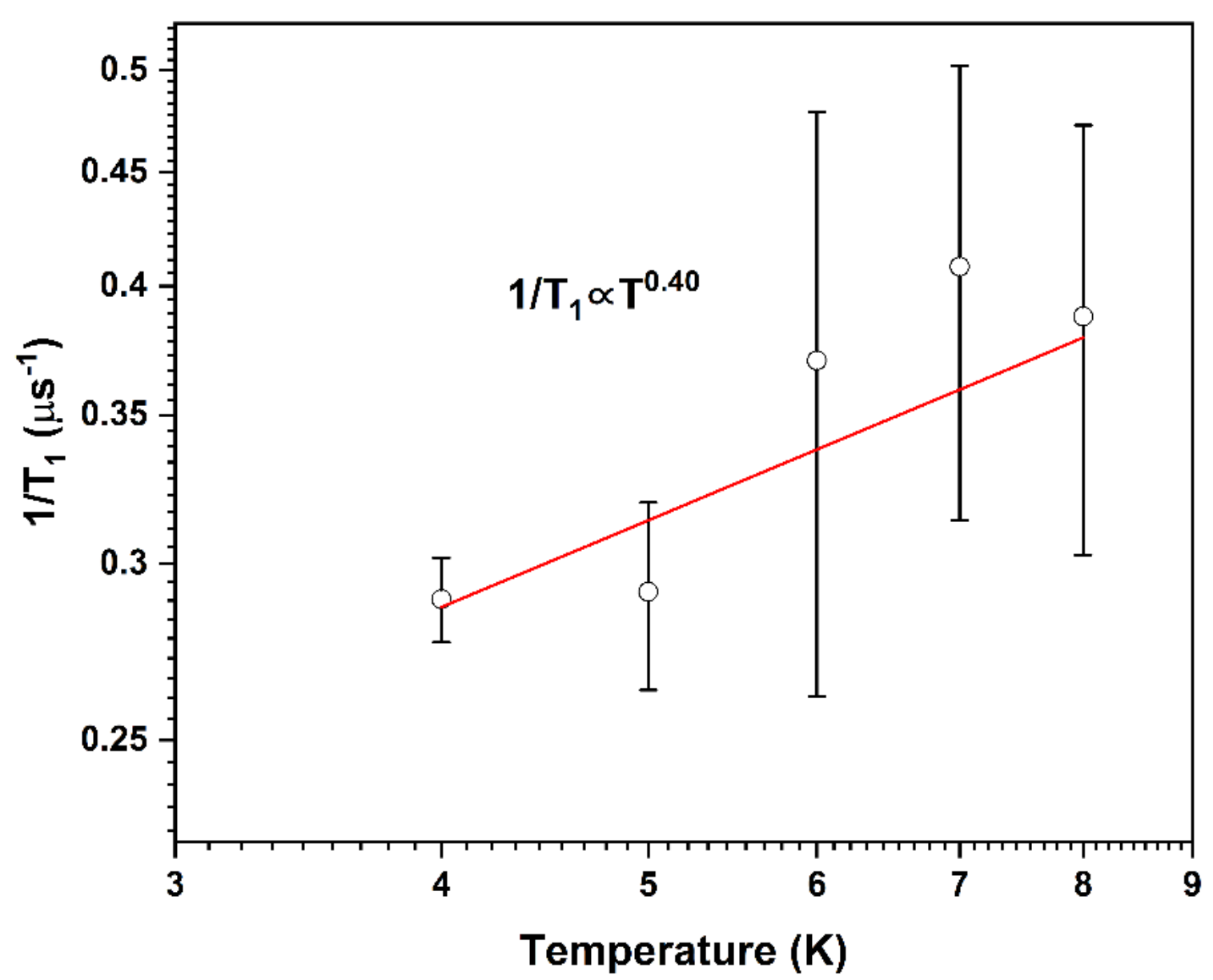

Figure S27. Plot of log(1/T_1) vs. log(temperature) for **4-Tb⁴⁺THF** and corresponding linear fit (red). The dependence of 1/T_1 as a function of temperature extracted from the fit is given in the plot.

***T_m* Mechanistic Fits.** Sufficient data is available for **1-Eu²⁺**, **2-Gd³⁺**, and **3-Gd³⁺** to enable fitting of the T_m data to extract information regarding the mechanisms driving the phase memory relaxation. Beginning with the model for the coherence lifetime of the central transition proposed by Wilson *et al.*:[23]

$$\frac{1}{T_m} = A_1 \omega_{dd}(\bar{r}) \sum_{m=-S}^{S-1} \sum_{m'=-S+1}^{S} W_{m,m'} n_m n_{m'} + A_2 \frac{\omega_{dd}(\bar{r})}{T_1} + \frac{C}{T_1} + \Gamma \qquad \text{(S4)}$$

Where $1/T_m$ and $1/T_1$ are the rates of the phase memory and spin-lattice relaxations respectively, $\omega_{dd}(\bar{r})$ is the average nearest-neighbor dipolar coupling strength and $W_{m,m'}$ is the matrix element of an electronic spin "flip-flop" between two arbitrary spin projection states (m and m') on different sites weighted by the Boltzmann population of their Zeeman-split energies (n_m and $n_{m'}$) and summed over all valid spin projection combinations. The parameters A_1, A_2, and C weight the individual contributions of the "ubiquitous flip-flop", "T_1 induced" neighbor spin flips (off-site), and direct T_1 induced relaxation (on-site) processes and Γ is some residual relaxation rate. In the absence of variable concentration data, the off-site and on-site T_1 contributions cannot be distinguished, meaning A_2 and C cannot be uniquely determined. Considering this and neglecting contributions from other processes (Γ), Eqn. S4 can be recast as:

$$\frac{1}{T_m} = A_1 \omega_{dd}(\bar{r}) \sum_{m=-S}^{S-1} \sum_{m'=-S+1}^{S} W_{m,m'} n_m n_{m'} + \frac{F}{T_1} \qquad \text{(S5)}$$

Where the parameter F now weights both on- and off-site T_1 contributions to phase memory relaxation. Fits of the $1/T_m$ data acquired for **1-Eu^{2+}**, **2-Gd^{3+}**, and **3-Gd^{3+}** to Eqn. S5 can then be readily performed and the results are summarized in Table S6 and shown as plots of $1/T_m$ vs. $1/T_1$ in Figures S28-S30.

Table S6. T_m fitting parameters for **1-Eu^{2+}**, **2-Gd^{3+}**, and **3-Gd^{3+}**, based on Eqn. S5 and $\Delta\ ^8S$ parameter.

Sample	A_1 (10^{-3})	F	$\Delta\ ^8S$ (cm^{-1})[a]
1-Eu^{2+}	5.0(8)	1.7(2)	0.696
2-Gd^{3+}	1.2(6)	3.74(17)	1.115
3-Gd^{3+}	2.7(4)	2.47(10)	0.179

[a]Taken from Table S3

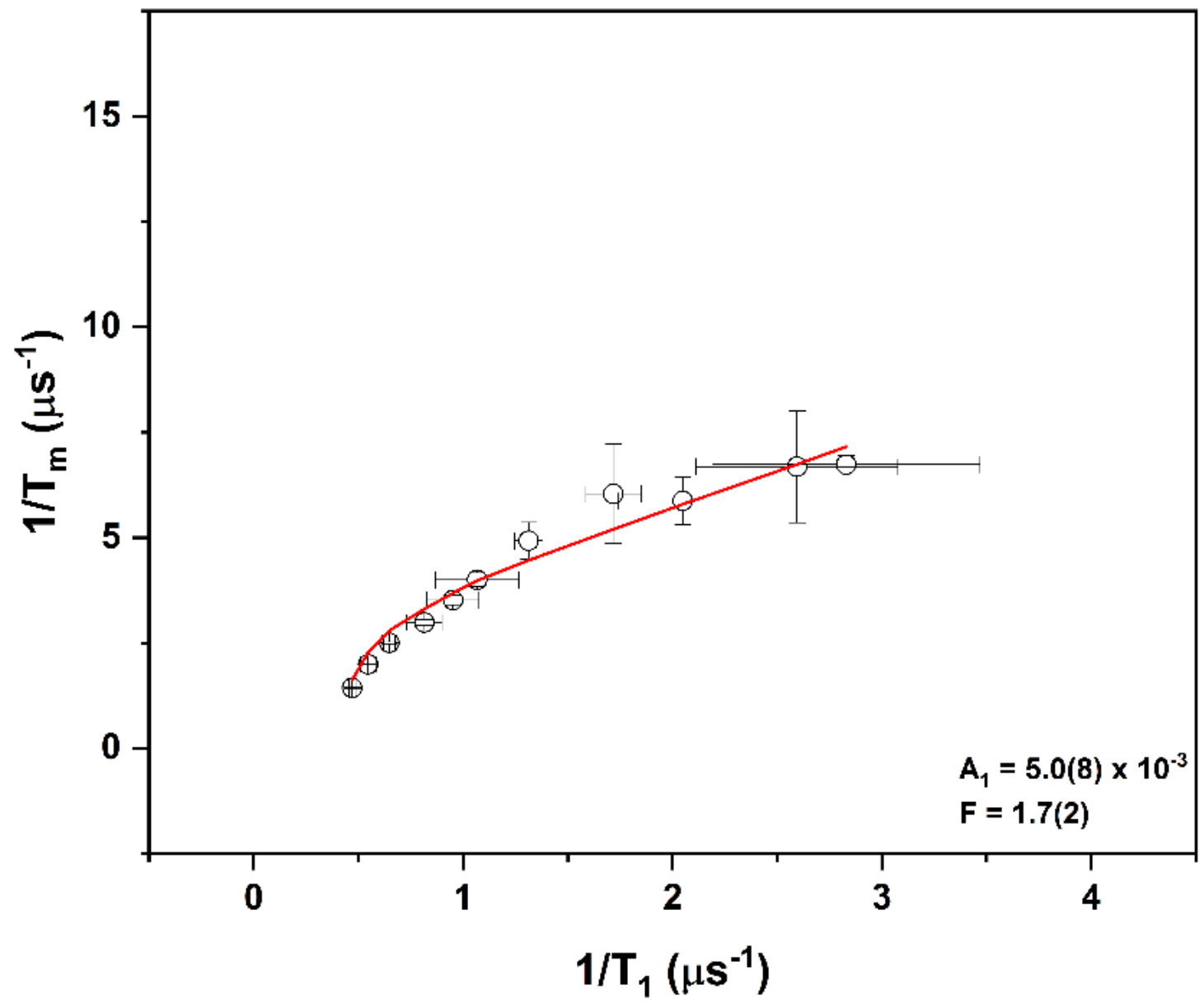

Figure S28. Plot of $1/T_m$ vs. $1/T_1$ (with errors) for **1-Eu^{2+}**, corresponding fit (red) from Eqn. S5, and fit parameters.

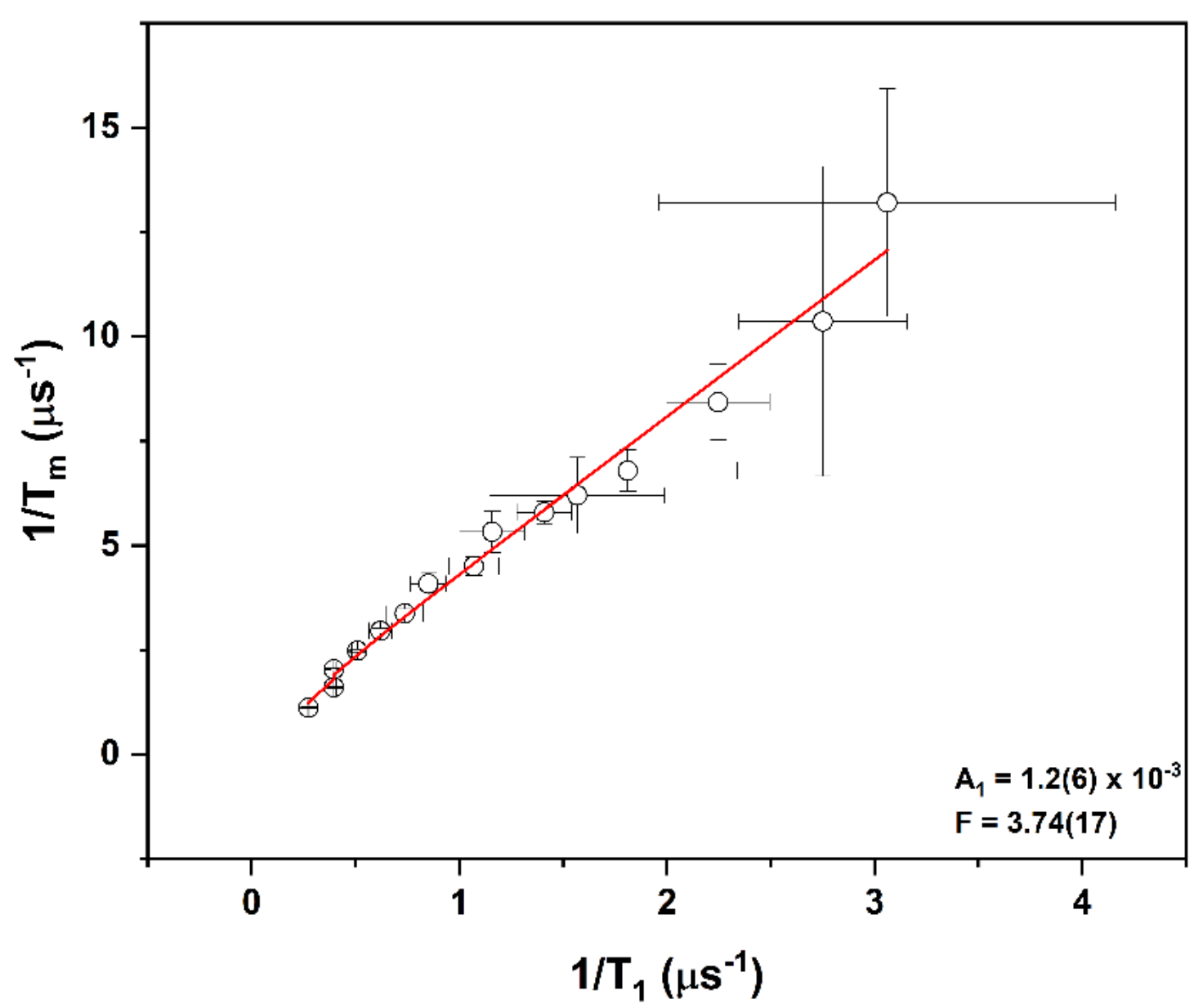

Figure S29. Plot of $1/T_m$ vs. $1/T_1$ (with errors) for **2-Gd^{3+}**, corresponding fit (red) from Eqn. S5, and fit parameters.

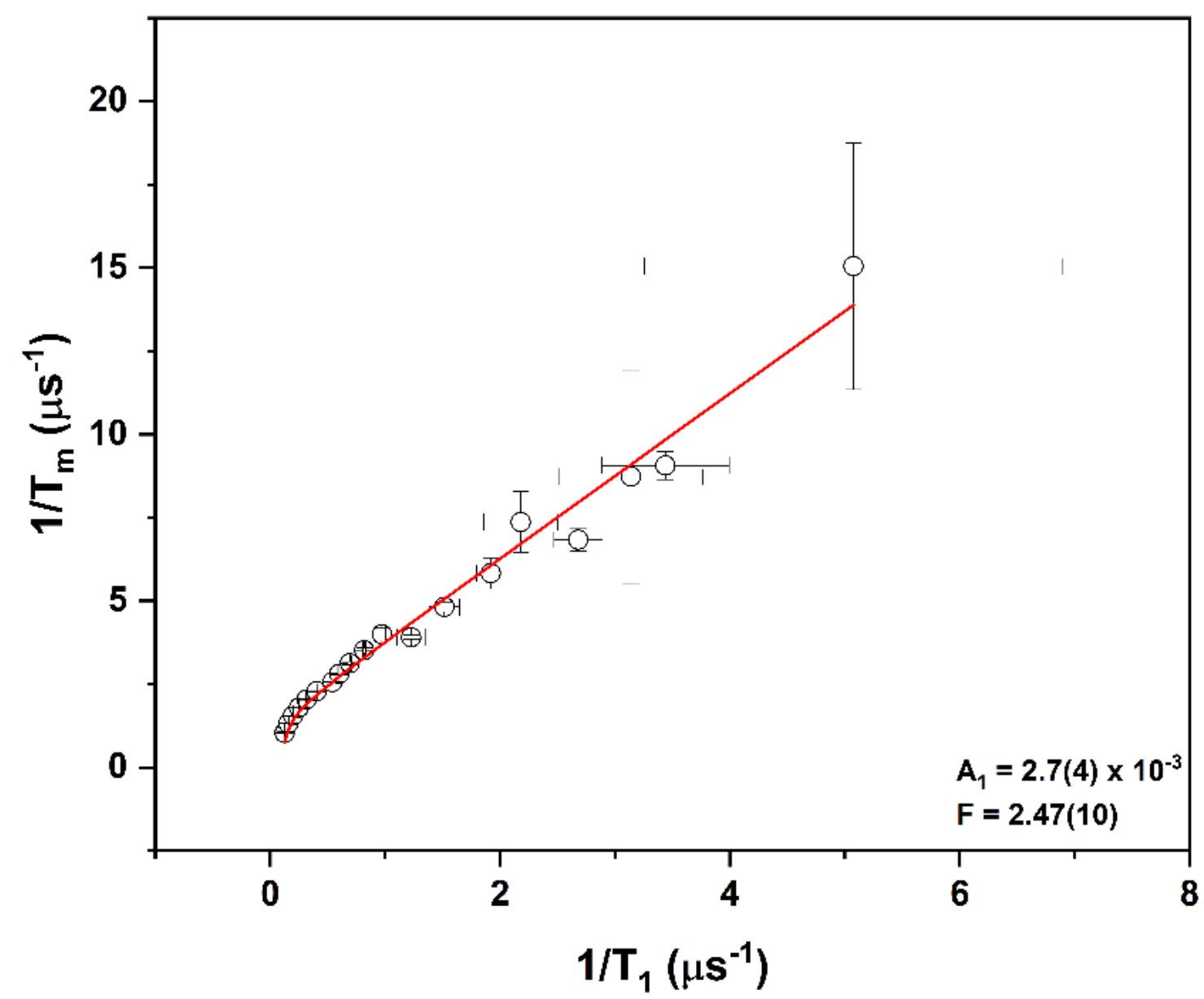

Figure S30. Plot of $1/T_m$ vs. $1/T_1$ (with errors) for **3-Gd^{3+}**, corresponding fit (red) from Eqn. S5, and fit parameters.

To determine the expected 5 K T_m values due to only T_1 contributions, the linear regimes in Figures S28-S30 were extrapolated down to 5 K by constructing a line extending from the fastest (highest temperature) fitted ($1/T_1$,$1/T_m$) point to the slowest $1/T_1$ value for each plot and the low temperature regions of this extrapolation are shown in Figures S31-33.

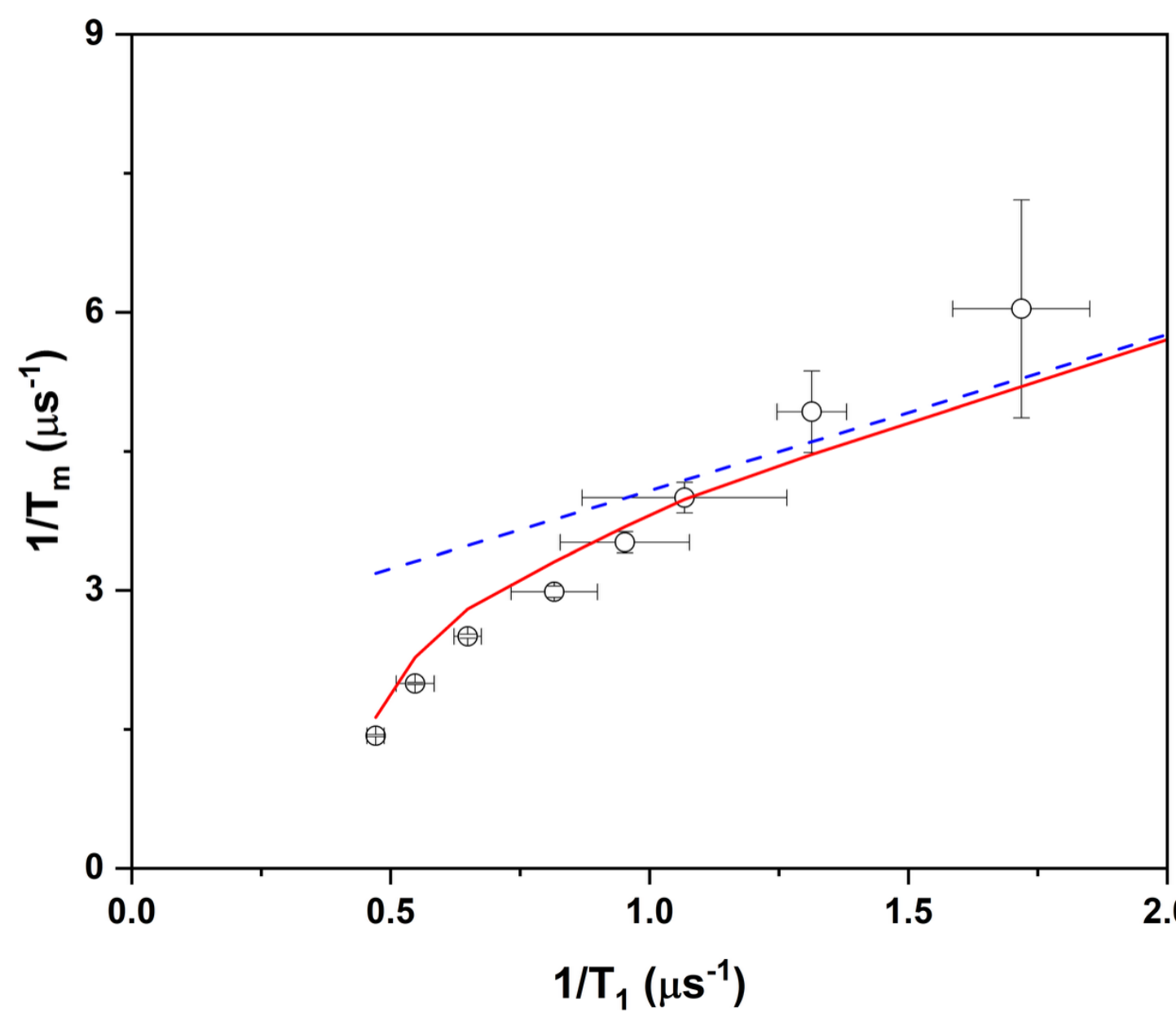

Figure S31. Low temperature plot of $1/T_m$ vs. $1/T_1$ (with errors) for **1-Eu^{2+}**, corresponding fit (red) and extrapolation (blue). Extrapolated value for $1/T_m$ at 5 K is 3.18 μs^{-1}.

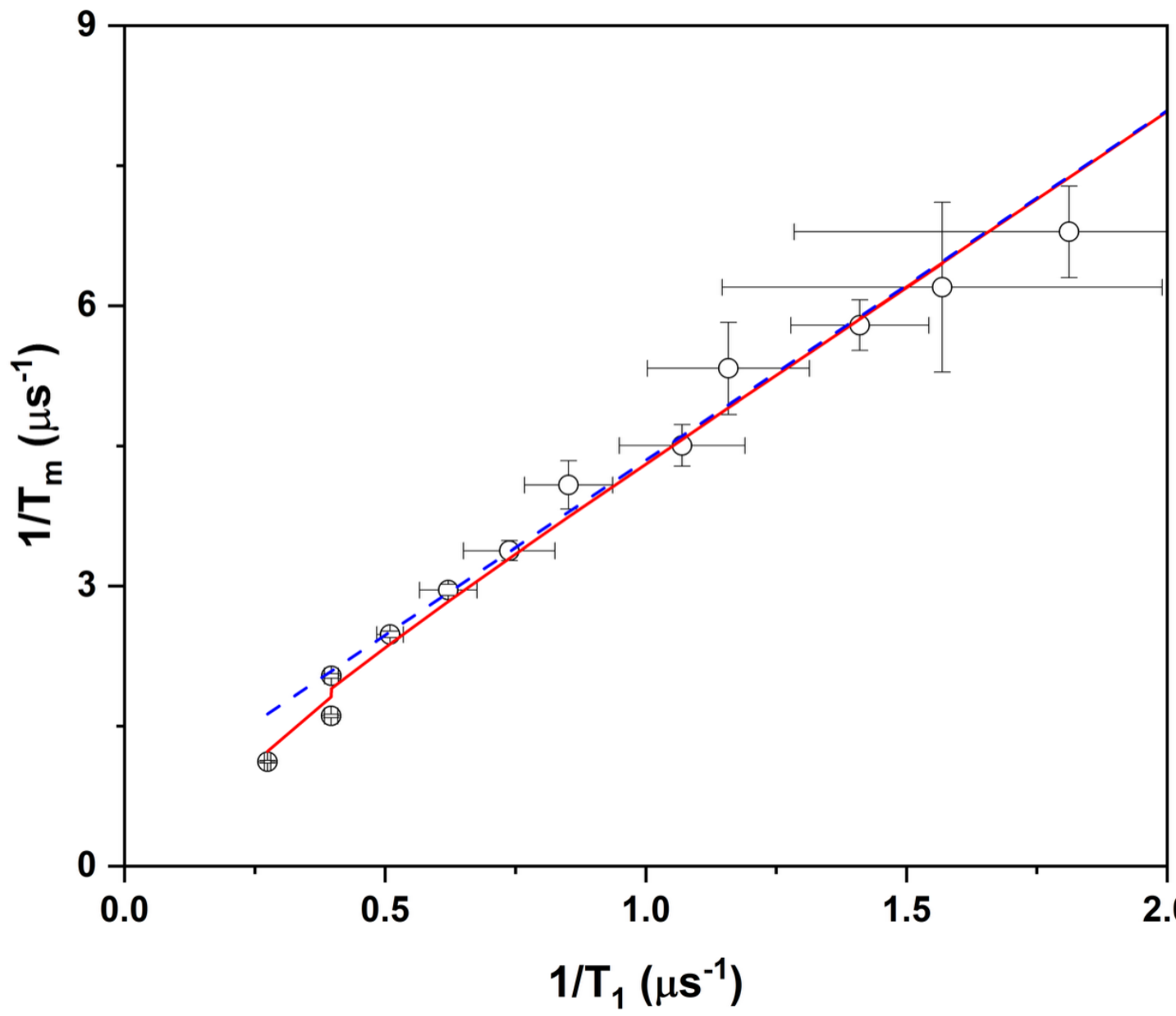

Figure S32. Low temperature plot of 1/T_m vs. 1/T_1 (with errors) for **2-Gd^{3+}**, corresponding fit (red) and extrapolation (blue). Extrapolated value for 1/T_m at 5 K is 1.63 μs^{-1}.

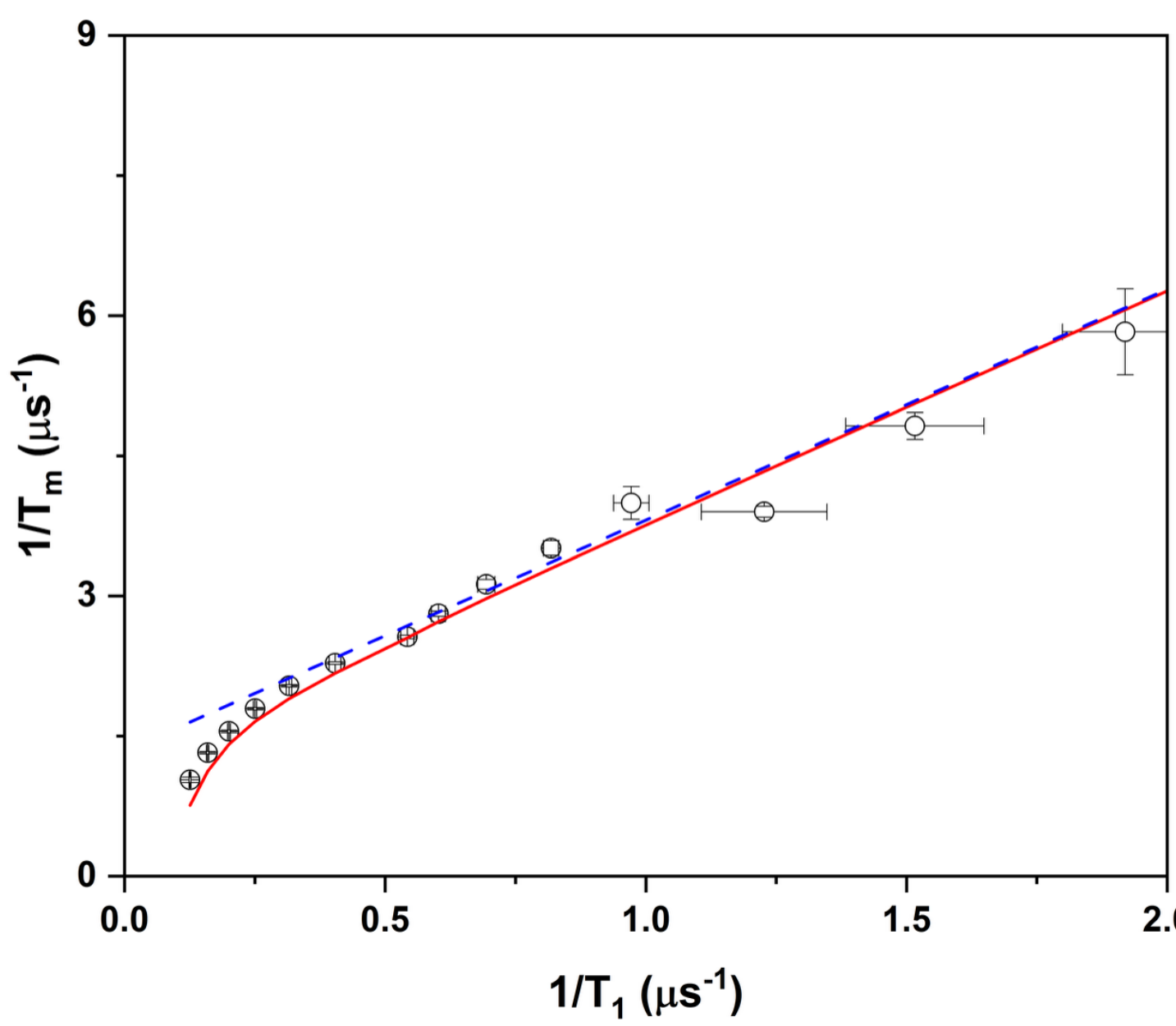

Figure S33. Low temperature plot of 1/T_m vs. 1/T_1 (with errors) for **3-Gd^{3+}**, corresponding fit (red) and extrapolation (blue). Extrapolated value for 1/T_m at 5 K is 1.65 μs^{-1}.

Trends in T_1 and T_m at 5 K. To examine potential trends in T_1 and T_m across analytes as a function of the changing ZFS parameters, the measured values at 5 K were plotted as a function of $\Delta\ {}^8S$. (Figures S34-S35).

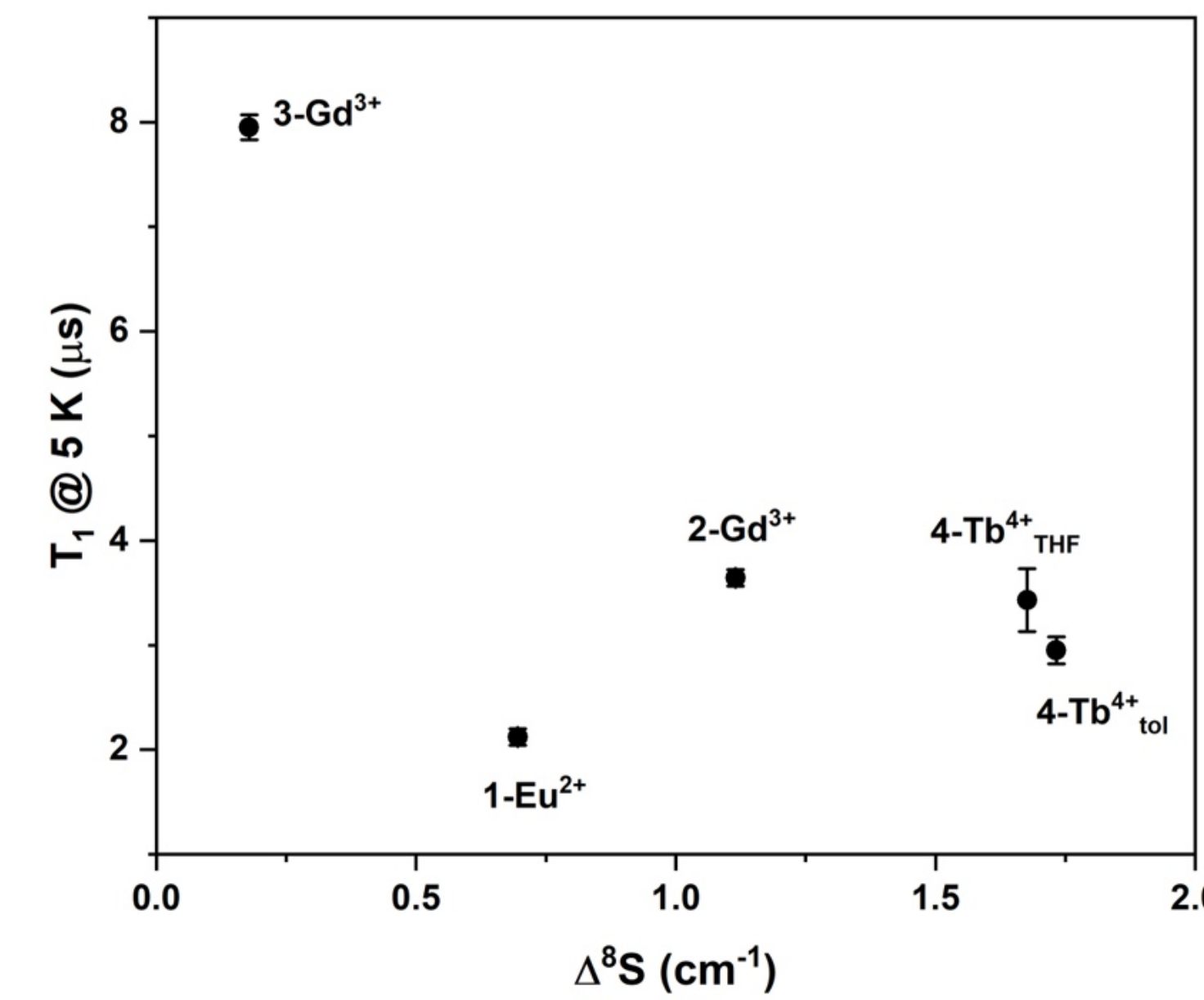

Figure S34. Plot of 5 K T_1 (with errors) vs. Δ ^8S for studied analytes. No obvious correlation is apparent in the measured data.

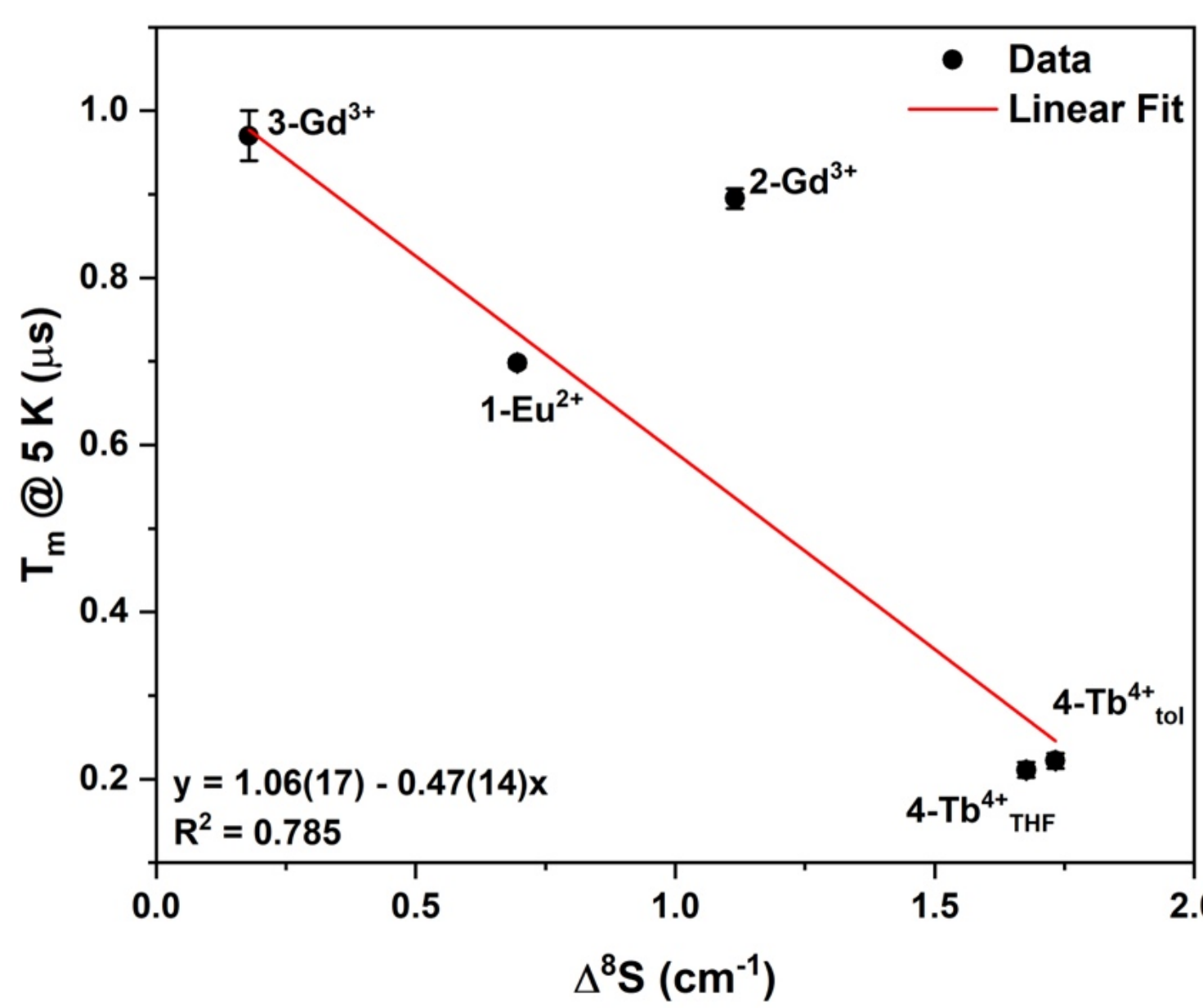

Figure S35. Plot of 5 K T_m (with errors) vs. Δ ^8S for studied analytes**,** corresponding linear fit (red) and relevant fit parameters.

Replotting the same values neglecting the outlier **2-Gd^{3+}** affords a significantly improved linear fit (Figure S36).

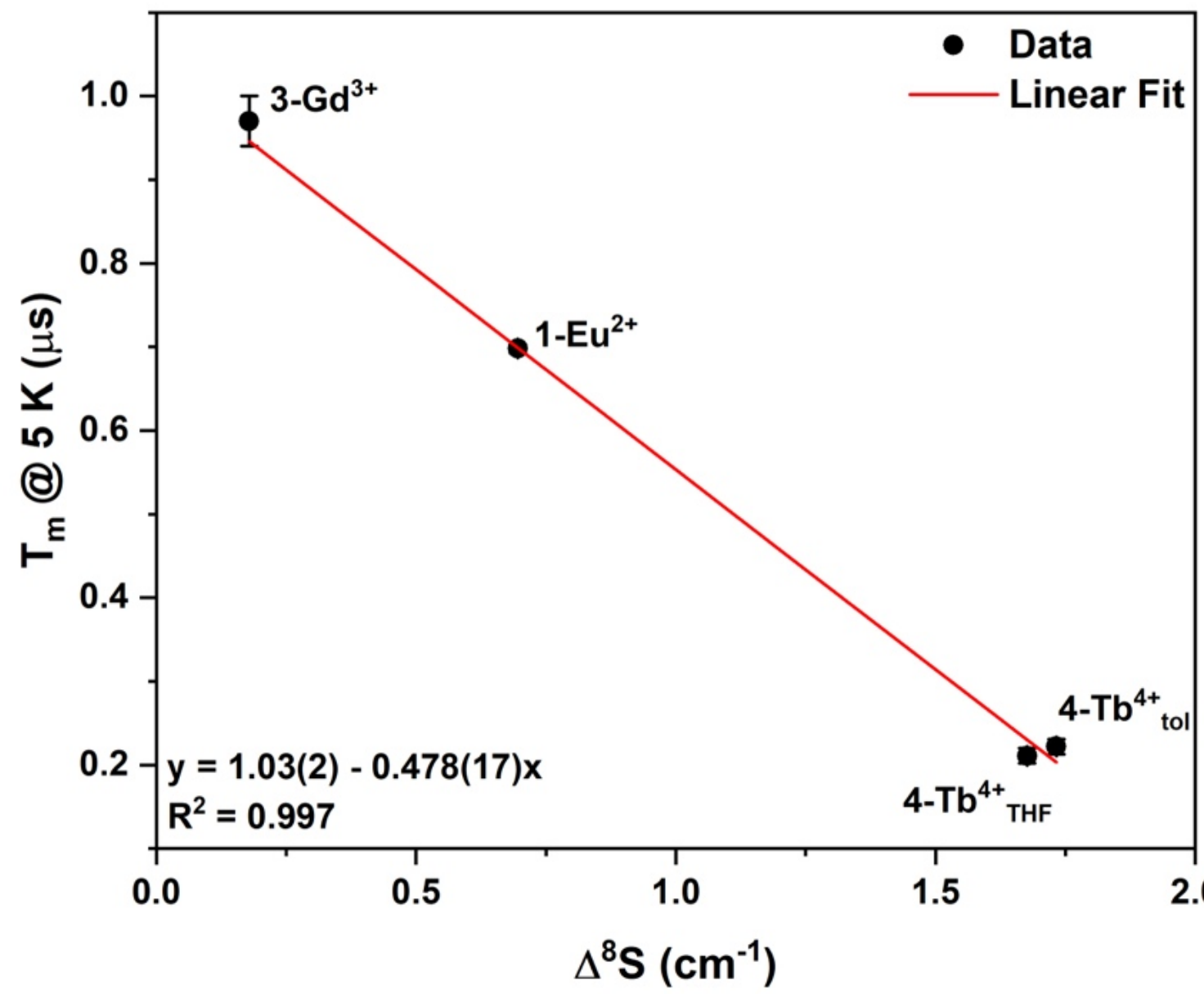

Figure S36. Plot of 5 K T_m (with errors) vs. $\Delta\ ^8S$ for **1-Eu²⁺**, **3-Gd³⁺**, **4-Tb⁴⁺tol**, and **4-Tb⁴⁺THF**, corresponding linear fit (red) and relevant fit parameters.

To examine any correlation between 5 K T_m and T_1 values that would be consistent with T_1 driven decoherence at this temperature, the two parameters are plotted against each other (Figure S37).

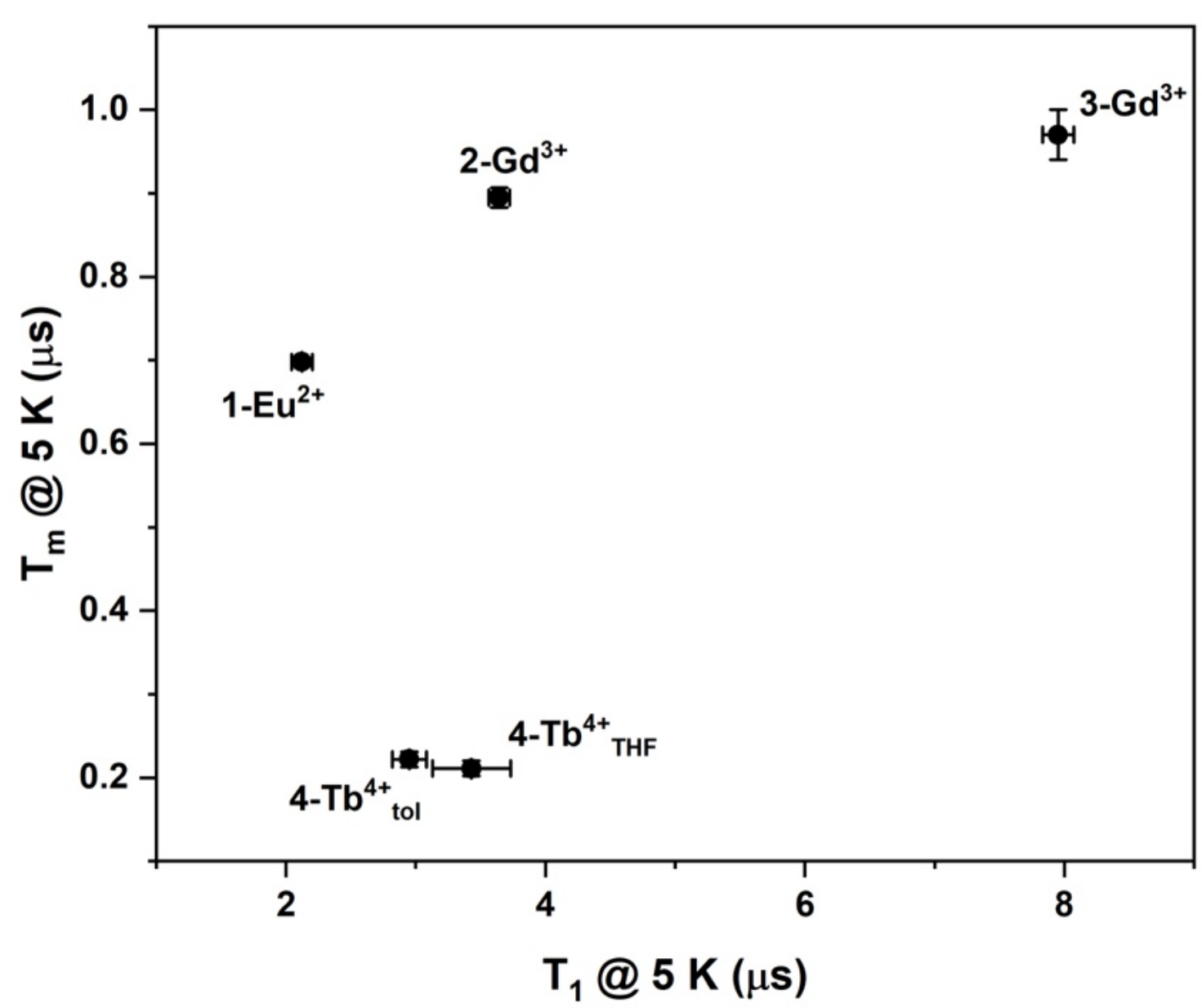

Figure S37. Plot of 5 K T_m vs. T_1 with measurement errors shown for studied analytes.

References

(1) Rice, N. T.; Popov, I. A.; Russo, D. R.; Bacsa, J.; Batista, E. R.; Yang, P.; Telser, J.; La Pierre, H. S. Design, Isolation, and Spectroscopic Analysis of a Tetravalent Terbium Complex. *J. Am. Chem. Soc.* **2019**, *141* (33), 13222-13233. DOI: 10.1021/jacs.9b06622.

(2) Gompa, T. P.; Greer, S. M.; Rice, N. T.; Jiang, N.; Telser, J.; Ozarowski, A.; Stein, B. W.; Pierre, H. S. L. High-Frequency and -Field Electron Paramagnetic Resonance Spectroscopic Analysis of Metal-Ligand Covalency in a $4f^7$ Valence Series (Eu^{2+}, Gd^{3+}, Tb^{4+}). *Inorg. Chem.* **2021**, *60*, 9064-9073. DOI: 10.1021/acs.inorgchem.1c01062.

(3) Watson, P. L.; Tulip, T. H.; Williams, I. Defluorination of perfluoroolefins by divalent lanthanoid reagents: activating carbon-fluorine bonds. *Organometallics* **1990**, *9* (7), 1999-2009. DOI: 10.1021/om00157a006.

(4) Groom, C. R.; Bruno, I. J.; Lightfoot, M. P.; Ward, S. C. The Cambridge Structural Database. *Acta Cryst. B* **2016**, *72* (2), 171-179. DOI: 10.1107/s2052520616003954.

(5) *FinalCIF, V125*; https://dkratzert.de/finalcif.html.

(6) Macrae, C. F.; Sovago, I.; Cottrell, S. J.; Galek, P. T. A.; Mccabe, P.; Pidcock, E.; Platings, M.; Shields, G. P.; Stevens, J. S.; Towler, M.; Wood, P. A. *Mercury 4.0*: from visualization to analysis, design and prediction. *J. Appl. Cryst.* **2020**, *53* (1), 226-235. DOI: 10.1107/s1600576719014092.

(7) *POV-Ray, v3.7.0*; http://www.povray.org/.

(8) Lukens, W. W.; Minasian, S. G.; Booth, C. H. Strengths of covalent bonds in LnO_2 determined from O K-edge XANES spectra using a Hubbard model. *Chem. Sci.* **2023**, *14* (44), 12784-12795. DOI: 10.1039/d3sc03304j.

(9) *SAINT, v8.40B*; Madison, Wisconsin, USA.

(10) Krause, L.; Herbst-Irmer, R.; Sheldrick, G. M.; Stalke, D. Comparison of silver and molybdenum microfocus X-ray sources for single-crystal structure determination. *J. Appl. Cryst.* **2015**, *48* (1), 3-10. DOI: 10.1107/s1600576714022985.

(11) Sheldrick, G. M. *SHELXT*– Integrated space-group and crystal-structure determination. *Acta Cryst. A* **2015**, *71* (1), 3-8. DOI: 10.1107/s2053273314026370.

(12) Sheldrick, G. M. Crystal structure refinement with *SHELXL*. *Acta Cryst. C* **2015**, *71* (1), 3-8. DOI: 10.1107/s2053229614024218.

(13) Dolomanov, O. V.; Bourhis, L. J.; Gildea, R. J.; Howard, J. A. K.; Puschmann, H. OLEX2: a complete structure solution, refinement and analysis program. *J. Appl. Cryst.* **2009**, *42* (2), 339-341. DOI: 10.1107/S0021889808042726.

(14) Höpfl, H. The tetrahedral character of the boron atom newly defined—a useful tool to evaluate the N→B bond. *J. Organomet. Chem.* **1999**, *581* (1), 129-149. DOI: 10.1016/S0022-328X(99)00053-4.

(15) Boggiano, A. C.; Chowdhury, S. R.; Roy, M. D.; Bernbeck, M. G.; Greer, S. M.; Vlaisavljevich, B.; La Pierre, H. S. A Four-Coordinate Pr^{4+} Imidophosphorane Complex. *Angew. Chem. Int. Ed.* **2024**, *63* (43), e202409789. DOI: 10.1002/anie.202409789.

(16) Bain, G. A.; Berry, J. F. Diamagnetic Corrections and Pascal's Constants. *J. Chem. Ed.* **2008**, *85* (4), 532. DOI: 10.1021/ed085p532.

(17) Kahn, O. *Molecular Magnetism*; Dover Publications, 2021.

(18) Cruickshank, P. A. S.; Bolton, D. R.; Robertson, D. A.; Hunter, R. I.; Wylde, R. J.; Smith, G. M. A kilowatt pulsed 94 GHz electron paramagnetic resonance spectrometer with high concentration sensitivity, high instantaneous bandwidth, and low dead time. *Review of Scientific Instruments* **2009**, *80* (10), 103102. DOI: 10.1063/1.3239402.

(19) Subramanya, M. V. H.; Marbey, J.; Kundu, K.; McKay, J. E.; Hill, S. Broadband Fourier-Transform-Detected EPR at W-Band. *Appl. Magn. Reson.* **2023**, *54* (1), 165-181. DOI: 10.1007/s00723-022-01499-3.

(20) Epel, B.; Gromov, I.; Stoll, S.; Schweiger, A.; Goldfarb, D. Spectrometer manager: A versatile control software for pulse EPR spectrometers. *Concepts Magn. Reson., Part B Magn. Reson. Eng.* **2005**, *26B* (1), 36-45. DOI: 10.1002/cmr.b.20037.
(21) Stoll, S.; Schweiger, A. EasySpin, a comprehensive software package for spectral simulation and analysis in EPR. *J. Magn. Reson.* **2006**, *178* (1), 42-55. DOI: 10.1016/j.jmr.2005.08.013.
(22) Newville, M.; Otten, R.; Nelson, A.; Stensitzki, T.; Ingargiola, A.; Allan, D.; Fox, A.; Carter, F.; Michal; Osborn, R.; Pustakhod, D.; Weigand, S.; Ineuhaus; Aristov, A.; Glenn; Mark; mgunyho; Deil, C.; Hansen, A. L. R.; Pasquevich, G.; Foks, L.; Zobrist, N.; Frost, O.; Stuermer; Jaskula, J.-C.; Caldwell, S.; Eendebak, P.; Pompili, M.; Nielsen, J. H.; Persaud, A. lmfit/lmfit-py: 1.3.1. **2024**. DOI: 10.5281/zenodo.10998841.
(23) Wilson, C. B.; Qi, M.; Han, S.; Sherwin, M. S. Gadolinium Spin Decoherence Mechanisms at High Magnetic Fields. *J. Phys. Chem. Lett.* **2023**, *14* (47), 10578-10584. DOI: 10.1021/acs.jpclett.3c01847.
